# UMAP reveals cryptic population structure and phenotype heterogeneity in large genomic cohorts

**DOI:** 10.1371/journal.pgen.1008432

**Published:** 2019-11-01

**Authors:** Alex Diaz-Papkovich, Luke Anderson-Trocmé, Chief Ben-Eghan, Simon Gravel

**Affiliations:** 1 Quantitative Life Sciences, McGill University, Montreal, Québec, Canada; 2 McGill University and Genome Quebec Innovation Centre, Montreal, Québec, Canada; 3 Department of Human Genetics, McGill University, Montreal, Québec, Canada; University of Pennsylvania, UNITED STATES

## Abstract

Human populations feature both discrete and continuous patterns of variation. Current analysis approaches struggle to jointly identify these patterns because of modelling assumptions, mathematical constraints, or numerical challenges. Here we apply uniform manifold approximation and projection (UMAP), a non-linear dimension reduction tool, to three well-studied genotype datasets and discover overlooked subpopulations within the American Hispanic population, fine-scale relationships between geography, genotypes, and phenotypes in the UK population, and cryptic structure in the Thousand Genomes Project data. This approach is well-suited to the influx of large and diverse data and opens new lines of inquiry in population-scale datasets.

## Author summary

The demographic history of human populations features varying geographic and social barriers to mating. Over time, these barriers have led to varying levels of genetic relatedness among individuals. This population structure is informative about human history, and can have a significant impact on studies of medical genetics. Because population structure depends on myriad demographic, ecological, and social forces, a priori visualization is useful to identify subtle patterns of population structure. We use a dimension reduction method—UMAP—to visualize population structure in three genomic datasets and find previously unobserved patterns, revealing fine-scale population structure and illustrating differences between groups in traits such as white blood cell count, height, and FEV1, a measure of lung function. Using UMAP is computationally efficient and can identify fine-scale population structure in large population datasets. We find it particularly useful to reveal phenotypic variation among genetically related populations, and recommend it is a complement to principal component analysis in primary data visualization.

## Introduction

Questions in medicine, anthropology, and related fields hinge on interpreting the deluge of genomic data provided by modern high-throughput sequencing technologies. Because genomic datasets are high-dimensional, their interpretation requires statistical methods that can comprehensively condense information in a manner that is understandable to researchers and minimizes the amount of data that is sacrificed. Both model-based and model-agnostic approaches to summarize data have played important roles in shaping our understanding of the evolution of our species [e.g., [1–5]].

Here we will focus on nonparametric approaches to visualize relatedness patterns among individuals within populations. If we consider unphased single nucleotide polymorphism (SNP) data, an individual genome can be represented as a sequence of integers corresponding to the number of copies of the alleles carried by the individual at each of the *L* SNPs for which genotypes are available, with *L* ranging from hundreds of thousands to hundreds of millions. Since each individual is represented as an *L*-dimensional vector, dimension reduction methods are needed to visualize the data.

Principal component analysis (PCA) is often the first dimensional reduction tool used for genomic data. It identifies and ranks directions in genotype space that explain most-to-least variance among individuals. Positions of individuals along directions of highest variance can then be used to summarize individual genotypes. PCA coordinates have natural genealogical interpretations in terms of expected times to a most recent common ancestor (TMRCA) [6], and are used empirically to reveal admixture [7], continuous isolation-by-distance [8, 9], as well as technical artefacts. PCA coordinates are particularly well-suited to correct for population structure in GWAS [4].

The amount of information encoded in the highest-variance PCs increases slowly with sample size, so researchers typically examine multiple two-dimensional projections to lower-variance PCs to explore data. In this process, finer features of the data may be hidden by the projections or hard to interpret. To display finer features of the data in a two dimensional figure, we can use non-linear transformations that emphasize the local structure of the data. A popular method for such visualization is t-distributed stochastic neighbour embedding (t-SNE) [10]. t-SNE has been used before to visualize SNPs [11]. Using data from the 1000 Genomes Project (1KGP) [12], it groups individuals corresponding roughly to their continent of origin, with smaller ethnic sub-groups visible within the larger continental clusters [13]. However, t-SNE struggles with very large datasets, when a large number of locally optimal configurations make convergence to a globally satisfying solution difficult.

Uniform Manifold Approximation and Projection (UMAP) is a dimension reduction technique designed to model and preserve the high-dimensional topology of data points in the low-dimensional space [14]. With genotype data, UMAP creates a neighbourhood around each individual’s genetic coordinates and identifies a pre-selected number of neighbours to build high-dimensional manifolds. The end result is a patchwork of low-dimensional representations of neighbourhoods that groups genetically similar individuals together on a local scale while better preserving long-range topological connections to more distantly related individuals. The method has been successfully applied to single-cell RNA sequencing datasets [15].

Non-linear dimension reduction methods tend to be computationally intensive. A common practice to reduce this burden is to first apply PCA to data, and perform dimensional reduction on data projected to leading principal components (PCs). In addition to being computationally advantageous, this discards noise that can confound non-linear approaches: population structure arising from *n* isolated randomly-mating demes can be described by the leading *n* − 1 PCs, with the following PCs describing stochastic variation in relatedness [4]. Selecting the leading PCs therefore has potential to extract meaningful population structure while filtering out stochastic noise. We explore different strategies to pre-process the data and investigate discrete and continuous population structure patterns present in large datasets of human genotypes: the 1KGP, the Health and Retirement Study (HRS) [16], and the UK BioBank (UKBB) [17], and compare UMAP’s performance to t-SNE.

## Results

### Fine-scale visualization of the 1KGP dataset

**Fig 1:**
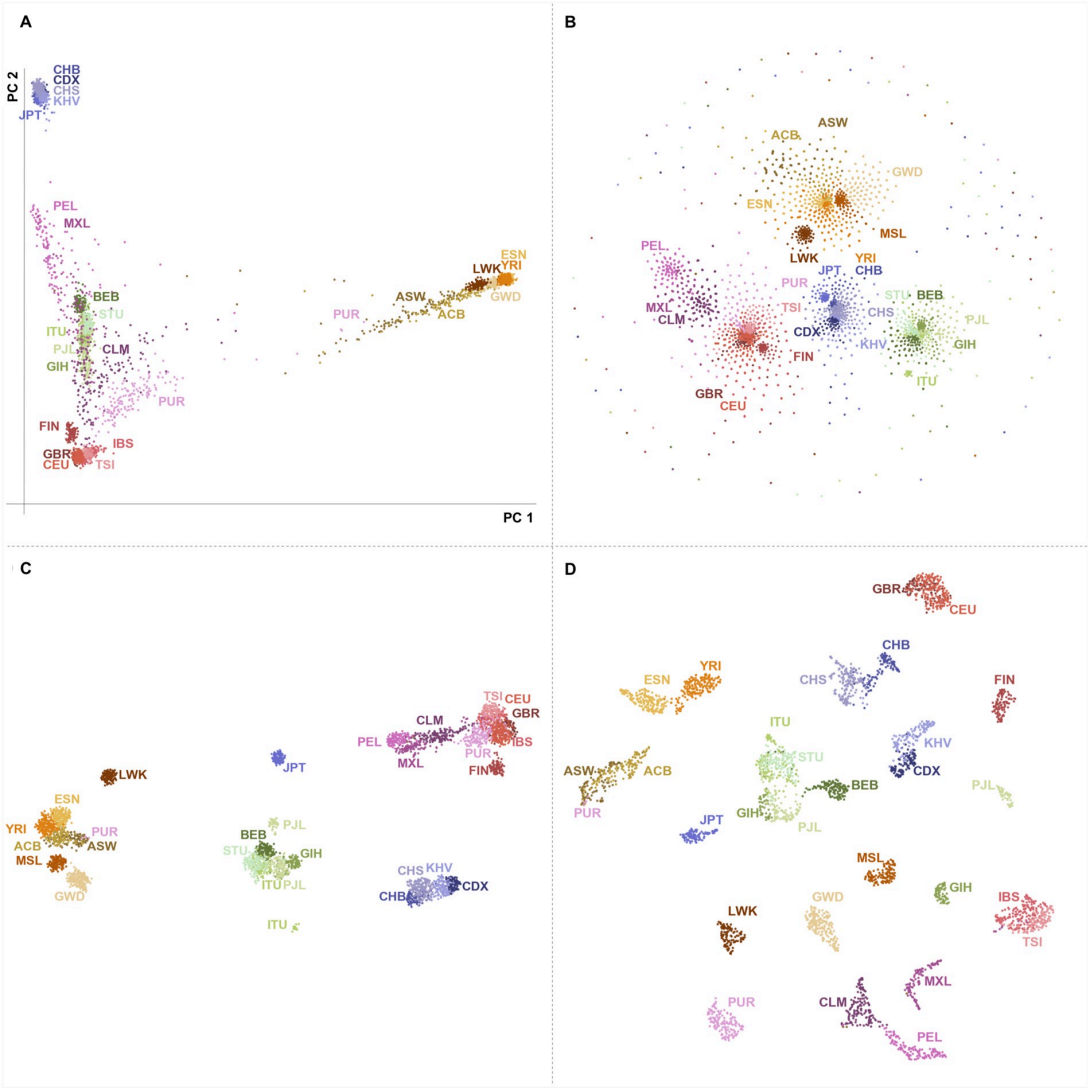
Four methods of dimension reduction of 1KGP genotype data with population labels. (**A**) PCA maps individuals in a triangle with vertices corresponding to African, Asian, and European continental ancestry. Discarding lower-variance PCs leads to overlap of populations with no close affinity, such as Central and South American populations with South Asians. (**B**) t-SNE forms groups corresponding to continents, with some overlap between European and Central and South American people. Smaller subgroups are visible within continental clusters. The cloud of peripheral points results from the method’s poor convergence. (**C**) UMAP forms distinct clusters related to continent with clearly defined subgroups. Japanese, Finnish, Luhya, and some Punjabi and Telugu populations form separate clusters consistent with their population history [12]. (**D**) UMAP on the first 15 principal components forms fine-scale clusters for individual populations. Groups closely related by ancestry or geography, such as African Caribbean/African American, Spanish/Italian, and Kinh/Dai populations cluster together. Results using t-SNE on principal components are presented in S1 Fig. Axes in UMAP and t-SNE are arbitrary. Since the algorithms prioritize local distances, long distances between clusters are not meaningful. ACB, African Caribbean in Barbados; ASW, African Ancestry in Southwest US; BEB, Bengali; CDX, Chinese Dai; CEU, Utah residents with Northern/Western European ancestry; CHB, Han Chinese; CHS, Southern Han Chinese; CLM, Colombian in Medellin, Colombia; ESN, Esan in Nigeria; FIN, Finnish; GBR, British in England and Scotland; GWD, Gambian; GTH, Gujarati; IBS, Iberian in Spain; ITU, Indian Telugu in the UK; JPT, Japanese; KHV, Kinh in Vietnam; LWK, Luhya in Kenya; MSL, Mende in Sierra Leone; MXL, Mexican in Los Angeles, California; PEL, Peruvian; PJL, Punjabi in Lahore, Pakistan; PUR, Puerto Rican; STU, Sri Lankan Tamil in the UK; TSI, Toscani in Italy; YRI, Yoruba in Nigeria.

The 1KGP contains genotype data of 3,450 individuals from 26 relatively distinct labeled populations [12]. Fig 1 shows visualizations using PCA, t-SNE, UMAP, and UMAP with PCA pre-processing. Using UMAP and t-SNE on the genotype data presents clusters that are roughly grouped by continent, with UMAP showing a clear hierarchy of population and continental clusters, whereas t-SNE fails to assign many individuals to population clusters. Using either method on the top principal components leads to distinct population clusters and less defined continental structure. Adding more components results in progressively finer clusters until approximately 20 populations appear using 15 components; adding further components converges to results similar to using the entire genotype data (see S1, S2, and S3 Figs). To investigate the population information contained in low-variance PCs, we performed UMAP on data projected onto PCs 100 to 3450 (i.e., without information about the leading 99 PCs). S4 Fig shows that population structure is still clearly visible.

Focusing on UMAP with the leading 15 principal components (Fig 1D), several population clusters reflect shared ancestries. British individuals from England and Scotland form a cluster mixed with those from Utah who claim Northern and Western European ancestry. Toscani and Iberian individuals form a cluster reflecting their Mediterranean heritage. African Americans in the Southwest US, African Caribbean individuals in Barbados, and some Puerto Ricans also form a cluster. Three East Asian clusters appear: one is largely Han and Southern Han individuals, another is comprised of the Chinese Dai in southern China and the Kinh from Vietnam, and the third is the Japanese population. Other clusters are comprised of Colombians and Peruvians, the Esan and Yoruba populations of Nigeria, and several South Asian populations.

Within population clusters, family members were projected near each other within broader population groups. When UMAP was parameterized to use only 5 nearest neighbours, however, families often formed distinct clusters (S5 Fig): Using few neighbours to build a manifold emphasizes closer relatedness.

A few individuals cluster with populations different from their label: some Mexican individuals cluster with Spanish and Italian populations; some Puerto Rican individuals cluster with African American and Caribbean populations; and one Gambian individual clusters with the Mende of Sierra Leone. Two populations form multiple clusters: Gujarati Indians in Houston, Texas and Punjabi people in Lahore, Pakistan. This clustering effect is robust to the number of components considered (S2 Fig). Differentiation in the 1KGP Gujarati population has been previously identified through a PCA restricted to the Gujarati [18]. Following a preprint version of the present article, 23andme released a statement [19] arguing that one of the two clusters could be traced, via 23andMe participant recontact to individuals from a group in Western India with shared ancestry and patronym. [D. Poznik, 23andme, personal communication, and [19]].

### Admixed individuals fall along a genetic continuum

The 1KGP sampled individuals from relatively distinct populations, so the data are more likely to form clusters. Most medical cohorts, however, comprise larger numbers of individuals sampled across extended geographical areas. The HRS contains genotype data of 12,454 Americans from a variety of backgrounds. Using UMAP on the first 10 principal components, we demonstrate projections that present a collection of sub-populations and a continuum of genetic variation.

The HRS forms several large clusters, reflecting both ethnicity (S6 Fig) and admixture proportions (S7 Fig). Gradients in admixture proportion are visible within the predominantly Hispanic cluster, but not within the predominantly Black cluster, perhaps because the variance in ancestry proportions is greater among Hispanics. The “White Not Hispanic” (WNH) group forms several interconnected clusters, and these do not correspond to broad geographical areas (S8 Fig). By generating the PC axes and UMAP embedding for the HRS data in S6 Fig, and projecting the 1KGP data onto it, we reveal substructure within the Hispanic cluster, groupings of Finnish individuals within the WNH groups, as well as Italian and Spanish individuals grouping near the White Hispanic population (S9 Fig). One group of WNH individuals regularly appears at the periphery of the main cluster and does not cluster with any 1KGP populations.

### Regional patterns in the Hispanic subpopulation

**Fig 2:**
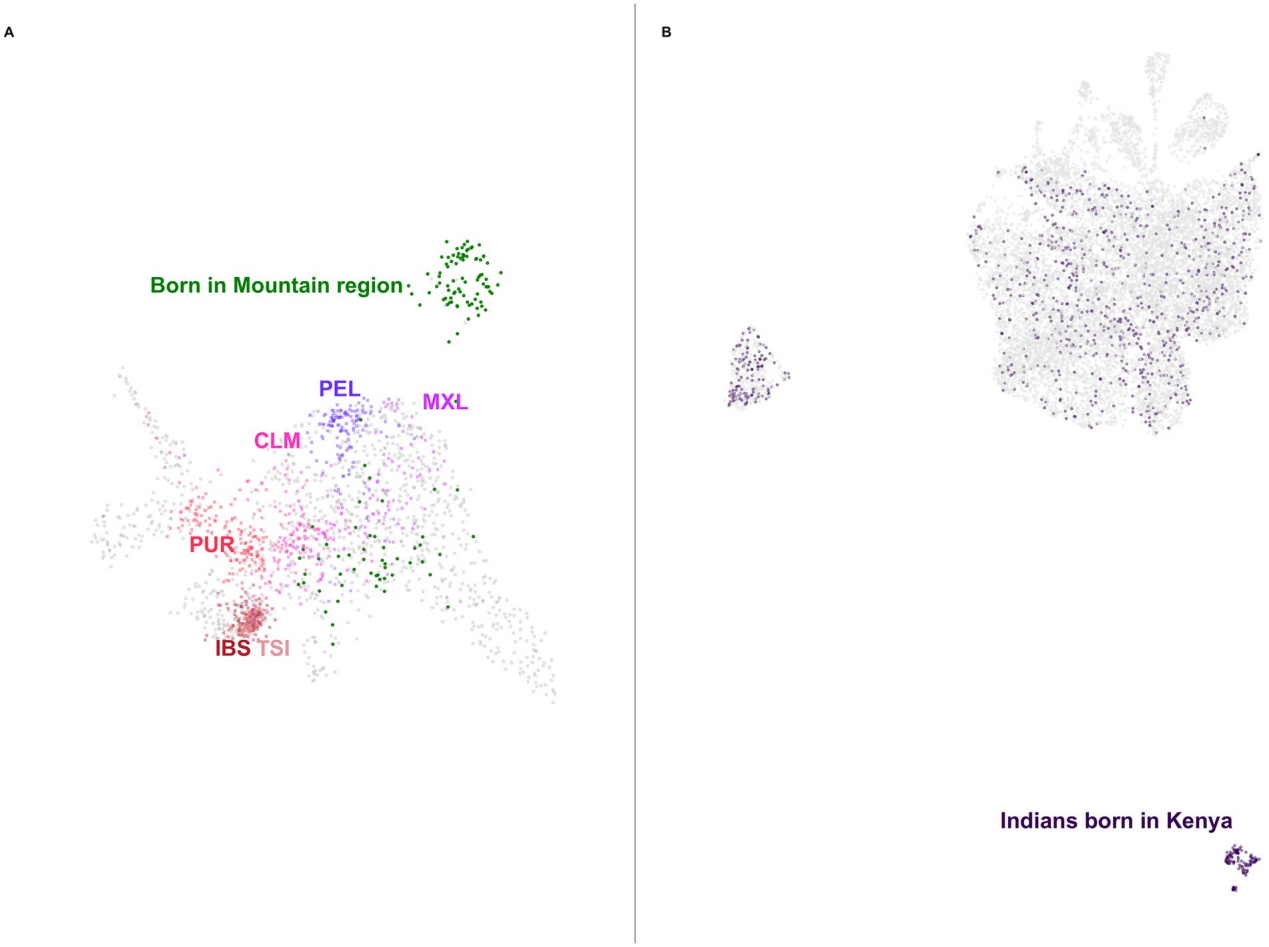
Applying UMAP to subsets of data can reveal deep population structure. (**A**) UMAP on the top 7 principal components of the self-identified Hispanic population of the HRS reveals a cluster. Colouring the points by birthplace shows they were born almost entirely in the Mountain region (in green) of the United States (New Mexico, Arizona, Colorado, Utah, Nevada, Wyoming, Idaho, and Montana). When populations from the 1KGP are projected onto the UMAP embedding they do not map to the cluster. Six 1KGP populations are presented: CLM, Colombian in Medellin, Colombia; IBS, Iberian in Spain; MXL, Mexican in Los Angeles, California; PEL, Peruvian; PUR, Puerto Rican; TSI, Toscani in Italy. S11 and S12 Figs present the same projection of individuals from the HRS coloured by estimated admixture proportions census region of birth, respectively. (**B**) UMAP on the top 8 principal components of the self-identified Asian populations of the UKBB creates clusters. Indian individuals born in Kenya (in purple) form one such cluster. A version coloured by self-identified ethnicity is presented in S13 Fig.

Applying UMAP to self-identified Hispanic individuals in the HRS reveals clear groupings related to birth region in Fig 2A. The highlighted cluster consists almost entirely of individuals born in the Mountain Region of the United States. This cluster is not apparent when looking at a grid of pairwise plots of the first 8 principal components, provided in S10 Fig, as the signal is distributed along PCs 3, 4, and 6. Even though continental admixture patterns do correlate with UMAP position (S11 Fig), these do not explain the Mountain Region cluster. Individuals from 1KGP populations do not appear in the cluster when projected to the UMAP embedding. The cluster possibly comprises the Hispano/Nuevomexicano population of the Southwest US, who have been present in the Mountain Region area long before the more recent immigrants from Latin America, and whose ancestry is expected to reflect both distinct Native ancestry and population-specific drift relative to other Hispanic populations. Such a cluster has been previously identified in AncestryDNA data using network-based clustering on identity-by-descent connections [20]; a recent preprint discusses the Mountain Region Hispanics with a more detailed historical description [21].

### Population structure in the UKBB reflects local and global genetic variation

**Fig 3:**
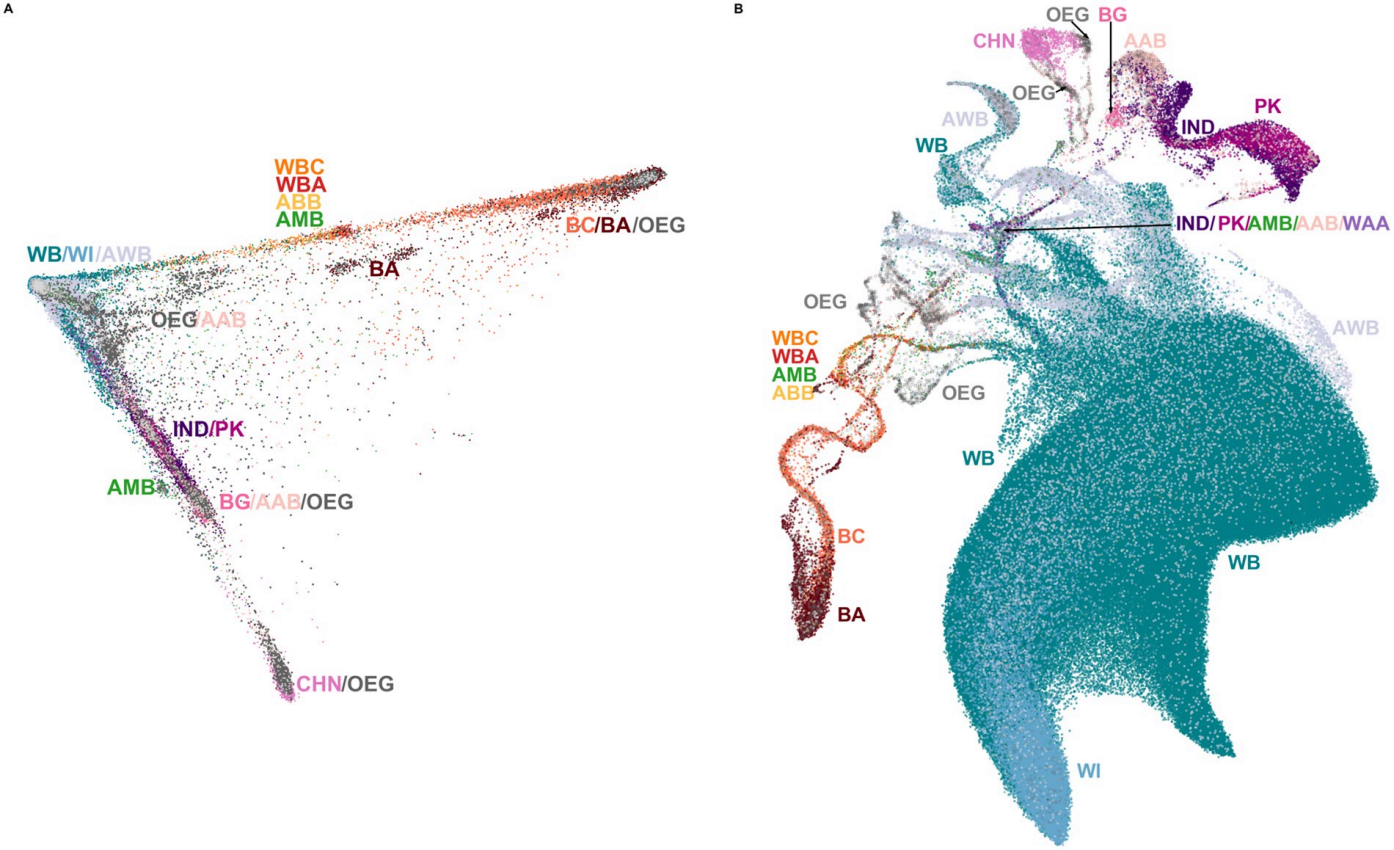
The UKBB coloured by self-reported ethnic background. (**A**) The first two principal components, showing the usual triangle with vertices corresponding to African, Asian, and European ancestries, and intermediate values indicating admixture or lack of relationship to the vertex populations. (**B**) UMAP on the first 10 principal components. The cluster of White British and White Irish individuals is greatly expanded, with the Irish forming a distinct sub cluster mixed with the White British population. South Asian and East Asian individuals form their separate clusters, as do individuals of African or Caribbean backgrounds. Population clusters are connected by “trails” comprised of large proportions of individuals with mixed backgrounds. BA, Black African; BC, Black Caribbean; BG, Bangladeshi; CHN, Chinese; IND, Indian; PK, Pakistani; WB, White British; WI, White Irish; WBC, White and Black Caribbean; WBA, White and Black African; WAA, White and Asian; AAB, Any other Asian Background; ABB, Any other Black Background; AWB, Any other White Background; AMB, Any other Mixed Background; OEG, Other ethnic group.

The UKBB contains data on 488,377 individuals including genotypes, phenotypic measures and self-identified ethnic backgrounds. Fig 3 compares UMAP to PCA applied to the UKBB. As expected, PCA captures major axes of variation emphasizing continental ancestry, whereas UMAP reveals finer structure. UMAP on the top 10 principal components reveals continuous and discrete population structure (Fig 3B): the patchwork of local topologies identifies multiple sub-populations, as well as continuous structure within populations and admixture gradients between populations. The result is a succinct illustration of the complex structure and population relationships in a large and multi-ethnic dataset.

The largest cluster in Fig 3B consists of the White British and Irish populations. The Irish population forms a sub-cluster, but many individuals are also scattered throughout the British-identifying population. Individuals identifying as Black African and Black Caribbean partially overlap, but admixed individuals form distinct trails leading to Asian and European clusters. Chinese individuals form a cluster, within a broader East Asian population; Indian, Pakistani, and Bangladeshi populations form a closely bound cluster as well. The East Asian and South Asian populations each have large clusters of individuals who identify as having an “other Asian background” or belonging to an “other ethnic group”. The patchwork of genetic neighbourhoods is connected by trails of admixed individuals, which converge at a nexus of individuals with a variety of ethnicities. Many claim mixed ancestry, and there are clusters of individuals who belong to an “other ethnic group”. Using data on countries of birth, we identified many finer groups in S14 Fig, and confirmed they appeared in intuitive areas with, e.g., Japanese and Filipino clusters being projected near Chinese clusters.

**Fig 4:**
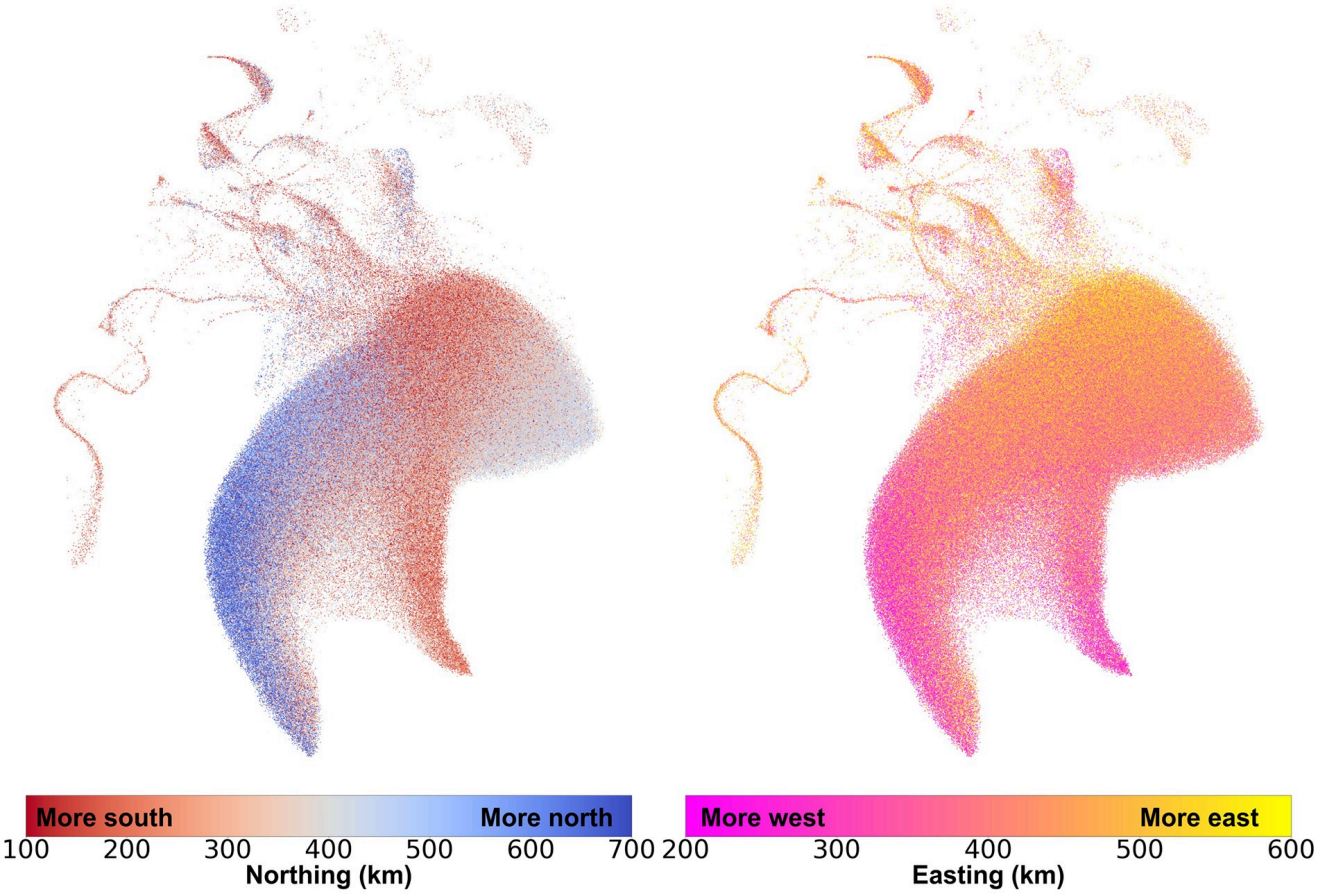
UMAP captures relationships between population structure and geography. Each individual is coloured by their geographical coordinates of residence. Coordinates follow the UKBB’s OSGB1936 geographic grid system and represent distance from the Isles of Scilly, which lie southwest of Great Britain. The left image colours individuals by their north-south (“northing”) coordinates, and the right image colours them by their east-west (“easting”) coordinates. Adding more components creates finer clusters (S17 and S18 Figs). Northing values were truncated between 100km and 700km, and easting values were truncated between 200km and 600km.

Fig 4 presents the UMAP projection from Fig 3B coloured instead by geographical coordinates from the Ordnance Survey National Grid (OSGB1936), with distances defined as a north or east position relative to the Isles of Scilly. Geographic clusters form in the large White British grouping, reflecting the relationship between genetic and geographic distance, as has been observed in Europe and British-wide data [8, 22]. Fig 3B shows that the admixed individuals have UMAP coordinates next to White British individuals residing in the South East of the UK, where London is located (see also S15 Fig, where individuals are coloured by distance from London). This likely reflects the high migration levels to the city and surrounding area: the UMAP projection attempted to preserve both the genetic similarity among admixed individuals and the relatedness with White British individuals in cosmopolitan areas.

The detailed shape of extended clusters is not stable as we vary the number of PCs included, but the patterns mentioned above are preserved. S16, S17 and S18 Figs show UMAP plots using the top 40 PCs from the UKBB.

**Fig 5:**
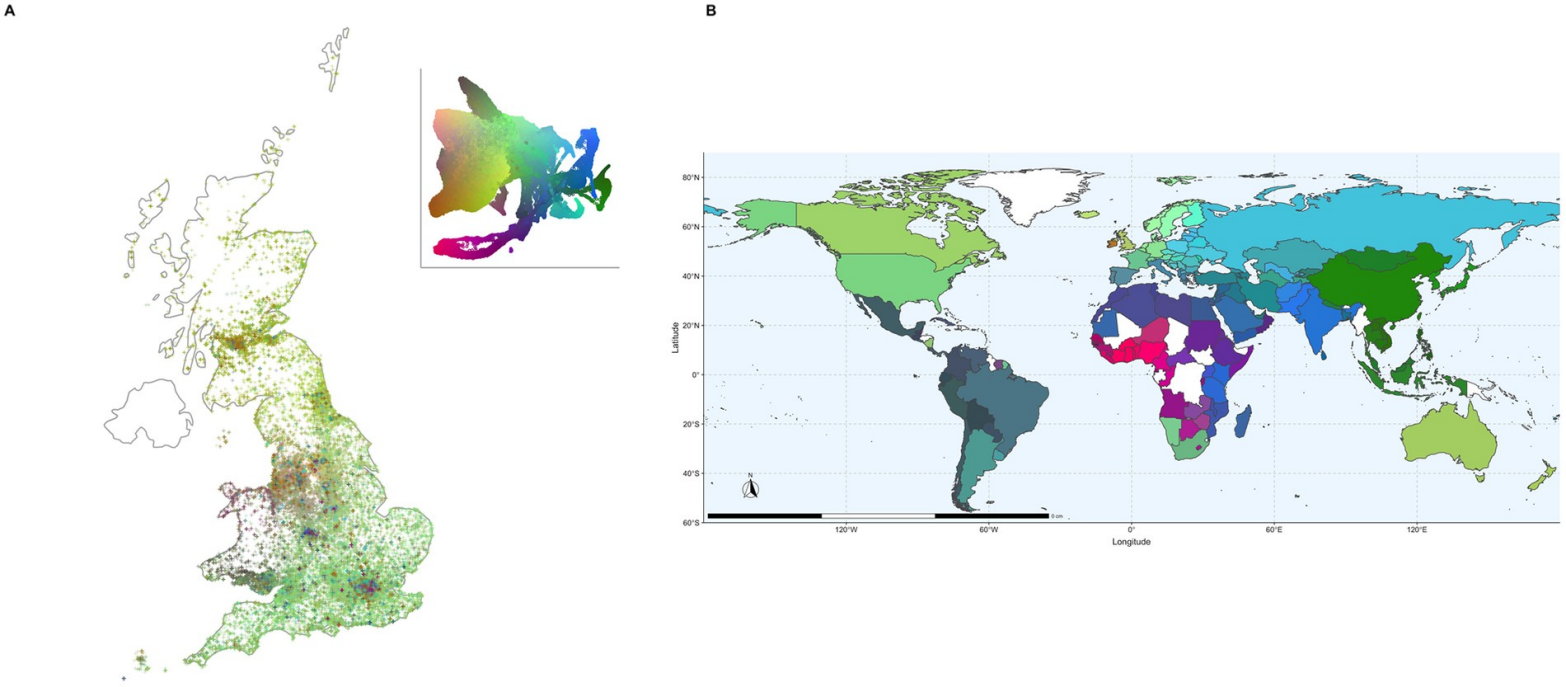
Maps coloured by 3D UMAP projections of the top 20 principal components of the UKBB. Each individual is assigned a 3D RGB vector based on 3D UMAP coordinates (a flattened projection is in the top right of panel A). Individuals who are closer to each other in the projection will be closer in colour in the maps. More details on colouring, as well as randomization of points to protect participant privacy, are available in the materials and methods. (**A**) Each point is an individual placed based on where they live. Patterns in genetic similarity are visible in Scotland, South England, North and South Wales, the East and West Midlands, and major urban centres. (**B**) Geographic distribution of UMAP coordinates. Using the country of birth of individuals in the UKBB, we colour countries by the closeness in 3D UMAP space of those born there. Broad patterns of similarity appear in East Asia, South Asia, North African and the Middle East, West Africa, and South America. Differences between neighbouring countries can reflect both ancient population structure and recent differences in migration history. Evidence of migrations related to colonialism are visible with, e.g., European ancestry in South Africa and South Asian ancestry in Kenya and Tanzania. Because of the large number of White British individuals born abroad, to avoid skewing the colour scale they were not included unless they were born in the UK, Europe, Australia, Canada, or the United States, where UKBB participants already tended to have European ancestry. Zoomed maps of East Asia, the Caribbean, and Europe are available in S19, S20, and S21 Figs, respectively.

As an alternate visualization of geography and genetic diversity, we performed a 3D UMAP projection and converted the UMAP coordinates into RGB values, allowing us to plot individuals on a map of Great Britain, emphasizing both spatial gradients of genetic relatedness and increased diversity in urban centers (Fig 5A). The geographical patterns outside major urban centers are similar to those reported in [22] using the haplotype-based CHROMOPAINTER on British individuals whose grandparents lived nearby. Using data about country of birth, we performed a similar analysis of a world map in Fig 5B, revealing subtle regional variation around the world.

Similarly to UMAP, t-SNE applied to the UKBB data both displays diversity within the “White British” population and identifies clusters among other groups. However, it has three drawbacks: it is much slower, requiring 2.26 hours for its first thousand iterations alone on 10 principal components against UMAP’s 14 minutes; it fails to find a global optimum, which results in a scattering of individuals and groups that are not stable across independent runs; and it does not identify continuity between different continental groups resulting from admixture (S22 Fig).

### Identifying patterns in phenotype variation related to genetic population structure

**Fig 6:**
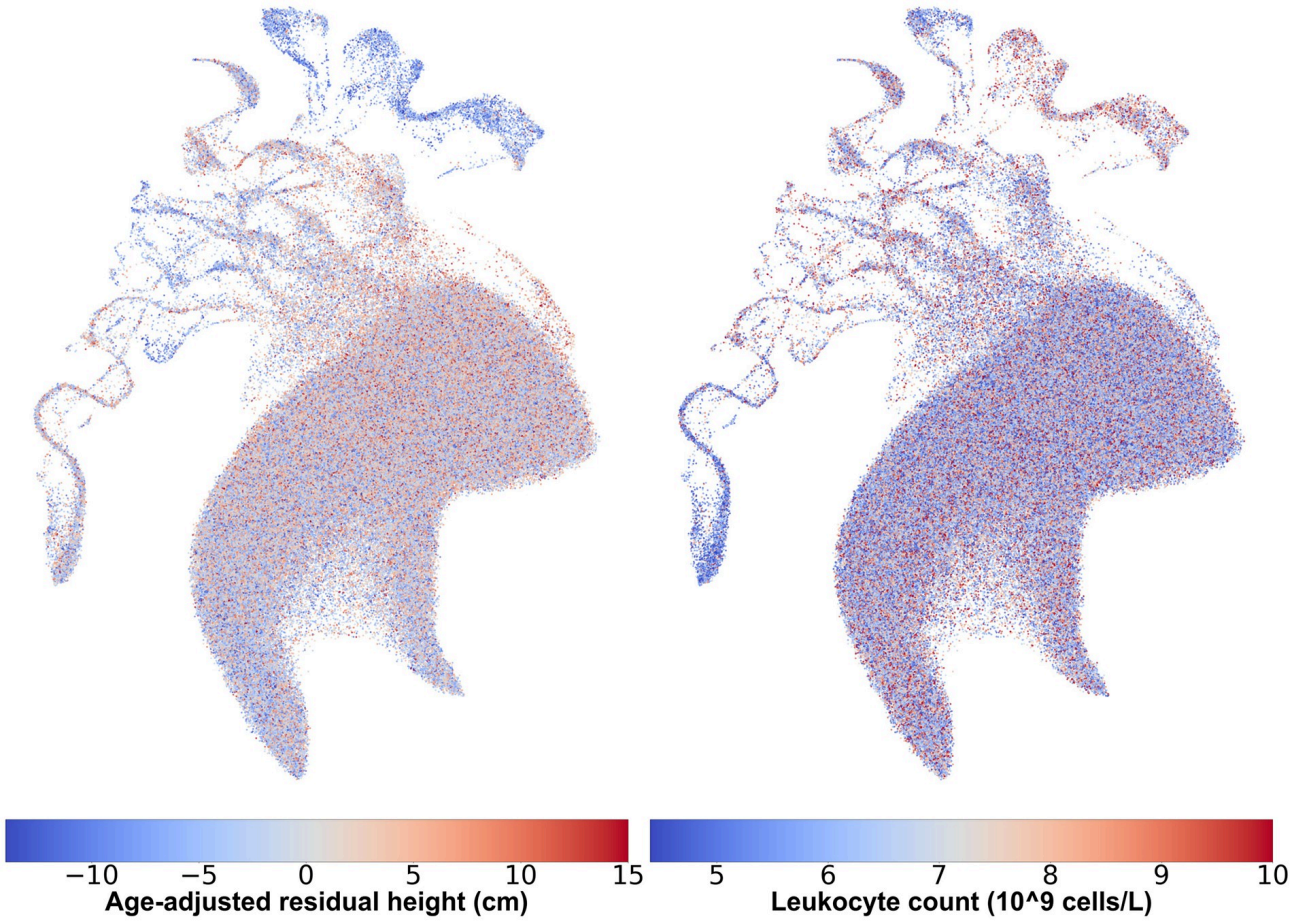
UMAP captures relationships between population structure and phenotype heterogeneity. Females from the UMAP projection in Fig 3B, coloured by age-adjusted difference from mean population height (left) and leukocyte counts (right). Individuals with missing data were excluded. To protect participant privacy, data in these images has been randomized as explained in the materials and methods section.

Covariates such as height and leukocyte count (Fig 6) and autoimmune and asthma-related measures (S23 to S34 Figs) correlate strongly with both discrete and continuous population structure. Several populations in Fig 6, including South Asian, East Asian, African, and others have noticeably lower-than-average heights. More subtle patterns are also visible: the area of the projection in Fig 3B with the cluster of White Irish people appears more blue than the main body of White British individuals. To quantify and statistically test these qualitative observations, we performed an unpaired two sample t-test of self-identified White Irish and White British individuals and found British males taller on average by 0.846cm (p-value 2.10 × 10^−23^) and British females by 0.763cm (p-value 3.65 × 10^−23^) (see S35 and S36 Figs for boxplots). Height differences between Irish and British populations have been previously observed but the direction of the difference is not consistent [23, 24].

In clinical settings, baseline Forced Expiratory Volume in 1 second (FEV1) is determined via equations that include ethnicity or race [25], but studies in several populations have shown that there is considerable variation based on ancestry, even within self-defined ethnicity [26]. S27 and S28 Figs show strong correlations with genetic clustering: certain populations—South Asian, African, and Caribbean—have considerably lower measurements on average (see S37 and S38 Figs for boxplots and p-values).

Notably, there appears to be a juncture in the admixed population, highlighted in S39 Fig, where the distribution of FEV1 changes. This roughly corresponds to the transition from Black African/Caribbean individuals to those who identified having mixed backgrounds. Boxplots and statistical testing suggest that relative to White British populations, FEV1 values are significantly lower for Black African and Black Caribbean populations, but not for White and Black Caribbean and White and Black African populations (S37 and S38 Figs).

S40 Fig further suggests a difference in FEV1 between those who self-identified as Chinese and a nearby cluster enriched in individuals born in Japan; to our knowledge there have not been studies into differences in FEV1 between these populations. To focus on individuals of Asian ancestry (rather than, e.g., individuals born in Japan but who have European ancestry), we first selected the individuals whose UMAP coordinates were near the Chinese cluster. We then focused on individuals born in Japan, Malaysia, and the Philippines as well as the self-identified Chinese population. These four groups are mutually exclusive and are shown in S41 Fig. After adjusting for age, age^2^, height, and sex, an unpaired two-sample t-test shows those born in Japan have a higher mean FEV1 than Chinese individuals by 0.224 (p-value 2.787 × 10^−15^). By sex, there is a difference of 0.213 (p-value 5.4 × 10^−13^) among females and 0.317 (p-value 5.1 × 10^−4^) among males, though there are considerably fewer males in the sample (distributions presented in S42 Fig). For comparison, the adjusted difference between self-identified African and British individuals in the UKBB is 0.762 (p-value 2.2 × 10^−16^).

### Comparing t-SNE and UMAP

Identifying the best dimension reduction technique is challenging, both because the “best” representation depends on context, and because convergence issues may mean that a good theoretical model for dimensional reduction might perform poorly because of challenges in numerical optimization. To assess whether the relatively poor performance of t-SNE could be due to convergence rather than a flawed model, we used UMAP to preprocess the UKBB data and provide a starting point to a standard t-SNE implementation. This led to representations that were objectively better (according to the t-SNE metric) than the default t-SNE implementation (S43 Fig). Yet, these representations were much less detailed than the UMAP embedding provided as a starting point (S44 Fig). Given these results, we recommend UMAP over t-SNE for large and diverse genomic datasets.

## Discussion

Methods such as UMAP and t-SNE focus on preserving local distances to reveal fine-scale structure in populations, and in the process may preserve aspects of global structure as well. In contrast, PCA preserves long range distances but hides finer-scale details. Hierarchical clustering of networks has also successfully detected fine-scale population structure using identity-by-descent similarity by attempting to preserve relations between global networks and smaller local ones (e.g., [20]). We speculate that the addition of weak constraints favouring the preservation of longer distances in UMAP-like approaches has the potential of preserving the desirable local properties while encouraging more intuitive positioning of clusters on a global scale.

UMAP comprehensively illustrates genotypic information at fine scales and within the context of global population structure. It is easy to use and fast: given PCA data and a desktop computer, UMAP can be performed in 15 to 25 minutes on a sample of hundreds of thousands of individuals over tens of dimensions. It excels with larger datasets containing individuals with admixed backgrounds, which present discrete and continuous population structure.

Using UMAP reveals clusters that would have been difficult to identify via pairwise PCA plots or Admixture analysis, such as the geographically restricted cluster within the Hispanic population of the HRS, or the splits within the Gujarati and Punjabi population samples in the 1KGP. More importantly, UMAP helps reveal patterns of covariation between geography, phenotypes and genotypes. Traits such as height showed continuous variation across admixture edges and geographic gradients, as expected from genetically controlled complex traits, and others, such as leukocyte counts or FEV1, showed sharper boundaries and non-linear behavior consistent with the existence of strong regional environmental influences.

We found that pre-processing the data with PCA allowed for time savings, but identifying an optimal number of PCs to use is challenging. Groupings on ethnicity formed slowly as PCs were added until reaching a stable number around 10 to 15 PCs. Geographical patterns in the UKBB continued to appear even up to 40 components, as visible in S17 and S18 Figs.

### Caveats

In contrast to PCA, UMAP has more adjustable parameters. Changing the PC cutoff, minimum distance, and number of neighbours can change characteristics of the visualizations. Using a minimal number of neighbours (e.g. 5 rather than the default 15) can result in the formation of disjoint clusters comprised of related family members (S5 Fig), and using a low minimum distance (e.g. 0.001 rather than the default 0.1) can result in clusters becoming more compact, losing visual detail. We used a minimum distance of 0.5 and 15 neighbours; however, default suggested parameters in UMAP generally perform well across datasets.

In the absence of clear theoretical rationales, we suggest to use as many PCs as are available and computationally feasible, even though we sometimes found that a lower number of PCs led to a simpler shape that facilitated discussion (e.g. Fig 3B). Overall, we recommend reporting on a range of parameter values and following up on observations with statistical testing.

Like most non-linear methods, UMAP lacks direct interpretability. It emphasizes local distances over global distances; while points that are very close in UMAP space are likely close in the original data, points that are distant in UMAP space are not necessarily very different in the original data. Disconnected clusters may also change their positions relative to other clusters over the course of multiple projections, as in S2 Fig. For these reasons, UMAP coordinates should not be used as GWAS covariates or for quantifying distances between populations. UMAP is sensitive to sample sizes and spends more visual space on populations with larger sample sizes. This is useful to identify significant patterns in a cohort, but it makes comparing visualization across cohorts difficult and may appear to exaggerate the genetic variation within the most sampled populations, such as the White British population in the UKBB. We did not assign meaning to wiggles in UMAP figures, which occurred consistently in the UKBB but may be an artefact of the dimensional reduction strategy rather than a meaningful feature of the data. Hand-waving interpretations of pretty plots have a history of getting population geneticists in trouble (as pointed out, e.g., in [27]): visualization is not a replacement for statistical testing.

With these caveats in mind, a priori data visualization plays a central role in quality control, hypothesis generation, and confounder identification for a wide range of genomic applications. Non-linear approaches, despite their limitations, become increasingly useful as the size of datasets increases. UMAP, in particular, reveals a wide range of features that would not be apparent using linear maps. Given its ease of use, broad applicability, and low computational cost, we propose that UMAP should become a default companion to PCA and other population structure visualization and inference methods in large genomic cohorts.

## Materials and methods

We used genotype data from 12,454 individuals from the Health and Retirement Study (HRS), genotyped on the Illumina Human Omni 2.5M platform [16]. Principal components were computed in PLINK v1.90b5.2 64-bit [28] using variants with a minor allele frequency greater than 0.05, Hardy-Weinberg p-value of more than 1 × 10^−6^, and genotype missing rate of less than 0.1, and sample with genotype missing rate of less than 0.1. We used the principal components of genotype data from 488,377 individuals in the UK BioBank (UKBB) as computed by the cohort [17]. We used genotype data from 3,450 individuals from the 1KGP project using Affy 6.0 genotyping [12].

The HRS contains genotype data of 12,454 American individuals across all 50 states who have provided racial identity (10,434 White, 1,652 Black, 368 Other) as well as whether they identify as Hispanic (1,203 total) and, if so, whether they identify as Mexican-American (705 total) [16]. We crossed these three variables to form a composite self-reported ethnicity resulting in 10 categories (e.g. White Hispanic Mexican-American), and considered birth regions based on the 10 census regions and divisions used by the US Census Bureau. Admixture proportions for each individual were estimated in [29] by assuming ancestral African, Asian, and European populations using RFMIX [30]. We have scaled each of the three proportions to values between 0 and 255 (with 100% corresponding to 255), to colour individual points by their estimated admixture represented by RGB where red, green, and blue respectively correspond to African, European, and Asian/Native American ancestry. To project 1KGP data on HRS embeddings, as in Fig 2A and S9 Fig, we created the PC axes and UMAP embedding for the HRS data and then projected the 1KGP data onto them.

The UKBB provides genotype data on 488,377 individuals along with self-identified ethnic background in a hierarchical tree-structured dictionary. Participants provided ethnic background on two occasions. We used the initial ethnicity after finding minimal differences between the two. The dataset is majority White (88.3% British, 2.6% Irish, 3.4% other), with large populations identifying as Black (1.6% either African, Caribbean, or other), Asian (1.9% either Indian, Pakistani, Bangladeshi, or other), Chinese (0.3%), an other ethnic group (0.8%), mixed ethnicity (0.6%), or an unavailable response (0.5%).

Scripts for all tests and plotting functions can be found on https://github.com/diazale/gt-dimred with a command line script for UMAP available at https://github.com/diazale/gt-dimred/scripts/general_umap_script.py. A demo version using freely available 1KGP data is available at https://github.com/diazale/1KGP_dimred. PCA and standard t-SNE were done with Scikit-learn [31]. UMAP was performed using a Python implementation [14]. Statistical testing was done in SciPy [32], StatsModels [33], and R [34]. Visualizations were created with Matplotlib [35] and ggplot2 [36], and maps were made with Natural Earth.

Both UMAP and t-SNE feature a number of adjustable parameters. Among the parameters that we varied, the number of PCs used in pre-processing of the data has the largest effect for both methods (see S1 and S2 Figs). With UMAP, there are other parameters, such as the learning rate and the distance metric; these were left to the default values.

We tested different choices for perplexity in t-SNE. The default value of 30 provided comparable performance to other parameter choices. Similarly, we tested different parameter choices for UMAP, with the clearest results generated by specifying 15 nearest neighbours (the default value) and a “minimum distance” between points in low dimensions of 0.5. UMAP developers described “sensible” values for nearest neighbours as between 5 and 50 and minimum distance between 0.5 and 0.001. Tuning these parameters will not change qualitative results much but may make patterns easier to identify. Increasing the number of neighbours will increase the computational load, and a smaller minimum distance can break the connectivity between clusters, though the same individuals will continue to group together.

UMAP and t-SNE projections were carried out on an iMac with a 3.5GhZ Intel Core i7 processor, 32 GB 1600 MHz DDR3 of RAM, and an NVIDIA GeForce GTX 775M 2048 MB graphics card.

Colours for maps in Fig 5A and 5B, S19, S20, and S21 Figs were determined by projecting data to 3D and using each 3D coordinate as an RGB coordinate. For the world map, countries were determined using the country of birth variable, with a country’s colour being determined by the mean *x*, *y*, and *z* values of all individuals born in that country. Because many self-identified White British individuals were born abroad, including them everywhere would skew the colour scheme; they were included only if they were born in the UK, Europe, Australia, Canada, or the United States. This approach to colouring is sensitive to sample sizes as UMAP will give more space to larger populations.

To reduce the potential risks for re-identification from results in this publication, data has been randomly permuted so that the population characteristics are preserved but individual-level data is not presented directly in the figures. We rounded each attribute to an attribute-specific number of bins, and then permuted the data in the following way: For each point (i.e. each individual) in UMAP visualizations, and each attribute, we identified the 9 nearest neighbouring points, and copied the attribute from a randomly selected neighbour (thus allowing for the possibility of one value being printed twice). Because this process is done independently for each visualization, a given point shown on the figure will copy values from different randomly selected individuals. Projections coloured by participants’ spatial coordinates have random noise added (normally distributed about 0 with a standard deviation of 50km) before binning to the nearest 50km. Projections coloured by participants’ distance from London have random noise added (normally distributed about 0 with a standard deviation of 5km) before binning to the nearest km. For each point in Fig 5A we identified the nearest 50 neighbouring individuals and copied the colour value from a randomly selected neighbour.

## Supporting information

S1 Fig Montage of t-SNE and UMAP on up to 9 PCs of 1KGP data. UMAP (left two columns) and t-SNE (right two columns) applied to the top principal components of the 1KGP labelled by the number of components used. Adding more components results in progressively finer population clusters using both methods.
(PDF)

S2 Fig Montage of t-SNE and UMAP on 10 to 50 PCs of 1KGP data. UMAP (left two columns) and t-SNE (right two columns) applied to the top principal components of the 1KGP labelled by the number of components used. Results are similar until approximately 11 components, where t-SNE breaks apart clusters of South Asian (in green) and Central and South American populations (in pink) while UMAP preserves them. At approximately 30 components populations begin to drift together with UMAP and disperse with t-SNE.
(PDF)

S3 Fig Montage of UMAP on progressively more PCs of 1KGP data. UMAP applied to the first few hundred principal components of the 1KGP data with the amount of variance explained in parentheses. As more components are added, the figure begins to resemble that of UMAP carried out on the full genotype dataset.
(JPEG)

S4 Fig UMAP on PCs 100 to 3350 of 1KGP data. UMAP applied the last 3350 principal components of the 1KGP, which explain 78.7% of the variation. The colour scheme is the same as in Fig 1.
(JPEG)

S5 Fig Number of neighbours and families forming disjoint clusters. UMAP applied to the first 15 principal components of the 1KGP, with the number of neighbours set to 5 (top) and 15 (bottom). Six members of one Southern Han Chinese family are highlighted: HG00656 (grandfather), HG00657 (grandmother), HG00658 (uncle, mother’s brother), HG00701 (mother), HG00702 (father), HG00703 (child). When using UMAP with five neighbours, the father (in blue) is projected to the cluster of the Southern Han Chinese population while the rest of the family members (in red) form their own disjoint cluster. Using 15 neighbours, the family still clusters together, but as part of the Southern Han Chinese population rather than a separate cluster.
(PNG)

S6 Fig UMAP on HRS data coloured by ethnicity. UMAP applied to the first 10 principal components of HRS data. Points coloured by self-identified race, Hispanic status, and Mexican-American status. The cluster on the left is mostly people who identify as neither Black nor White and were born outside the contiguous United States or in the Pacific census region. Clustering with the 1KGP data places them with Asian-identified populations. BNH, Black (not Hispanic); BHO, Black (Hispanic, Other); WNH, White (not Hispanic); WHM, White (Hispanic, Mexican-American); WHO, White Hispanic (Other); ONH, Other (not Hispanic); OHM, Other (Hispanic, Mexican-American); OHO, Other (Hispanic, Other).
(PDF)

S7 Fig UMAP on HRS data coloured by admixture. UMAP on the first 10 principal components of HRS data. colouring individuals by estimated admixture from three ancestral populations reveals considerable diversity in the Hispanic population. This projection coloured by self-identified race and Hispanic status is presented in S6 Fig. Admixture proportions for each individual were estimated in (Baharian 2016) by assuming ancestral African, Asian, and European populations using RFMIX. We have scaled each of the three proportions to values between 0 and 255 (with 100% corresponding to 255), to colour individual points by their estimated admixture represented by RGB where red, green, and blue respectively correspond to African, European, and Asian/Native American ancestry. An alternate colouring is provided in S63 Fig.
(JPEG)

S8 Fig UMAP on HRS data coloured by birth region. UMAP on the top 10 principal components of the HRS dataset, coloured by Census Bureau birth region. Each colour represents one of the 10 birth regions. There is no obvious pattern in the clusters of majority “White Not Hispanic” individuals.
(JPEG)

S9 Fig UMAP on HRS data with 1KGP data overlaid. UMAP on the top 10 principal components of the HRS data, with 1KGP data projected onto the embedding. Individuals from the HRS are grey. British (GBR) and other European (CEU) individuals are scattered throughout the “White Not Hispanic” clusters. Finns (FIN) form clear groupings. Spanish (IBS) and Italian (TSI) individuals cluster near the Hispanic grouping. There are sub-groups in the Hispanic cluster formed of Puerto Ricans (PUR), Colombians (CLM), Mexicans (MXL), and Peruvians (PEL). Populations with African ancestry (AFR) appear with Black individuals. East Asian (EAS) populations comprising Chinese, Kinh, and Japanese individuals cluster together with what appears in S7 Fig as a population of mostly Asian ancestry. South Asian (SAS) populations with Indian, Pakistani, and Sri Lankan ancestry cluster in a separate area. One “White Not Hispanic” cluster at the bottom does not cluster with any 1KGP populations.
(PDF)

S10 Fig Pairwise plots of PCs of Hispanic HRS data. Pairwise plots of the first 8 principal components of the Hispanic subset of the HRS. Those born in the Mountain region are coloured green.
(PDF)

S11 Fig UMAP on Hispanic HRS data coloured by admixture. UMAP of the first 7 principal components of the Hispanic population of the HRS, coloured by estimated admixture proportions. Admixture proportions for each individual were estimated in (Baharian, 2016) by assuming ancestral African, Asian, and European populations using RFMIX. We have scaled each of the three proportions to values between 0 and 255 (with 100% corresponding to 255), to colour individual points by their estimated admixture represented by RGB where red, green, and blue respectively correspond to African, European, and Asian/Native American ancestry. An alternate colouring is provided in S64 Fig.
(JPEG)

S12 Fig UMAP on Hispanic HRS data coloured by birth region. UMAP of the first 7 principal components of the Hispanic population of the HRS, coloured region of birth.
(JPEG)

S13 Fig UMAP on Asian UKBB data coloured by self-identified ethnicity. UMAP of the first 8 principal components of the Asian population in the UKBB coloured by self-identified ethnicity. This is an alternate colouring of Fig 2B.
(JPEG)

S14 Fig UMAP on UKBB data with some countries of birth identified. Using country of birth data, some of the larger unidentified groups from Fig 3B were identified as being born mostly in Japan, the Philippines, North Africa, the Middle East, and Central and South America. The large cluster of “Any other Asian Background” were mostly born in Sri Lanka.
(JPEG)

S15 Fig UMAP on UKBB data coloured by distance from London. UMAP on UKBB data, coloured by distance from London, with red representing those living closer to London and blue representing those living farther from London. A 200km radius extends roughly to Cardiff, and a 100km radius extends roughly to cities such as Leicester and Bath, and contains cities such as Oxford, Cambridge, and Peterborough. Data has been randomized as explained in the materials and methods section.
(JPEG)

S16 Fig Montage of UMAP on top 40 PCs of UKBB data coloured by ethnicity. UMAP on UKBB data, coloured by self-identified ethnic background. Images are labelled by the number of components included.
(JPEG)

S17 Fig Montage of UMAP on top 40 PCs of UKBB data coloured by northing. UMAP on UKBB data, coloured by northing values, with more blue representing more northern coordinates and more red representing more southern coordinates. Images are labelled by the number of components included. Data has been randomized as explained in the materials and methods section.
(JPEG)

S18 Fig Montage of UMAP on top 40 PCs of UKBB data coloured by easting. UMAP on UKBB data, coloured by easting values, with more yellow representing more eastern coordinates and more pink representing more western coordinates. Images are labelled by the number of components included. Data has been randomized as explained in the materials and methods section.
(JPEG)

S19 Fig Map of Asia coloured by 3D UMAP coordinates of UKBB data. Fig 5b, zoomed in on Asia. Geographic distribution of UMAP coordinates. Using the country of birth of individuals in the UKBB, we colour countries by the closeness in 3D UMAP space of those born there. Broad patterns of similarity appear in East Asia, South Asia, North African and the Middle East, West Africa, and South America. Differences between neighbouring countries can reflect both ancient population structure and recent differences in migration history. Evidence of migrations related to colonialism are visible with, e.g., European ancestry in South Africa and South Asian ancestry in Kenya and Tanzania. Because of the large number of White British individuals born abroad, to avoid skewing the colour scale they were not included unless they were born in the UK, Europe, Australia, Canada, or the United States, where UKBB participants already tended to have European ancestry.
(JPG)

S20 Fig Map of Caribbean coloured by 3D UMAP coordinates of UKBB data. Fig 5b, zoomed in on the Caribbean. Geographic distribution of UMAP coordinates. Using the country of birth of individuals in the UKBB, we colour countries by the closeness in 3D UMAP space of those born there. Broad patterns of similarity appear in East Asia, South Asia, North African and the Middle East, West Africa, and South America. Differences between neighbouring countries can reflect both ancient population structure and recent differences in migration history. Evidence of migrations related to colonialism are visible with, e.g., European ancestry in South Africa and South Asian ancestry in Kenya and Tanzania. Because of the large number of White British individuals born abroad, to avoid skewing the colour scale they were not included unless they were born in the UK, Europe, Australia, Canada, or the United States, where UKBB participants already tended to have European ancestry.
(JPG)

S21 Fig Map of Europe coloured by 3D UMAP coordinates of UKBB data. Fig 5b, zoomed in on Europe. Geographic distribution of UMAP coordinates. Using the country of birth of individuals in the UKBB, we colour countries by the closeness in 3D UMAP space of those born there. Broad patterns of similarity appear in East Asia, South Asia, North African and the Middle East, West Africa, and South America. Differences between neighbouring countries can reflect both ancient population structure and recent differences in migration history. Evidence of migrations related to colonialism are visible with, e.g., European ancestry in South Africa and South Asian ancestry in Kenya and Tanzania. Because of the large number of White British individuals born abroad, to avoid skewing the colour scale they were not included unless they were born in the UK, Europe, Australia, Canada, or the United States, where UKBB participants already tended to have European ancestry.
(JPG)

S22 Fig t-SNE on UKBB data coloured by self-identified ethinicity. t-SNE applied to the top 10 principal components of the UKBB, coloured by ethnic background. The unbalanced populations resulted in many individuals and populations being orphaned along the periphery of the main cluster.
(PDF)

S23 Fig UMAP on UKBB data coloured by basophil count (female). UMAP on the top 10 principal components of the UKBB coloured by basophil count (female). Data has been randomized as explained in the materials and methods section.
(PDF)

S24 Fig UMAP on UKBB data coloured by basophil count (male). UMAP on the top 10 principal components of the UKBB coloured by basophil count (male). Data has been randomized as explained in the materials and methods section.
(PDF)

S25 Fig UMAP on UKBB data coloured by eosinphil count (female). UMAP on the top 10 principal components of the UKBB coloured by eosinophil count (female). Data has been randomized as explained in the materials and methods section.
(PDF)

S26 Fig UMAP on UKBB data coloured by eosinphil count (male). UMAP on the top 10 principal components of the UKBB coloured by eosinophil count (male). Data has been randomized as explained in the materials and methods section.
(PDF)

S27 Fig UMAP on UKBB data coloured by FEV1 (female). UMAP on the top 10 principal components of the UKBB coloured by FEV1 (female). Data has been randomized as explained in the materials and methods section.
(PDF)

S28 Fig UMAP on UKBB data coloured by FEV1 (male). UMAP on the top 10 principal components of the UKBB coloured by FEV1 (male). Data has been randomized as explained in the materials and methods section.
(PDF)

S29 Fig UMAP on UKBB data coloured by height (female). UMAP on the top 10 principal components of the UKBB coloured by height (female). Data has been randomized as explained in the materials and methods section.
(PDF)

S30 Fig UMAP on UKBB data coloured by height (male). UMAP on the top 10 principal components of the UKBB coloured by height (male). Data has been randomized as explained in the materials and methods section.
(PDF)

S31 Fig UMAP on UKBB data coloured by leukocyte count (female). UMAP on the top 10 principal components of the UKBB coloured by leukocyte count (female). Data has been randomized as explained in the materials and methods section.
(PDF)

S32 Fig UMAP on UKBB data coloured by leukocyte count (male). UMAP on the top 10 principal components of the UKBB coloured by leukocyte count (male). Data has been randomized as explained in the materials and methods section.
(PDF)

S33 Fig UMAP on UKBB data coloured by neutrophil count (female). UMAP on the top 10 principal components of the UKBB coloured by neutrophil count (female). Data has been randomized as explained in the materials and methods section.
(PDF)

S34 Fig UMAP on UKBB data coloured by neutrophil count (male). UMAP on the top 10 principal components of the UKBB coloured by neutrophil count (male). Data has been randomized as explained in the materials and methods section.
(PDF)

S35 Fig Box plots of height in the UKBB by self-identified ethnicity (female). Height by sex and ethnic group, annotated with p-values. Asterisks indicate significant difference from the White British group with a Bonferroni correction for 12 groups.
(PDF)

S36 Fig Box plots of height in the UKBB by self-identified ethnicity (male). Height by sex and ethnic group, annotated with p-values. Asterisks indicate significant difference from the White British group with a Bonferroni correction for 12 groups.
(PDF)

S37 Fig Box plots of FEV1 in the UKBB by self-identified ethnicity (female). FEV1 by sex and ethnic group, annotated with p-values. Asterisks indicate significant difference from the White British group with a Bonferroni correction for 12 groups.
(PDF)

S38 Fig Box plots of FEV1 in the UKBB by self-identified ethnicity (male). FEV1 by sex and ethnic group, annotated with p-values. Asterisks indicate significant difference from the White British group with a Bonferroni correction for 12 groups.
(PDF)

S39 Fig Subset (left) of UKBB UMAP projection coloured by height, FEV1, and self-identified ethnicity. Individuals of Black African, Black Caribbean, and mixed backgrounds (primarily White and Black Caribbean/African) coloured by self-identified ethnic background (left, from Fig 3B), FEV1 (middle), and age-adjusted height (right). An arrow points to an area where the FEV1 distribution appears to change, corresponding to where the clusters contain more people with self-identified mixed backgrounds.
(PDF)

S40 Fig Subset (top) of UKBB UMAP projection coloured by height, FEV1, and self-identified ethnicity. Zoomed in section of Fig 3B, focused on individuals with Chinese (CHI), White British (GBR), any other white background, or any other ethnic group (OEG) coloured by ethnicity (left), FEV1 (middle), and age-adjusted height (right). The OEG cluster next to the Chinese cluster appears redder on the middle panel, suggesting higher levels of FEV1.
(PNG)

S41 Fig East Asian individuals from UKBB UMAP projection selected for FEV1 investigation. Individuals from the zoomed in section in S40 Fig used in statistical testing, coloured the same as in S42 Fig. Brown, blue, and green represent those born in the Philippines, Malaysia, and Japan; pink represents those who self-identify as Chinese. The Chinese individuals were those who self-identified their ethnic background as Chinese, and the remaining populations were determined based on country of birth; the categorizations are mutually exclusive.
(PNG)

S42 Fig Ridge plots of East Asian individuals from UKBB UMAP projection selected for FEV1 investigation. Plots of the distributions of residual FEV1 by sex for East Asian populations, after adjusting for height, age, age^2^, and sex through linear regression. Individuals were limited to those in the “Chinese/Other Ethnic Group” cluster from S40 Fig. The Chinese individuals were those who self-identified their ethnic background as Chinese, and the remaining populations were determined based on country of birth; the categorizations are mutually exclusive. Asterisks indicate significant difference from the Japanese population, using Welch’s unpaired t-test with a Bonferroni correction for 3 groups. The dashed lines are the means of the distributions, and Japanese populations have consistently higher means.
(PNG)

S43 Fig Comparison of t-SNE error by initialization on UKBB data. Comparing the error terms of standard t-SNE versus t-SNE initialized with a UMAP embedding and no early exaggeration. Done on the UKBB dataset with 20000 iterations. The UMAP-initialized graph has been shifted by 230 iterations to approximate the 230 epochs UMAP uses for large datasets (*n* > 10, 000).
(JPEG)

S44 Fig Comparing visualizations of t-SNE and UMAP of UKBB data by initialization. Comparing the visualizations of UMAP, standard t-SNE, and t-SNE initialized with a UMAP projection, on the top 10 principal components of the UKBB. t-SNE used 20000 iterations.
(JPEG)

S45 Fig PCs 1 and 2 of the UKBB coloured by height (female). Principal components 1 and 2 from the UKBB, coloured by age-adjusted residual height (female). Data has been randomized as explained in the materials and methods section.
(JPEG)

S46 Fig PCs 1 and 2 of the UKBB coloured by FEV1 (female). Principal components 1 and 2 from the UKBB, coloured by FEV1 (female). Data has been randomized as explained in the materials and methods section.
(JPEG)

S47 Fig t-SNE projection of UKBB data coloured by height (female). t-SNE on the first 10 principal components from the UKBB, coloured by age-adjusted residual height (female). Data has been randomized as explained in the materials and methods section.
(JPEG)

S48 Fig t-SNE projection of UKBB data coloured by FEV1 (female). t-SNE on the first 10 principal components from the UKBB, coloured by FEV1 (female). Data has been randomized as explained in the materials and methods section.
(JPEG)

S49 Fig Zoomed in views of UMAP projection of UKBB data, coloured by self-identified ethnicity. Zoomed in areas of Fig 3B. Sections (i) and (ii) respectively focus on the African and Asian superpopulations, and section (iii) focuses on an area with individuals from many ethnic backgrounds. Noticeable clusters of unidentified ethnic backgrounds appear and are labelled “OEG” (“Other Ethnic Group”).
(PDF)

S50 Fig Comparing visualizations of t-SNE and UMAP of 1KGP data by initializaiton. Comparing the visualizations of UMAP, standard t-SNE, and t-SNE initialized with a UMAP projection, on the top 10 principal components of the 1KGP. t-SNE used 5000 iterations. Initializing t-SNE with UMAP breaks the continuous structure of the projection and instead forms many small clusters.
(JPEG)

S51 Fig Comparing visualizations of t-SNE and UMAP of HRS data by initialization. Comparing the visualizations of UMAP, standard t-SNE, and t-SNE initialized with a UMAP projection, on the top 10 principal components of the HRS. t-SNE used 5000 iterations.
(JPEG)

S52 Fig Comparison of t-SNE error by initialization on 1KGP data. Comparing the error terms of standard t-SNE versus t-SNE initialized with a UMAP embedding and no early exaggeration. Done on the 1KGP dataset with 5000 iterations. The UMAP-initialized graph has been shifted by 600 iterations to approximate the 600 epochs UMAP uses for small datasets (*n* <= 10, 000).
(JPEG)

S53 Fig Comparison of t-SNE error by initialization on HRS data. Comparing the error terms of standard t-SNE versus t-SNE initialized with a UMAP embedding and no early exaggeration. Done on the HRS dataset with 5000 iterations. The UMAP-initialized graph has been shifted by 230 iterations to approximate the 230 epochs UMAP uses for large datasets (*n* > 10,000).
(JPEG)

S54 Fig Box plots of basophil count in the UKBB by self-identified ethnicity (female). Basophil counts by sex and ethnic group, annotated with p-values. Asterisks indicate significant difference from the White British group with a Bonferroni correction for 12 groups.
(PDF)

S55 Fig Box plots of basophil count in the UKBB by self-identified ethnicity (male). Basophil counts by sex and ethnic group, annotated with p-values. Asterisks indicate significant difference from the White British group with a Bonferroni correction for 12 groups.
(PDF)

S56 Fig Box plots of eosinophil count in the UKBB by self-identified ethnicity (female). Eeosinophil counts by sex and ethnic group, annotated with p-values. Asterisks indicate significant difference from the White British group with a Bonferroni correction for 12 groups.
(PDF)

S57 Fig Box plots of eosinophil count in the UKBB by self-identified ethnicity (male). Eosinophil counts by sex and ethnic group, annotated with p-values. Asterisks indicate significant difference from the White British group with a Bonferroni correction for 12 groups.
(PDF)

S58 Fig Box plots of leukocyte count in the UKBB by self-identified ethnicity (female). Leukocyte counts by sex and ethnic group, annotated with p-values. Asterisks indicate significant difference from the White British group with a Bonferroni correction for 12 groups.
(PDF)

S59 Fig Box plots of leukocyte count in the UKBB by self-identified ethnicity (male). Leukocyte counts by sex and ethnic group, annotated with p-values. Asterisks indicate significant difference from the White British group with a Bonferroni correction for 12 groups.
(PDF)

S60 Fig Box plots of neutrophil count in the UKBB by self-identified ethnicity (female). Neutrophil counts by sex and ethnic group, annotated with p-values. Asterisks indicate significant difference from the White British group with a Bonferroni correction for 12 groups.
(PDF)

S61 Fig Box plots of neutrophil count in the UKBB by self-identified ethnicity (male). Neutrophil counts by sex and ethnic group, annotated with p-values. Asterisks indicate significant difference from the White British group with a Bonferroni correction for 12 groups.
(PDF)

S62 Fig UMAP projection of combined HRS and 1KGP data. UMAP projection of the top 10 principal components of the combined HRS and 1KGP datasets. One cluster (in the box) does not group with any of the 1KGP populations. A cluster of Finnish (FIN) individuals consistently appears in the “White Not Hispanic” (WNH) group. Groups of Central and South American populations from the 1KGP (CLM, Colombian; MXL, Mexican; PEL, Peruvian; PUR, Puerto Rican) form nearby or within the HRS Hispanic cluster (HIS). Iberian individuals (IBS) cluster near the Hispanic population. Toscani individuals (TSI) form some small clusters and sometimes appear near the Iberian and Hispanic populations. Individuals with British/Scottish (GBR) or Northern/Western European ancestry (CEU) are scattered throughout the WNH clusters. Individuals with African ancestry from the 1KGP group with Black Americans from the HRS (AFR). Similar population groupings occur with South Asian (SAS) and East Asian (EAS) individuals.
(PDF)

S63 Fig Alternate colouring of S7 Fig. An alternate colouring of S7 Fig. Here red, green, and blue correspond to African, Asian/Native American, and European ancestry, respectively.
(JPEG)

S64 Fig Alternate colouring of S11 Fig. An alternate colouring of S11 Fig. Here red, green, and blue correspond to African, Asian/Native American, and European ancestry, respectively.
(JPEG)

S65 Fig Admixture plot of Hispanic individuals in the HRS. Admixture plot of Hispanic individuals in the HRS. Individuals born in the Mountain census region fall between the white lines (indices 48 to 184).
(PDF)

S1 Table Variance explained by the PCs of the 1KGP. Variance explained in the 1KGP data by the number of principal components used.
(PDF)
