# Supplementary figures and images for: UMAP reveals cryptic population structure and phenotype heterogeneity in large genomic cohorts
Source: PLoS Genet. 2019 Nov 1;15(11):e1008432. doi: 10.1371/journal.pgen.1008432 (PMC6853336; doi:10.1371/journal.pgen.1008432)

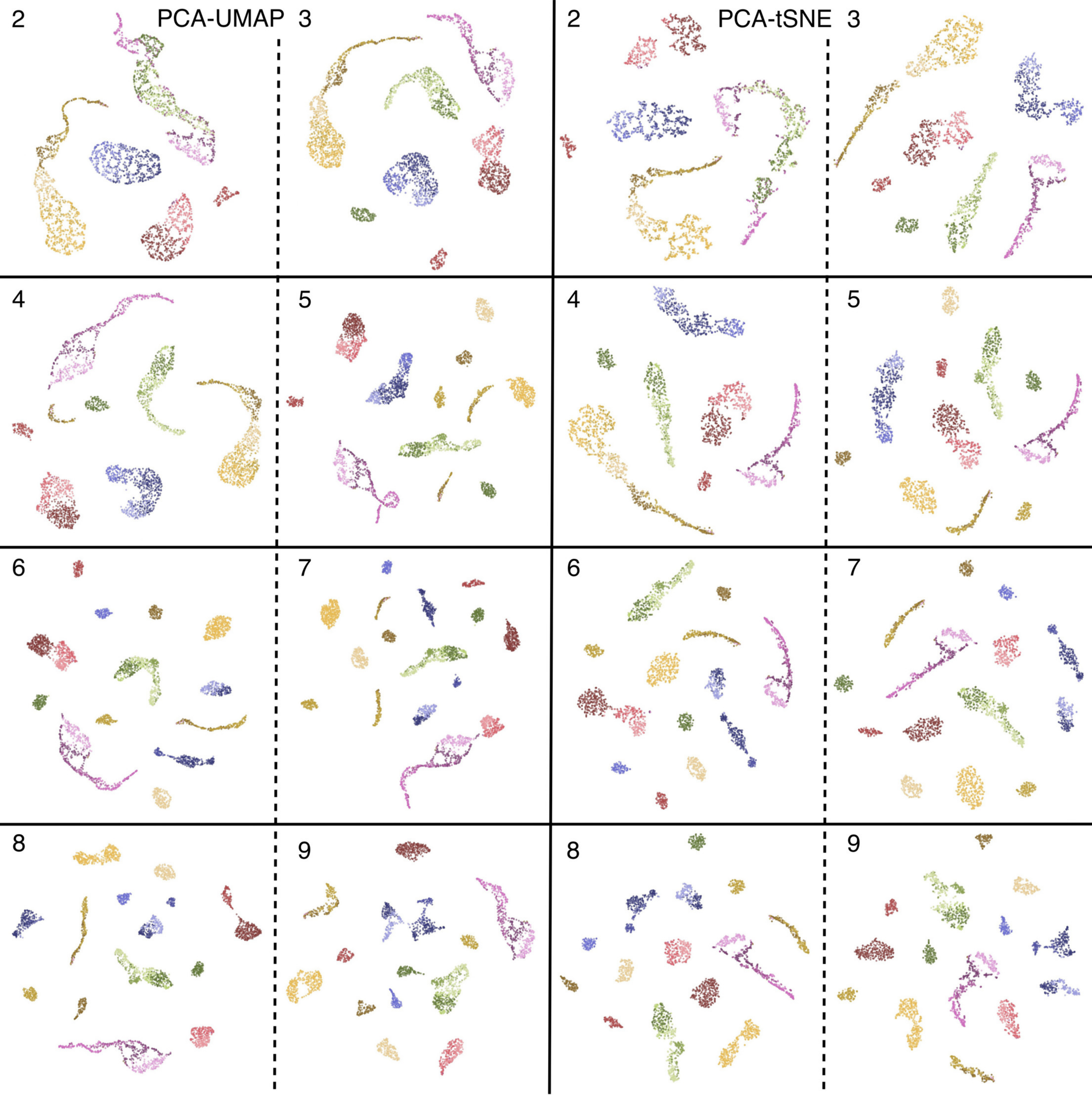

Supplement: S1 Fig — UMAP (left two columns) and t-SNE (right two columns) applied to the top principal components of the 1KGP labelled by the number of components used. Adding more components results in progressively finer population clusters using both methods. (PDF) [file pgen.1008432.s001.pdf]

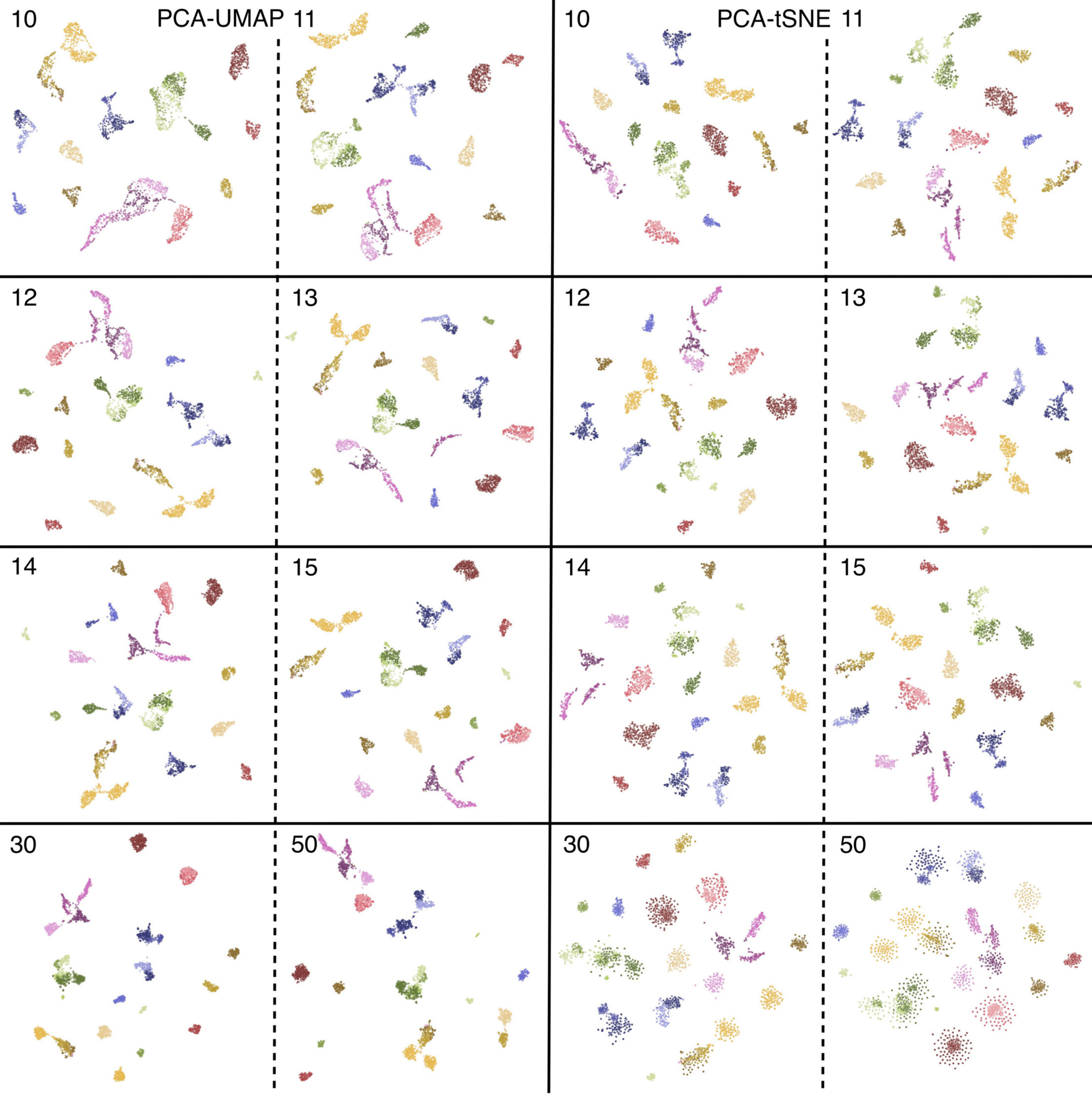

Supplement: S2 Fig — UMAP (left two columns) and t-SNE (right two columns) applied to the top principal components of the 1KGP labelled by the number of components used. Results are similar until approximately 11 components, where t-SNE breaks apart clusters of South Asian (in green) and Central and South American populations (in pink) while UMAP preserves them. At approximately 30 components populations begin to drift together with UMAP and disperse with t-SNE. (PDF) [file pgen.1008432.s002.pdf]

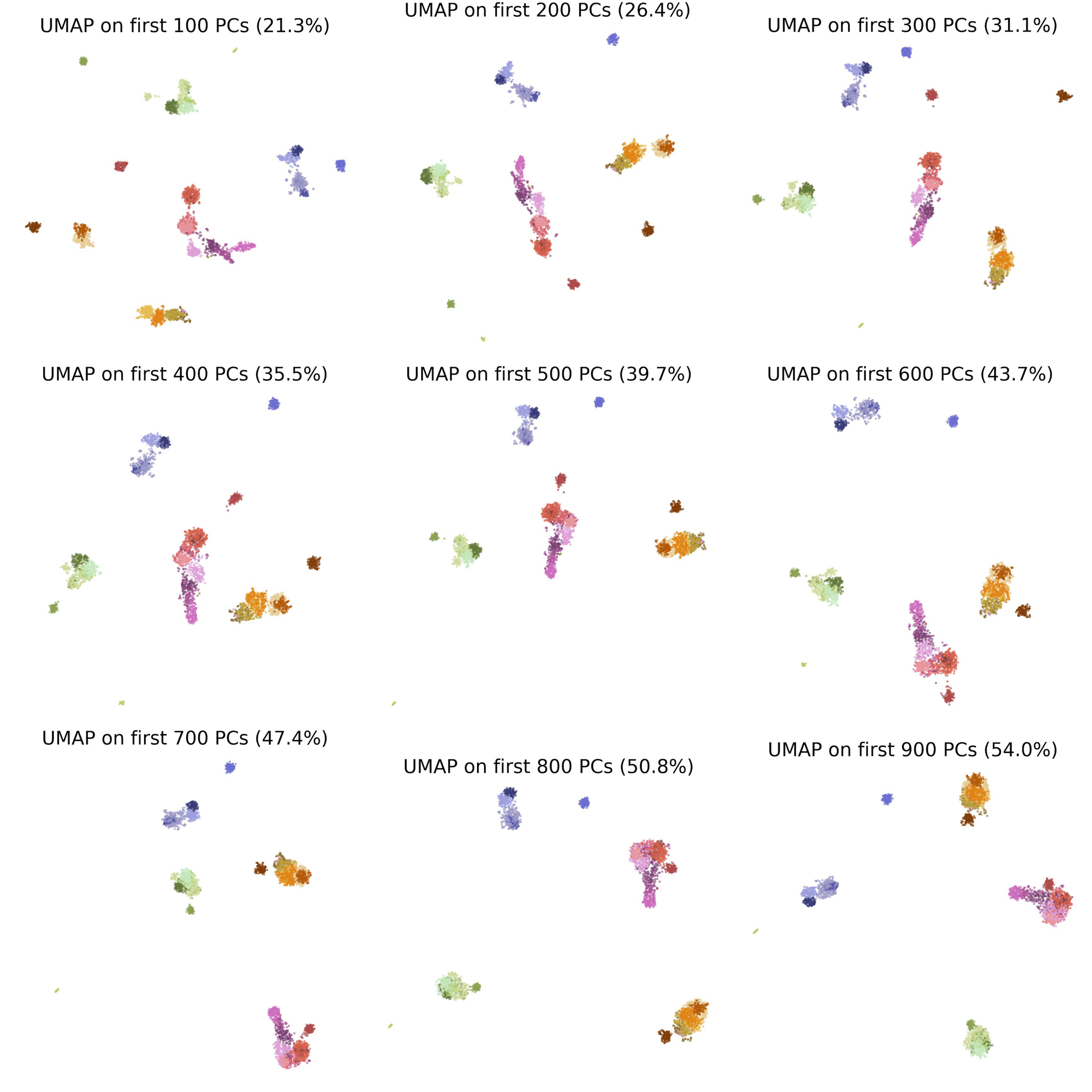

Supplement: S3 Fig — UMAP applied to the first few hundred principal components of the 1KGP data with the amount of variance explained in parentheses. As more components are added, the figure begins to resemble that of UMAP carried out on the full genotype dataset. (JPEG) [file pgen.1008432.s003.jpeg]

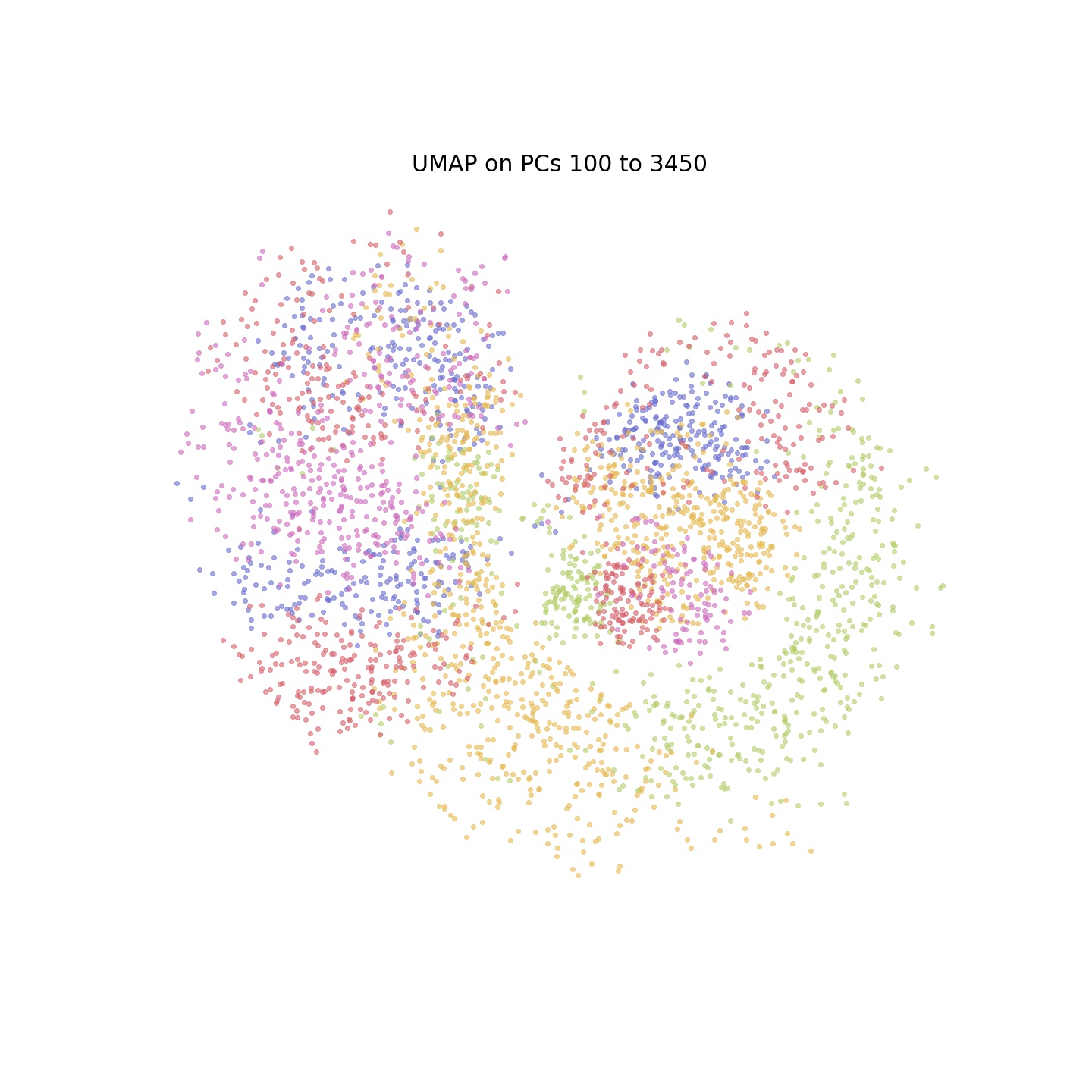

Supplement: S4 Fig — UMAP applied the last 3350 principal components of the 1KGP, which explain 78.7% of the variation. The colour scheme is the same as in Fig 1. (JPEG) [file pgen.1008432.s004.jpeg]

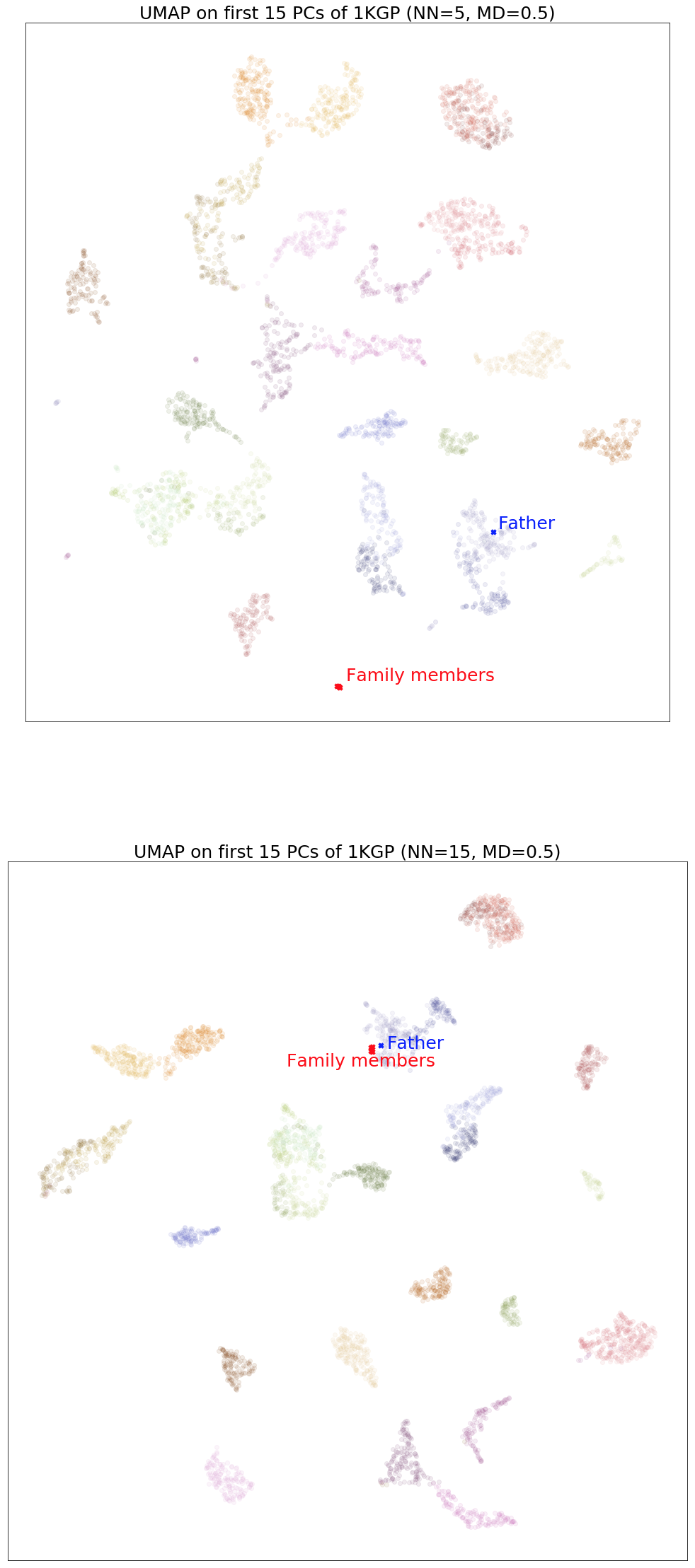

Supplement: S5 Fig — UMAP applied to the first 15 principal components of the 1KGP, with the number of neighbours set to 5 (top) and 15 (bottom). Six members of one Southern Han Chinese family are highlighted: HG00656 (grandfather), HG00657 (grandmother), HG00658 (uncle, mother’s brother), HG00701 (mother), HG00702 (father), HG00703 (child). When using UMAP with five neighbours, the father (in blue) is projected to the cluster of the Southern Han Chinese population while the rest of the family members (in red) form their own disjoint cluster. Using 15 neighbours, the family still clusters together, but as part of the Southern Han Chinese population rather than a separate cluster. (PNG) [file pgen.1008432.s005.png]

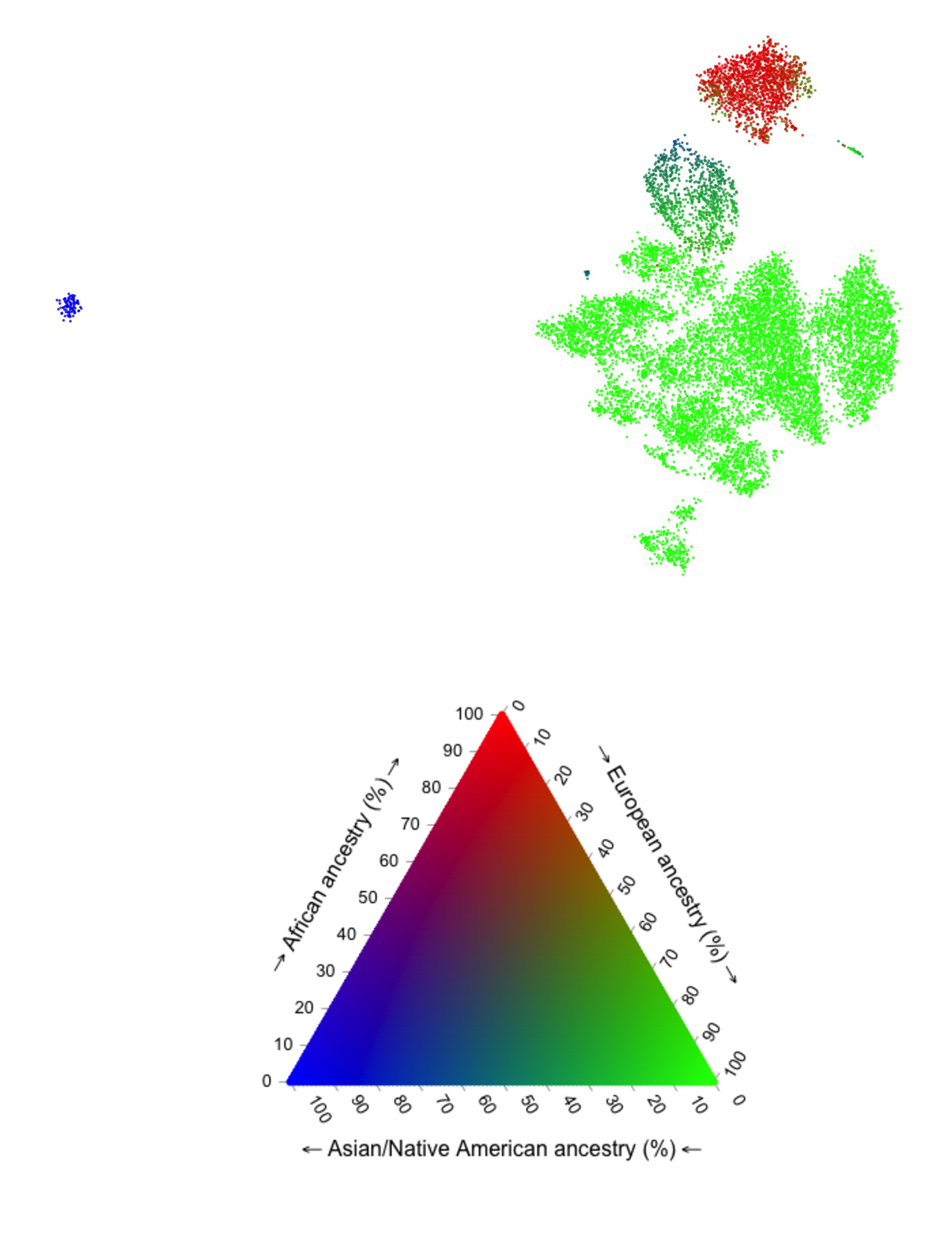

Supplement: S7 Fig — UMAP on the first 10 principal components of HRS data. colouring individuals by estimated admixture from three ancestral populations reveals considerable diversity in the Hispanic population. This projection coloured by self-identified race and Hispanic status is presented in S6 Fig. Admixture proportions for each individual were estimated in (Baharian 2016) by assuming ancestral African, Asian, and European populations using RFMIX. We have scaled each of the three proportions to values between 0 and 255 (with 100% corresponding to 255), to colour individual points by their estimated admixture represented by RGB where red, green, and blue respectively correspond to African, European, and Asian/Native American ancestry. An alternate colouring is provided in S63 Fig. (JPEG) [file pgen.1008432.s007.jpeg]

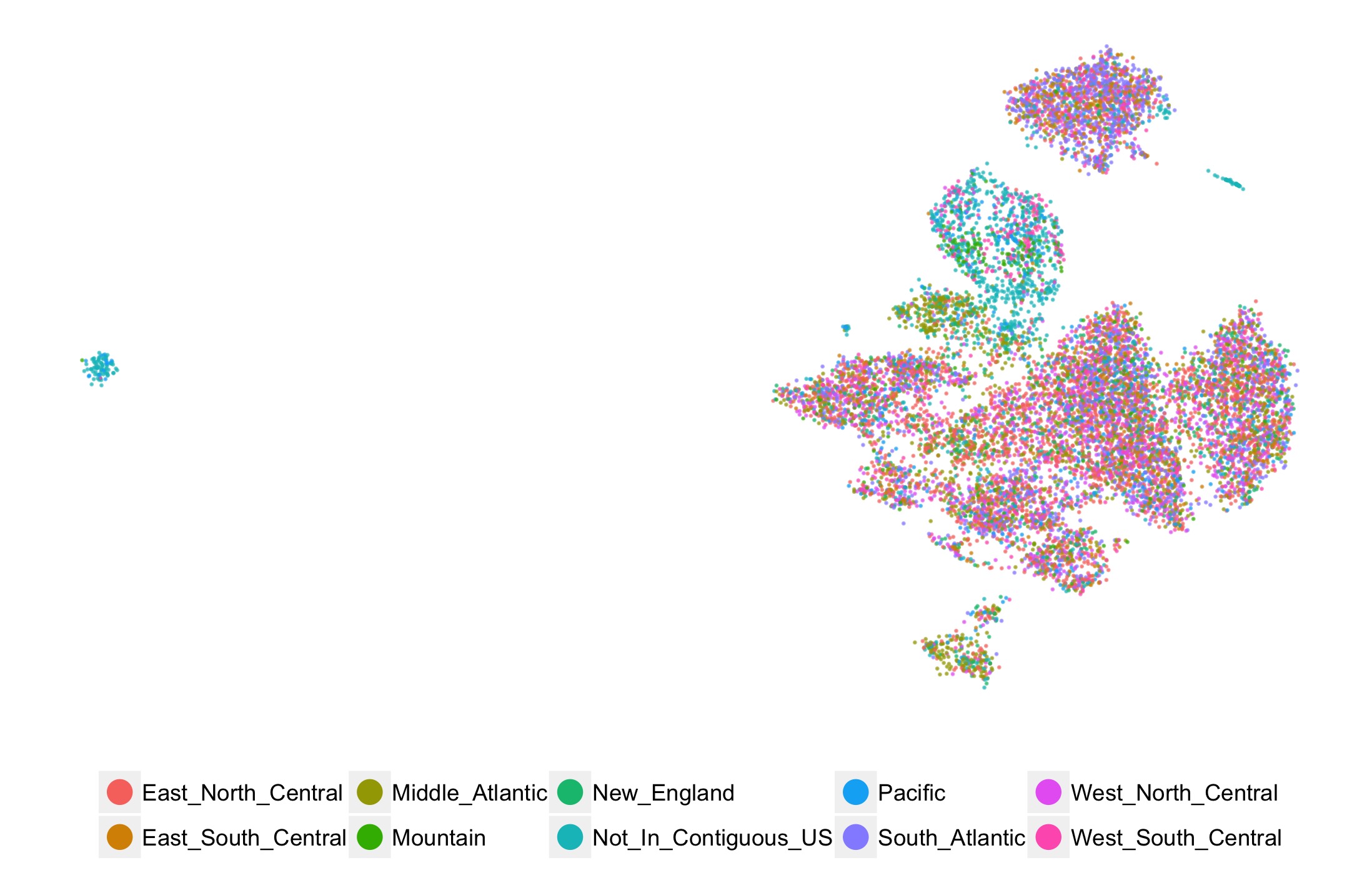

Supplement: S8 Fig — UMAP on the top 10 principal components of the HRS dataset, coloured by Census Bureau birth region. Each colour represents one of the 10 birth regions. There is no obvious pattern in the clusters of majority “White Not Hispanic” individuals. (JPEG) [file pgen.1008432.s008.jpeg]

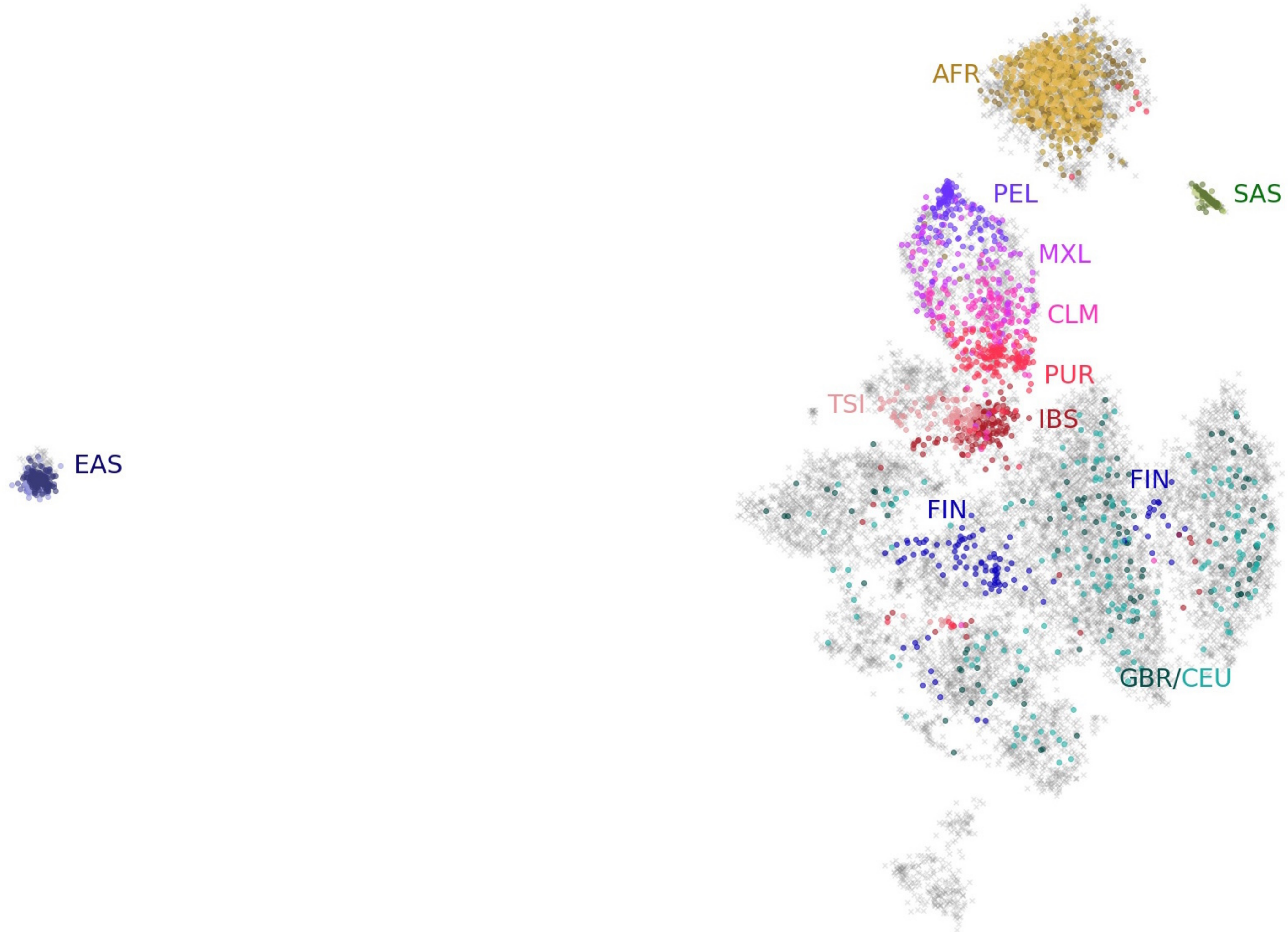

Supplement: S9 Fig — UMAP on the top 10 principal components of the HRS data, with 1KGP data projected onto the embedding. Individuals from the HRS are grey. British (GBR) and other European (CEU) individuals are scattered throughout the “White Not Hispanic” clusters. Finns (FIN) form clear groupings. Spanish (IBS) and Italian (TSI) individuals cluster near the Hispanic grouping. There are sub-groups in the Hispanic cluster formed of Puerto Ricans (PUR), Colombians (CLM), Mexicans (MXL), and Peruvians (PEL). Populations with African ancestry (AFR) appear with Black individuals. East Asian (EAS) populations comprising Chinese, Kinh, and Japanese individuals cluster together with what appears in S7 Fig as a population of mostly Asian ancestry. South Asian (SAS) populations with Indian, Pakistani, and Sri Lankan ancestry cluster in a separate area. One “White Not Hispanic” cluster at the bottom does not cluster with any 1KGP populations. (PDF) [file pgen.1008432.s009.pdf]

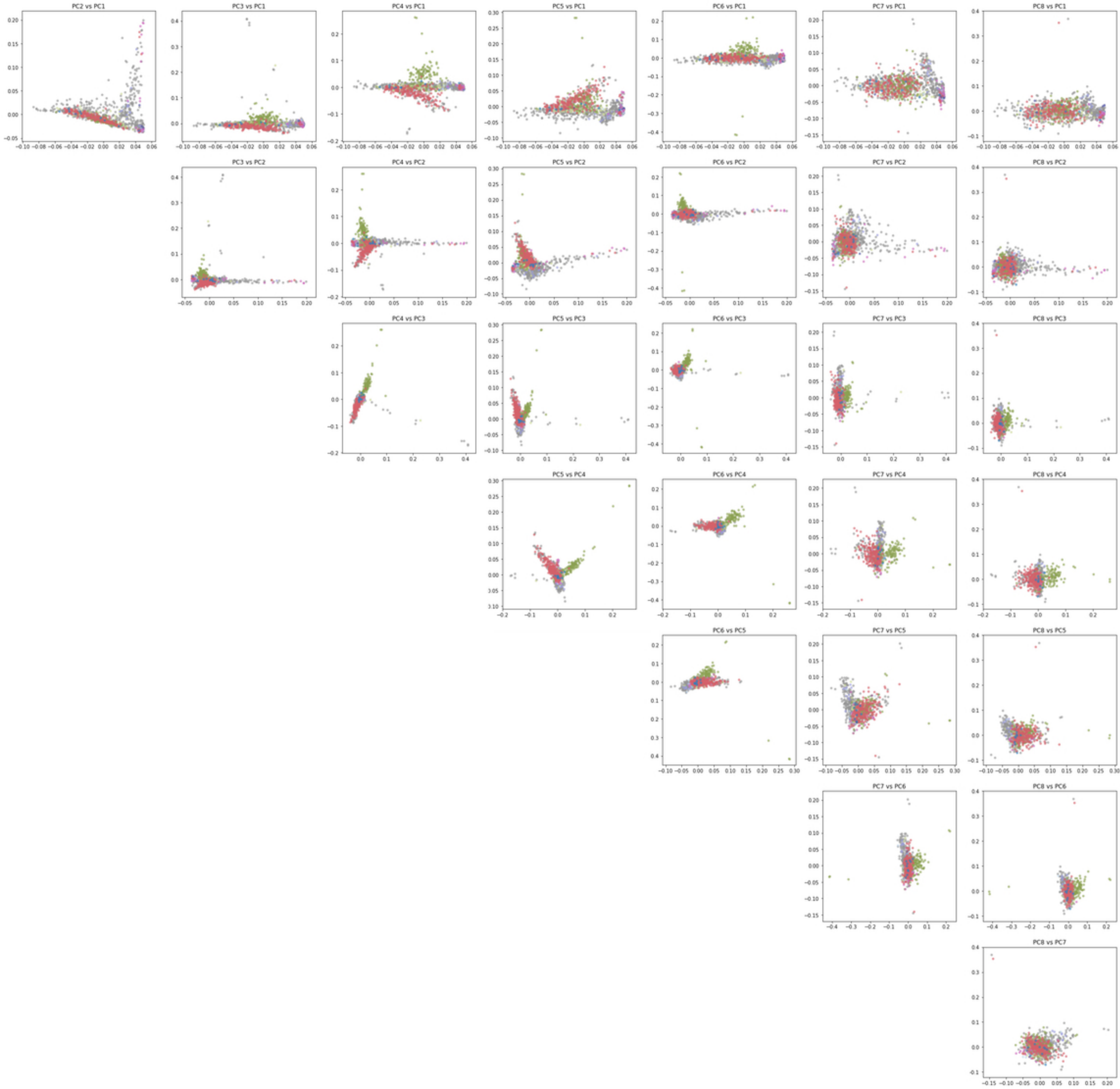

Supplement: S10 Fig — Pairwise plots of the first 8 principal components of the Hispanic subset of the HRS. Those born in the Mountain region are coloured green. (PDF) [file pgen.1008432.s010.pdf]

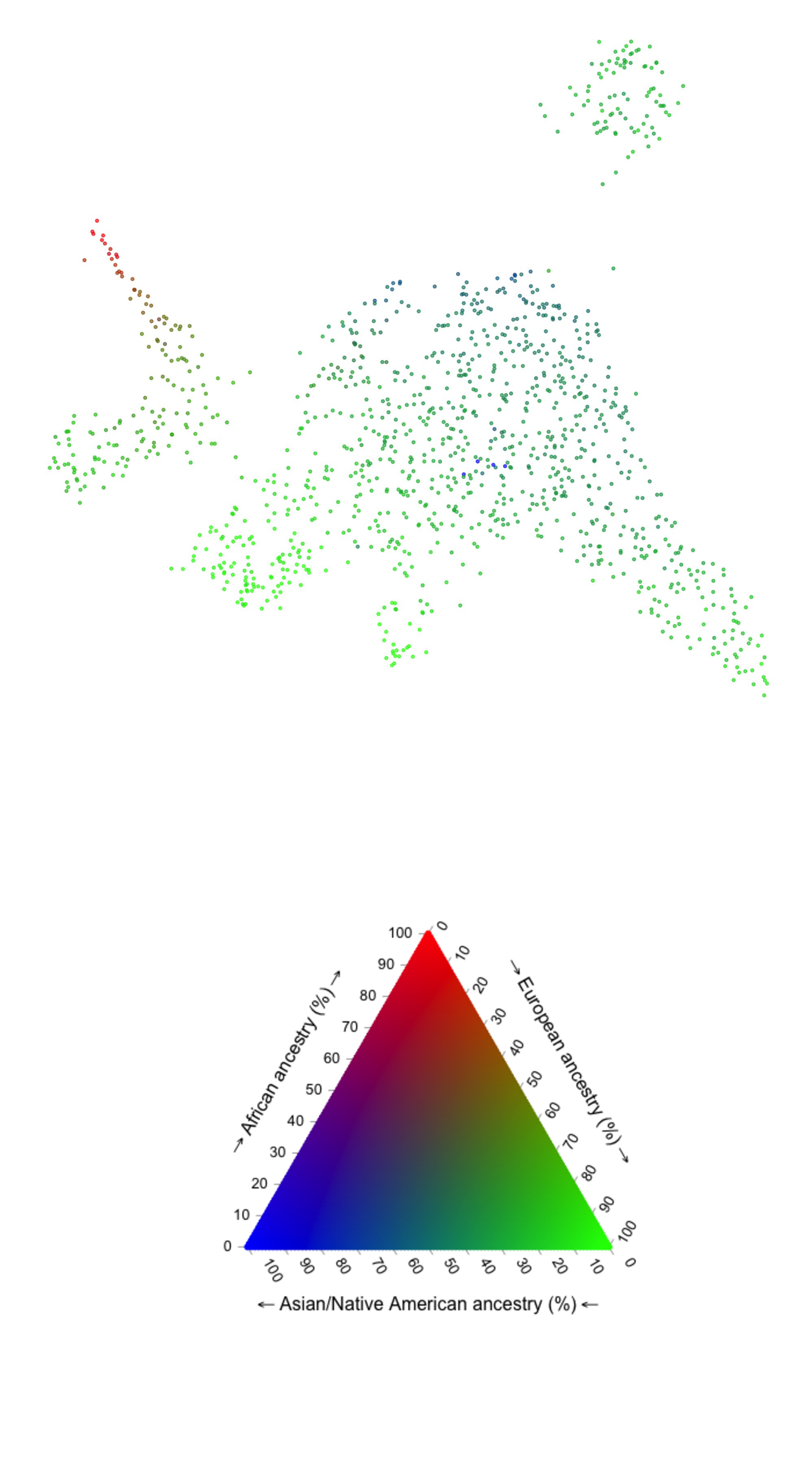

Supplement: S11 Fig — UMAP of the first 7 principal components of the Hispanic population of the HRS, coloured by estimated admixture proportions. Admixture proportions for each individual were estimated in (Baharian, 2016) by assuming ancestral African, Asian, and European populations using RFMIX. We have scaled each of the three proportions to values between 0 and 255 (with 100% corresponding to 255), to colour individual points by their estimated admixture represented by RGB where red, green, and blue respectively correspond to African, European, and Asian/Native American ancestry. An alternate colouring is provided in S64 Fig. (JPEG) [file pgen.1008432.s011.jpeg]

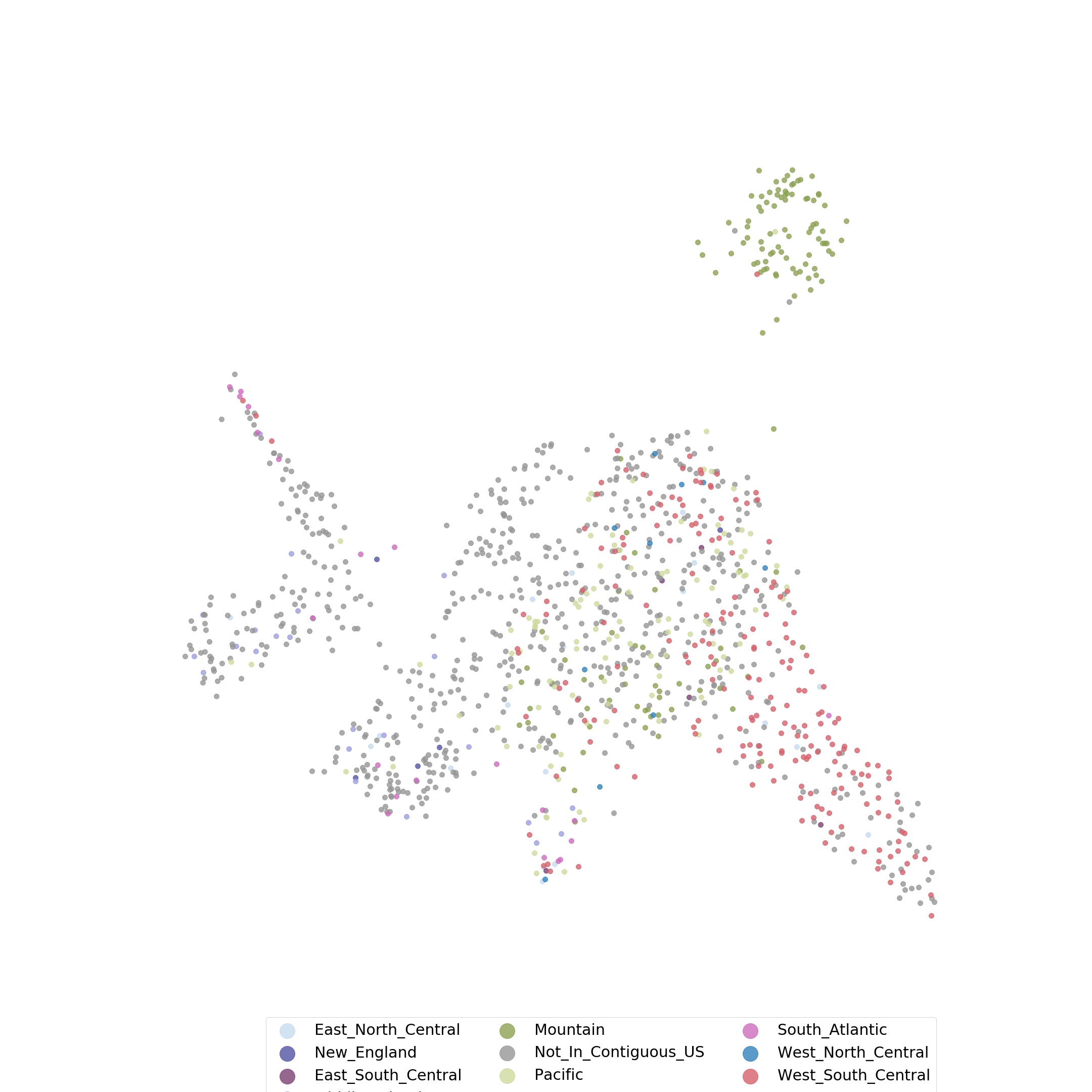

Supplement: S12 Fig — UMAP of the first 7 principal components of the Hispanic population of the HRS, coloured region of birth. (JPEG) [file pgen.1008432.s012.jpeg]

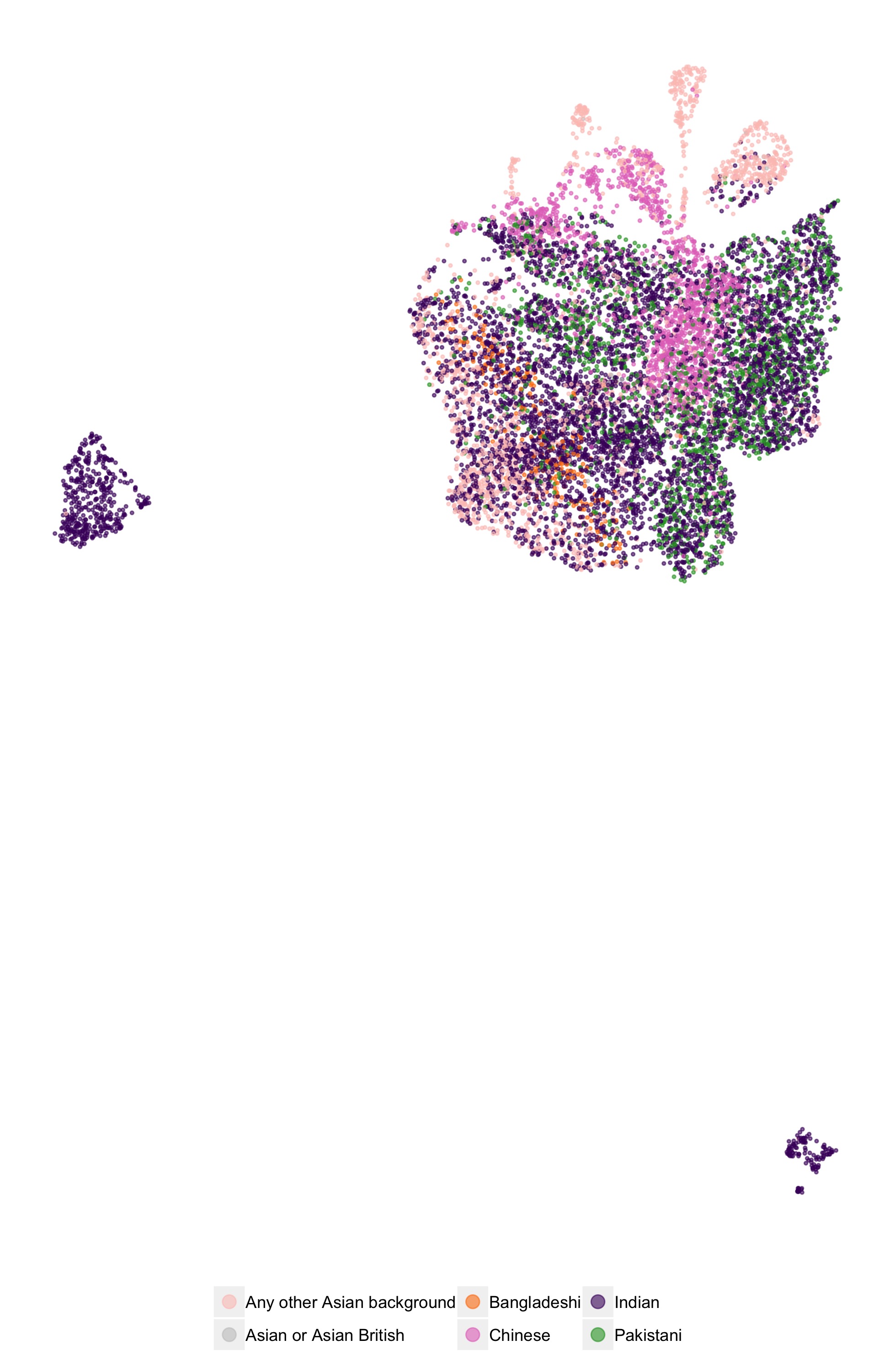

Supplement: S13 Fig — UMAP of the first 8 principal components of the Asian population in the UKBB coloured by self-identified ethnicity. This is an alternate colouring of Fig 2B. (JPEG) [file pgen.1008432.s013.jpeg]

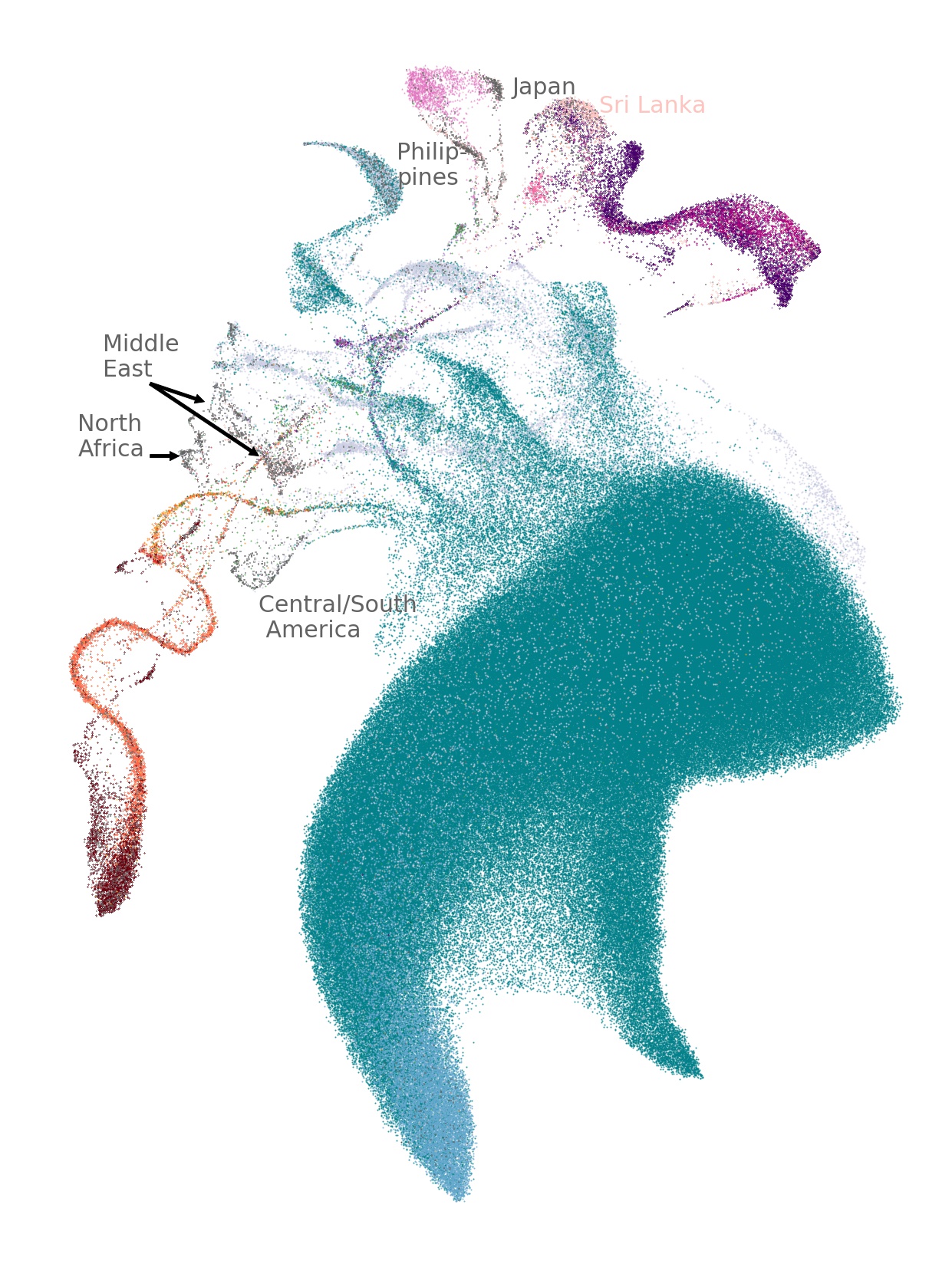

Supplement: S14 Fig — Using country of birth data, some of the larger unidentified groups from Fig 3B were identified as being born mostly in Japan, the Philippines, North Africa, the Middle East, and Central and South America. The large cluster of “Any other Asian Background” were mostly born in Sri Lanka. (JPEG) [file pgen.1008432.s014.jpeg]

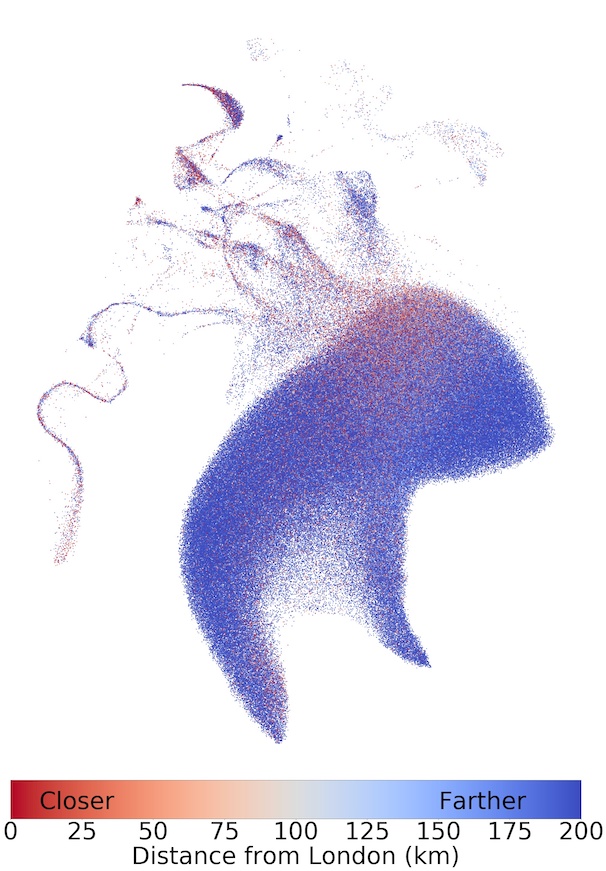

Supplement: S15 Fig — UMAP on UKBB data, coloured by distance from London, with red representing those living closer to London and blue representing those living farther from London. A 200km radius extends roughly to Cardiff, and a 100km radius extends roughly to cities such as Leicester and Bath, and contains cities such as Oxford, Cambridge, and Peterborough. Data has been randomized as explained in the materials and methods section. (JPEG) [file pgen.1008432.s015.jpeg]

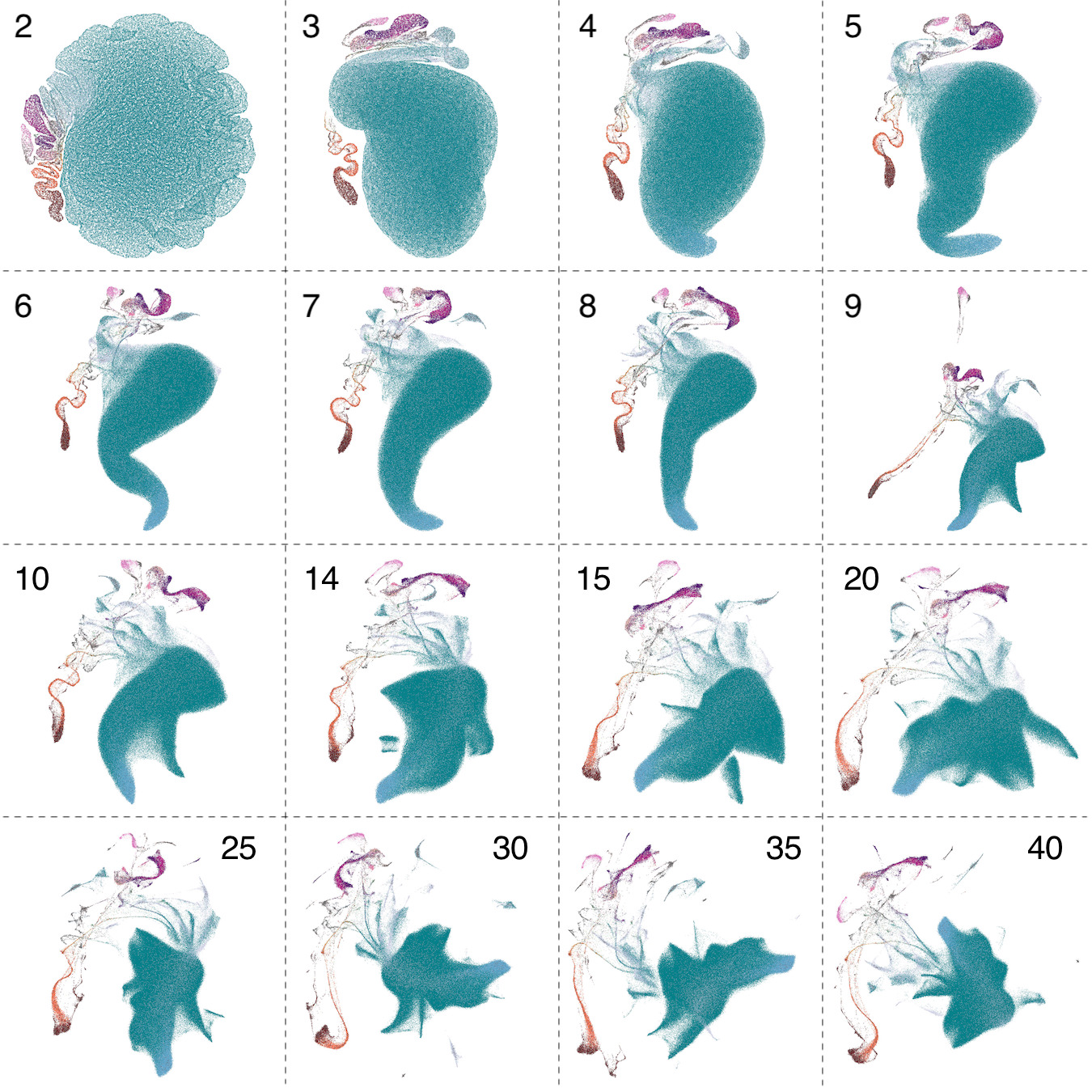

Supplement: S16 Fig — UMAP on UKBB data, coloured by self-identified ethnic background. Images are labelled by the number of components included. (JPEG) [file pgen.1008432.s016.jpeg]

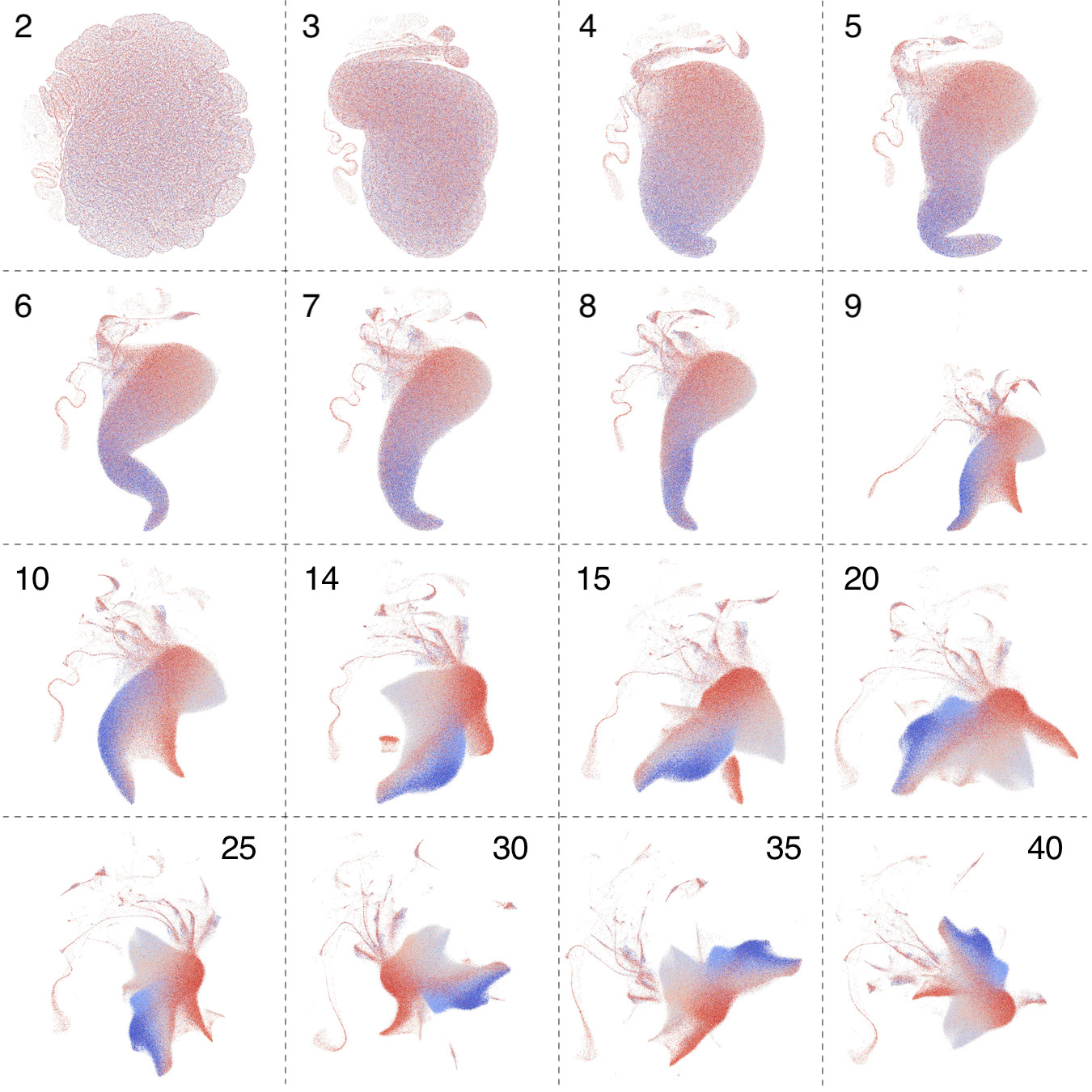

Supplement: S17 Fig — UMAP on UKBB data, coloured by northing values, with more blue representing more northern coordinates and more red representing more southern coordinates. Images are labelled by the number of components included. Data has been randomized as explained in the materials and methods section. (JPEG) [file pgen.1008432.s017.jpeg]

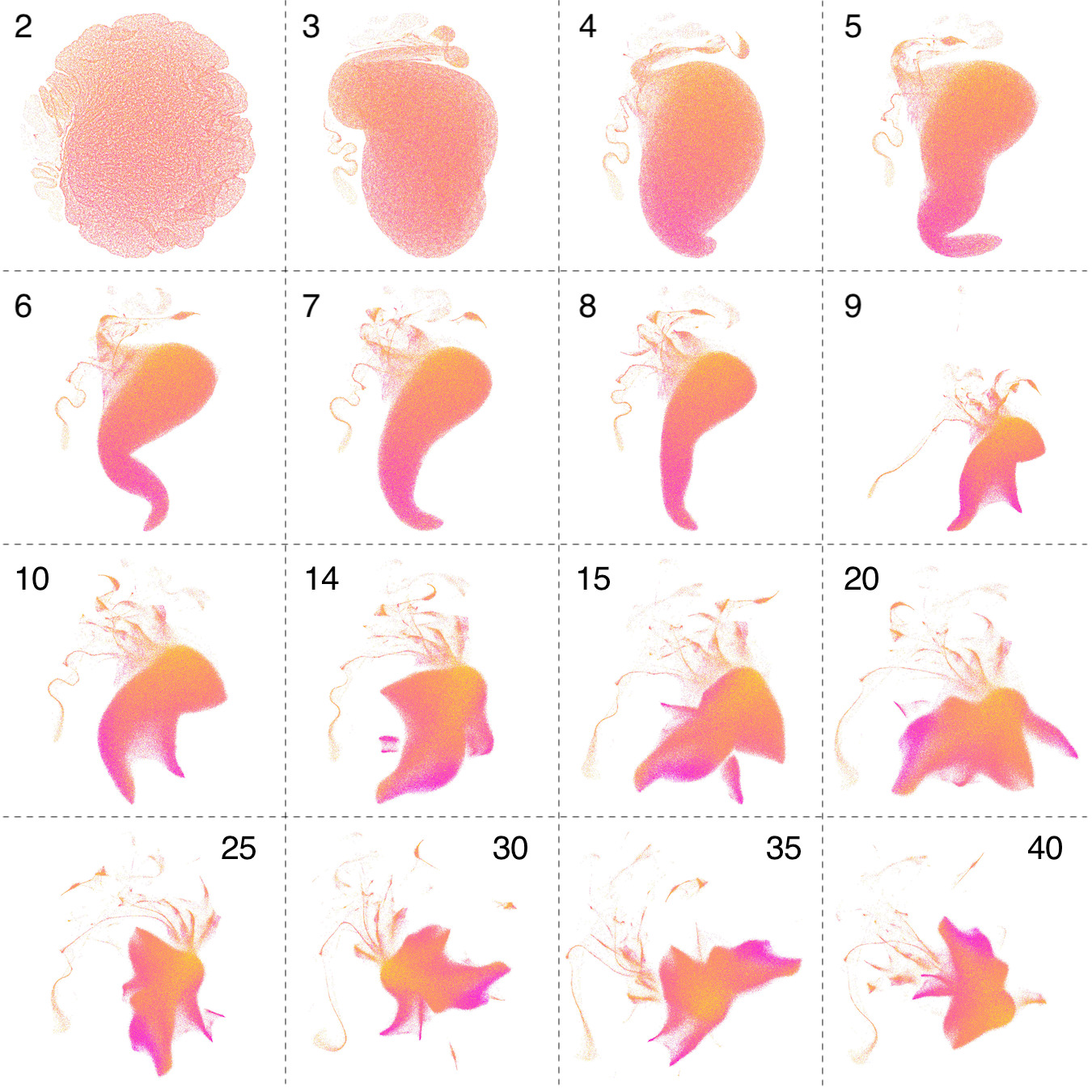

Supplement: S18 Fig — UMAP on UKBB data, coloured by easting values, with more yellow representing more eastern coordinates and more pink representing more western coordinates. Images are labelled by the number of components included. Data has been randomized as explained in the materials and methods section. (JPEG) [file pgen.1008432.s018.jpeg]

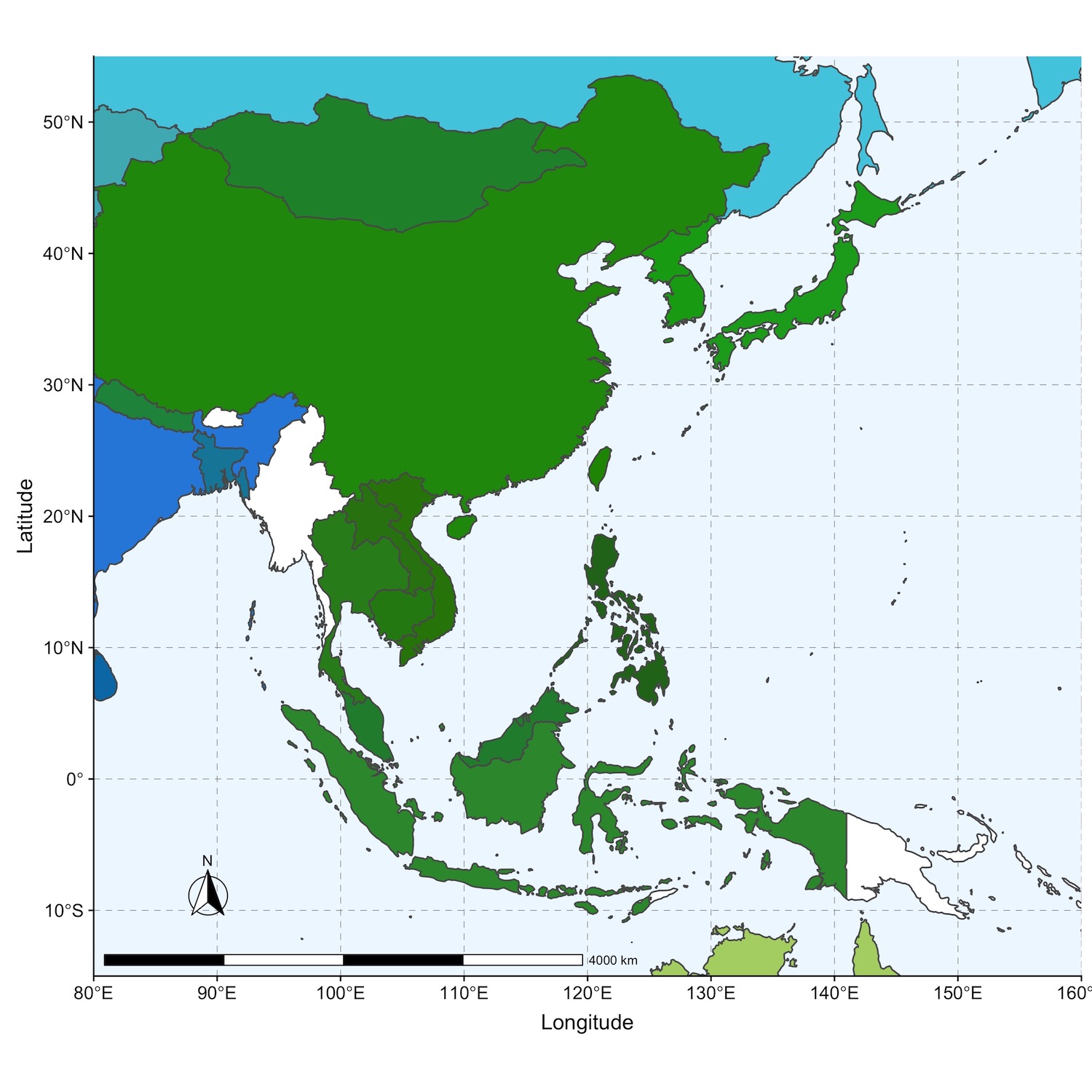

Supplement: S19 Fig — Fig 5b, zoomed in on Asia. Geographic distribution of UMAP coordinates. Using the country of birth of individuals in the UKBB, we colour countries by the closeness in 3D UMAP space of those born there. Broad patterns of similarity appear in East Asia, South Asia, North African and the Middle East, West Africa, and South America. Differences between neighbouring countries can reflect both ancient population structure and recent differences in migration history. Evidence of migrations related to colonialism are visible with, e.g., European ancestry in South Africa and South Asian ancestry in Kenya and Tanzania. Because of the large number of White British individuals born abroad, to avoid skewing the colour scale they were not included unless they were born in the UK, Europe, Australia, Canada, or the United States, where UKBB participants already tended to have European ancestry. (JPG) [file pgen.1008432.s019.jpg]

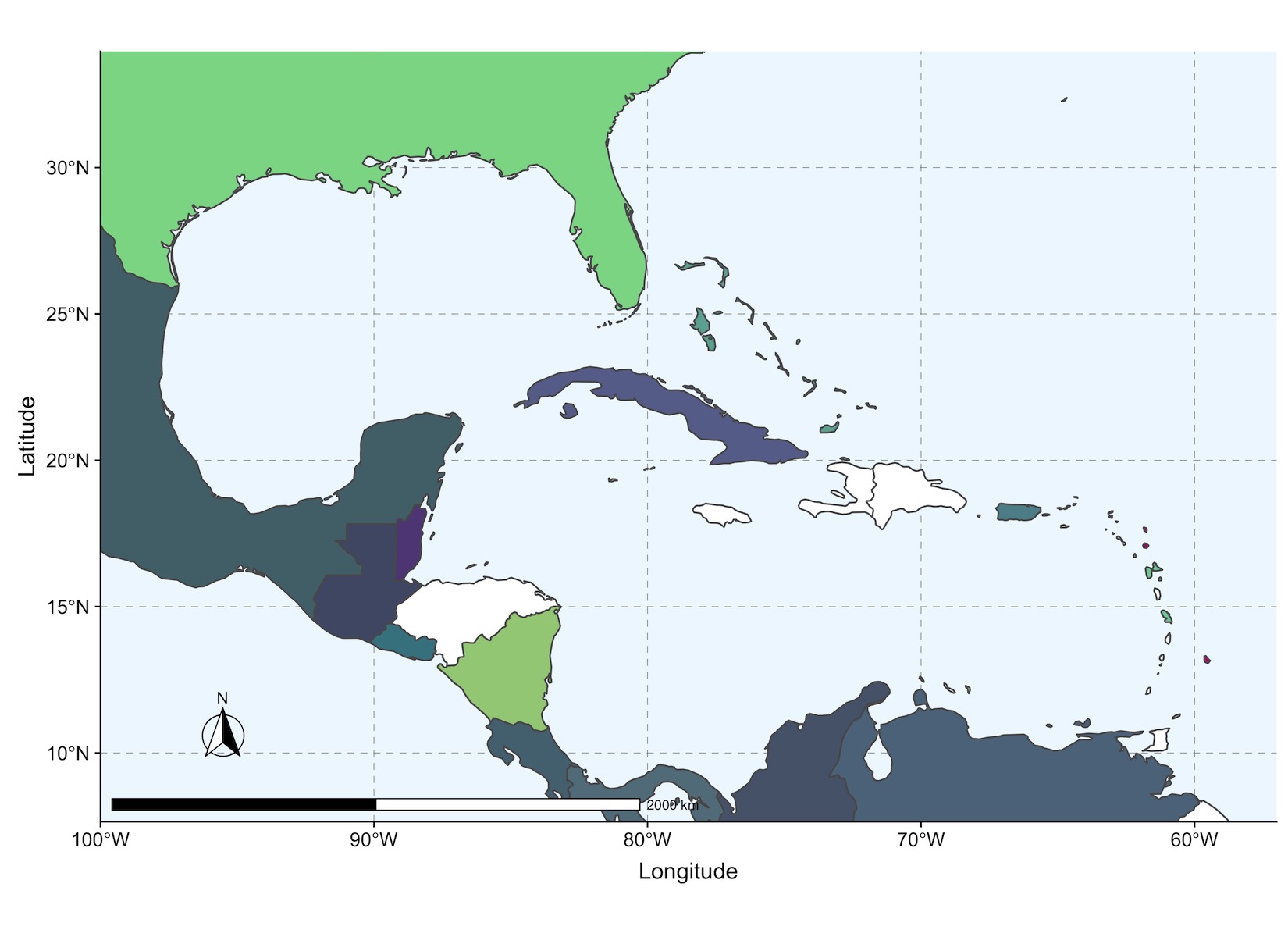

Supplement: S20 Fig — Fig 5b, zoomed in on the Caribbean. Geographic distribution of UMAP coordinates. Using the country of birth of individuals in the UKBB, we colour countries by the closeness in 3D UMAP space of those born there. Broad patterns of similarity appear in East Asia, South Asia, North African and the Middle East, West Africa, and South America. Differences between neighbouring countries can reflect both ancient population structure and recent differences in migration history. Evidence of migrations related to colonialism are visible with, e.g., European ancestry in South Africa and South Asian ancestry in Kenya and Tanzania. Because of the large number of White British individuals born abroad, to avoid skewing the colour scale they were not included unless they were born in the UK, Europe, Australia, Canada, or the United States, where UKBB participants already tended to have European ancestry. (JPG) [file pgen.1008432.s020.jpg]

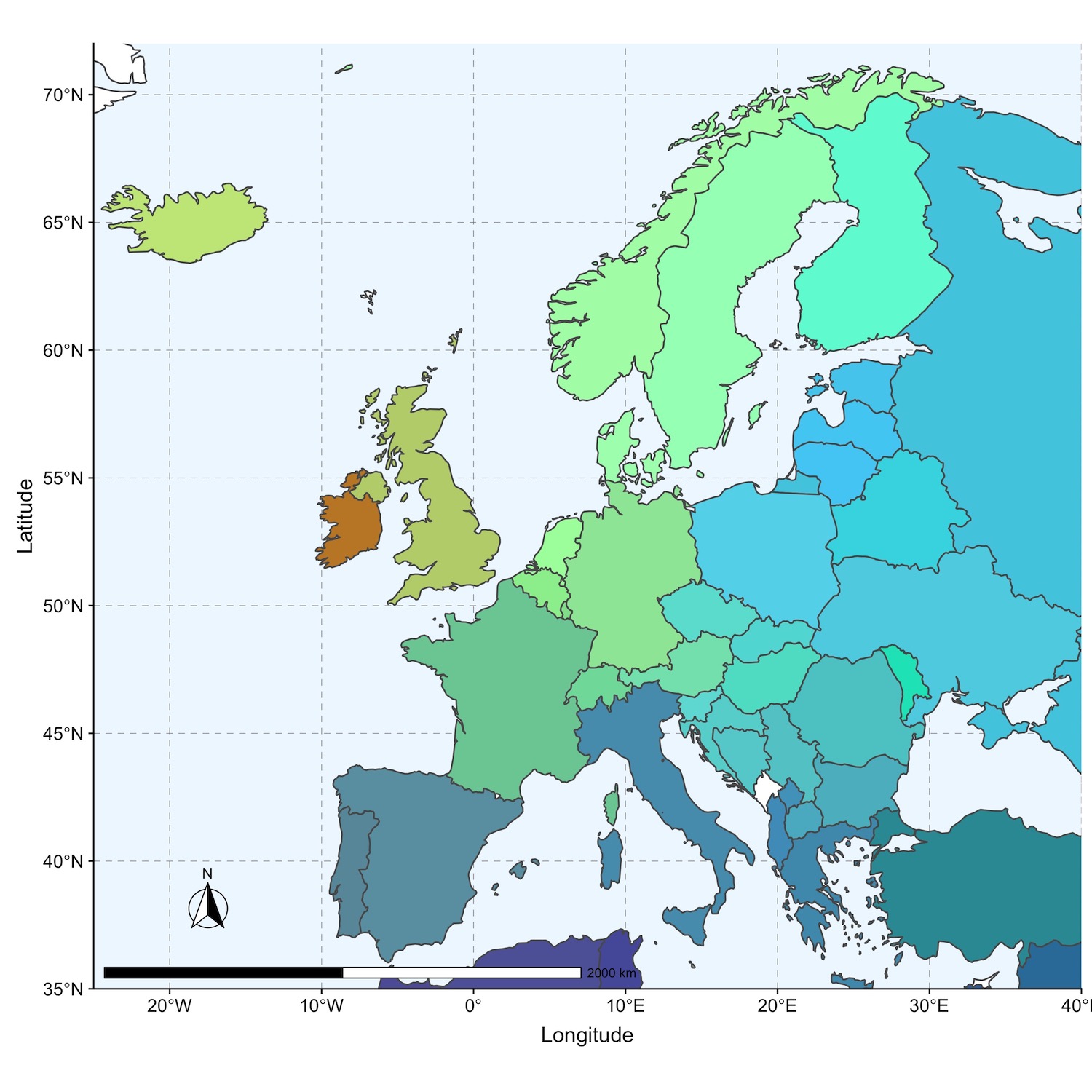

Supplement: S21 Fig — Fig 5b, zoomed in on Europe. Geographic distribution of UMAP coordinates. Using the country of birth of individuals in the UKBB, we colour countries by the closeness in 3D UMAP space of those born there. Broad patterns of similarity appear in East Asia, South Asia, North African and the Middle East, West Africa, and South America. Differences between neighbouring countries can reflect both ancient population structure and recent differences in migration history. Evidence of migrations related to colonialism are visible with, e.g., European ancestry in South Africa and South Asian ancestry in Kenya and Tanzania. Because of the large number of White British individuals born abroad, to avoid skewing the colour scale they were not included unless they were born in the UK, Europe, Australia, Canada, or the United States, where UKBB participants already tended to have European ancestry. (JPG) [file pgen.1008432.s021.jpg]

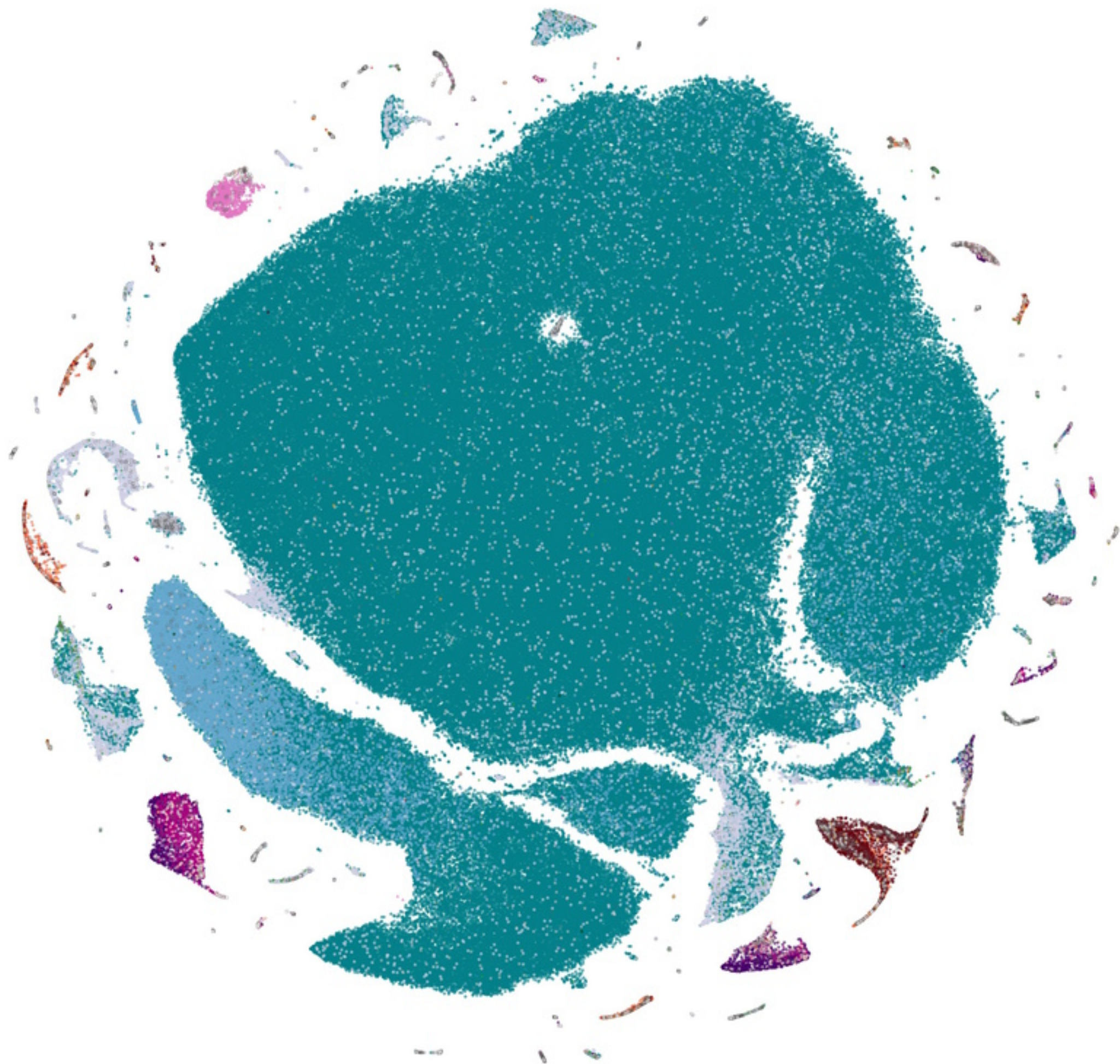

Supplement: S22 Fig — t-SNE applied to the top 10 principal components of the UKBB, coloured by ethnic background. The unbalanced populations resulted in many individuals and populations being orphaned along the periphery of the main cluster. (PDF) [file pgen.1008432.s022.pdf]

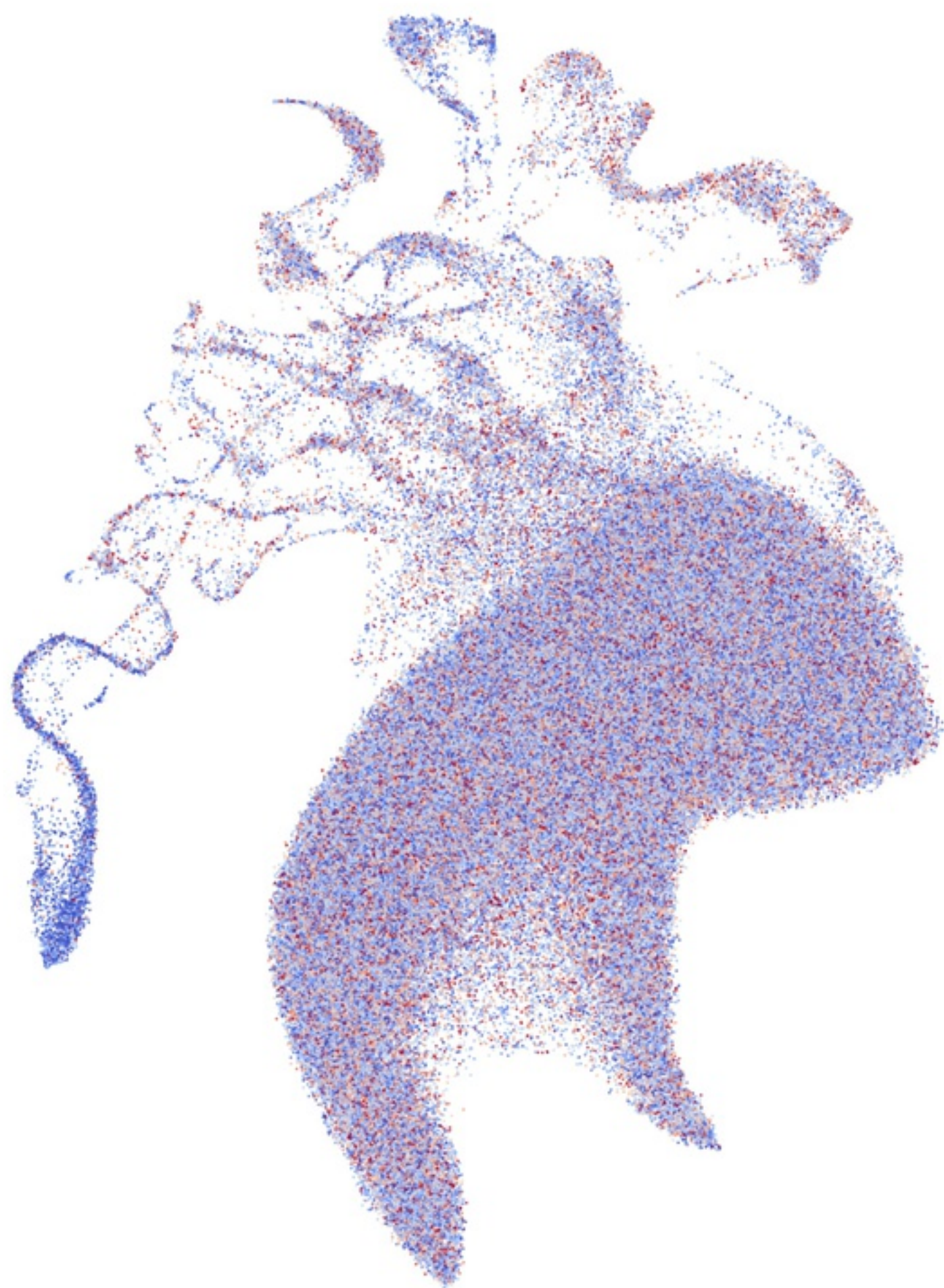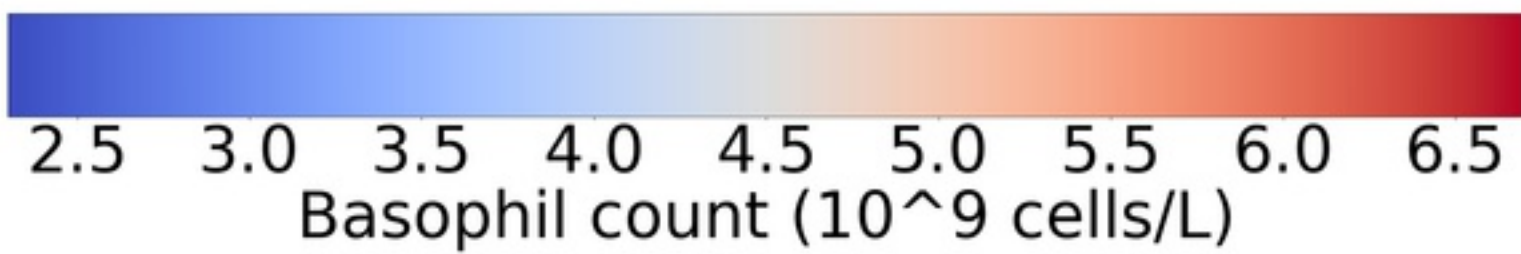

Supplement: S23 Fig — UMAP on the top 10 principal components of the UKBB coloured by basophil count (female). Data has been randomized as explained in the materials and methods section. (PDF) [file pgen.1008432.s023.pdf]

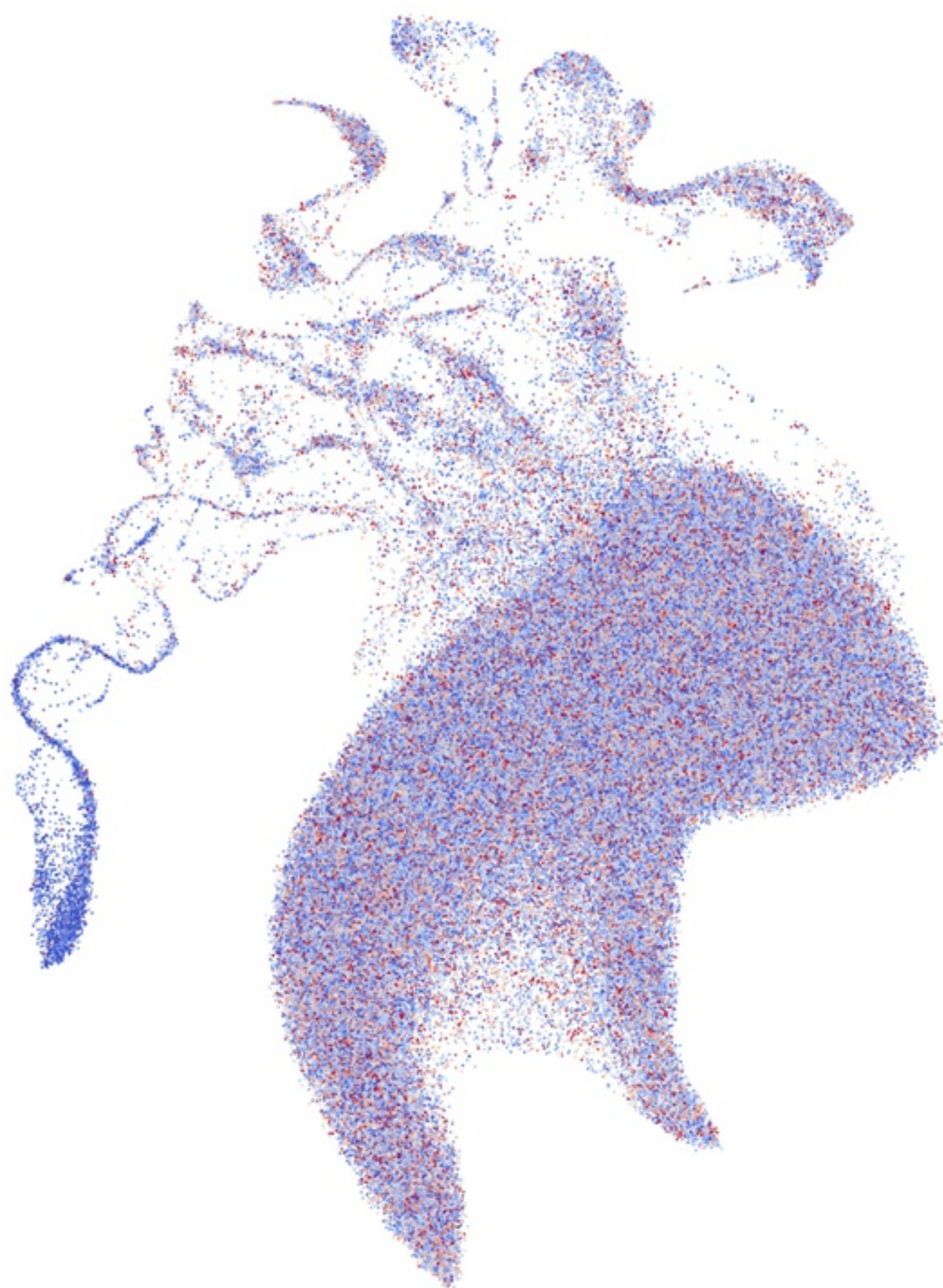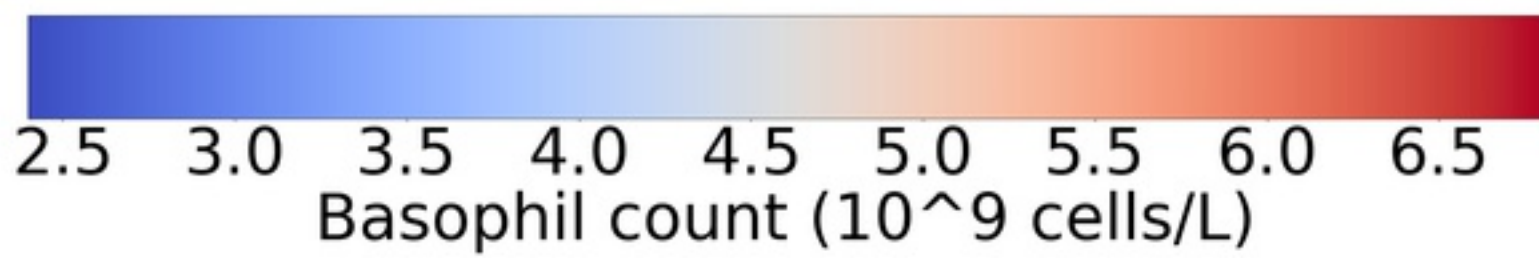

Supplement: S24 Fig — UMAP on the top 10 principal components of the UKBB coloured by basophil count (male). Data has been randomized as explained in the materials and methods section. (PDF) [file pgen.1008432.s024.pdf]

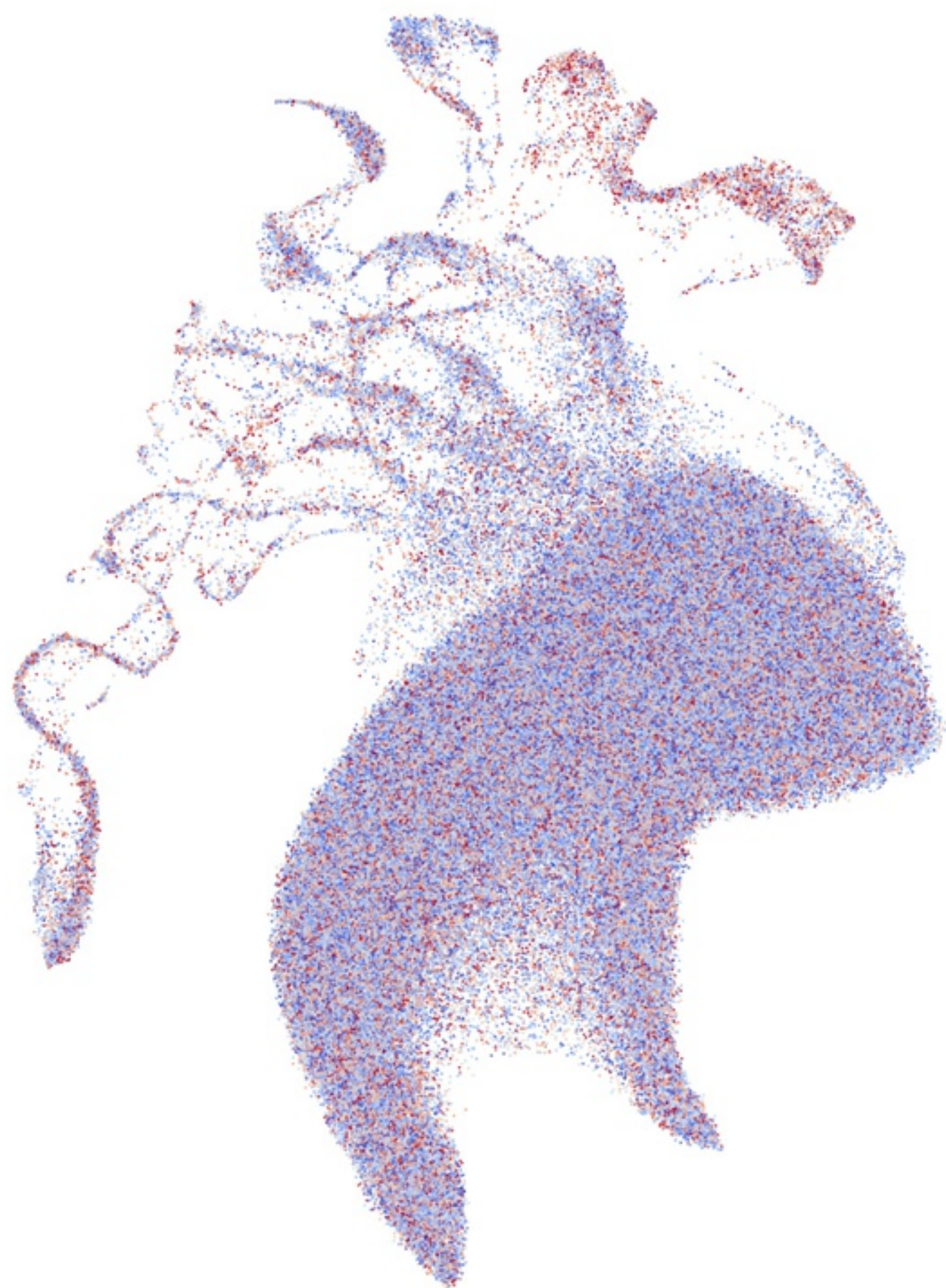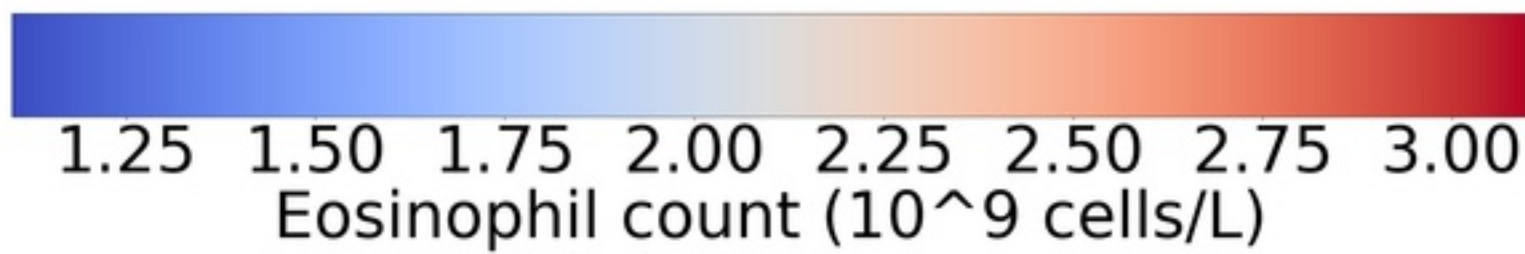

Supplement: S25 Fig — UMAP on the top 10 principal components of the UKBB coloured by eosinophil count (female). Data has been randomized as explained in the materials and methods section. (PDF) [file pgen.1008432.s025.pdf]

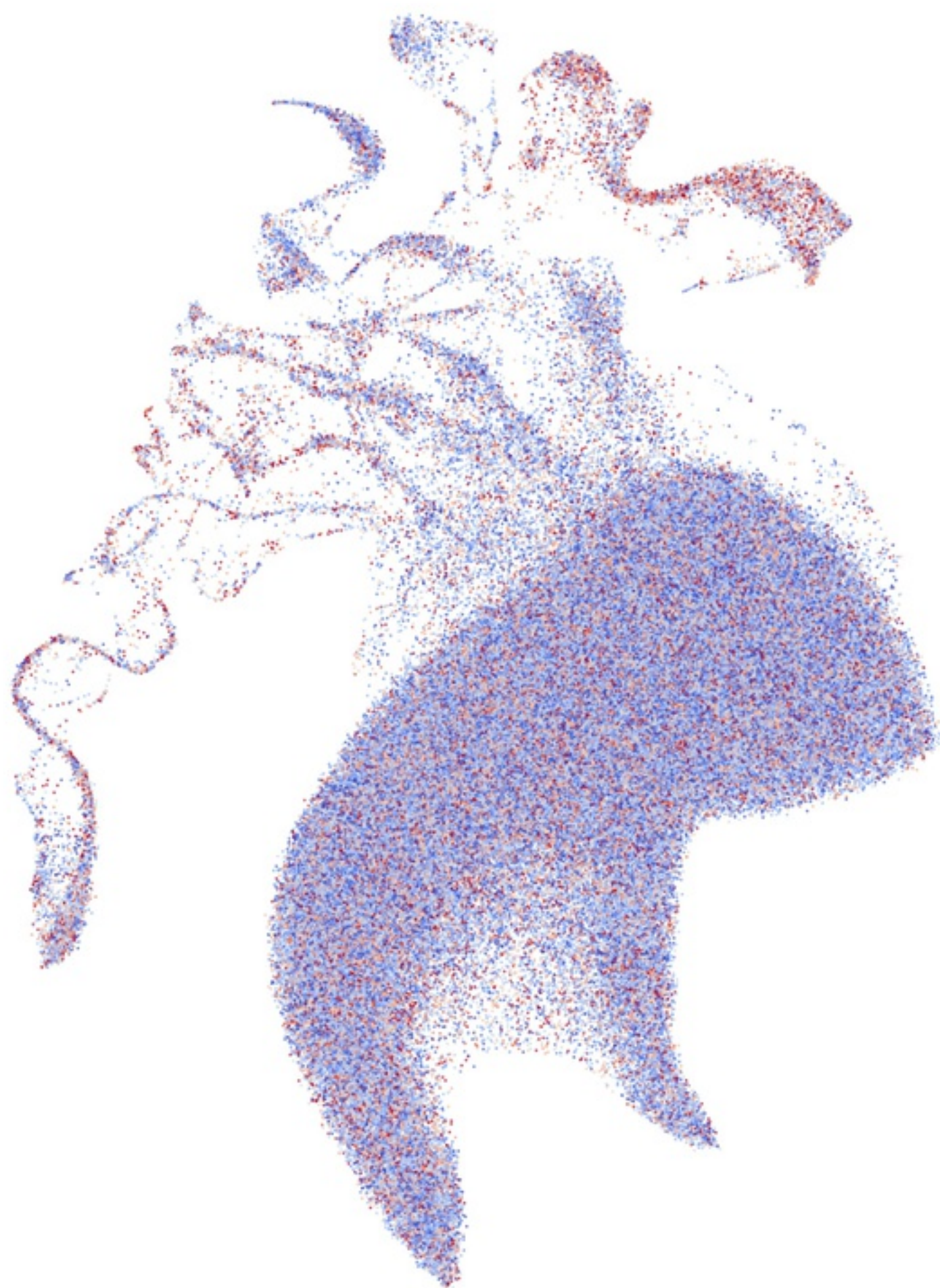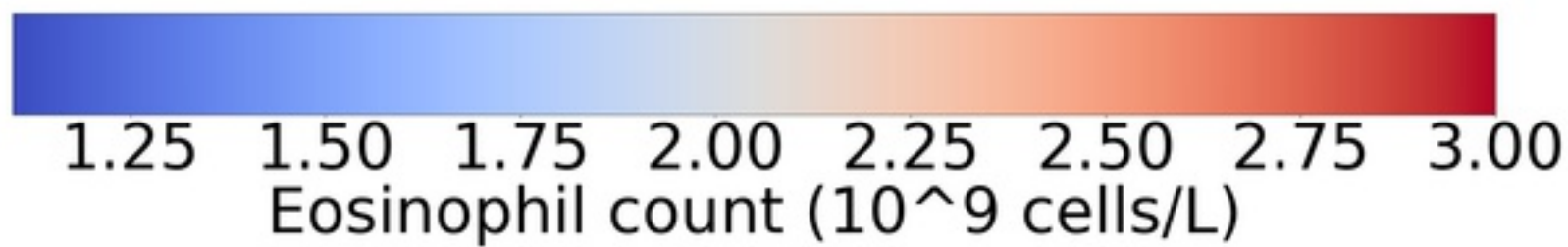

Supplement: S26 Fig — UMAP on the top 10 principal components of the UKBB coloured by eosinophil count (male). Data has been randomized as explained in the materials and methods section. (PDF) [file pgen.1008432.s026.pdf]

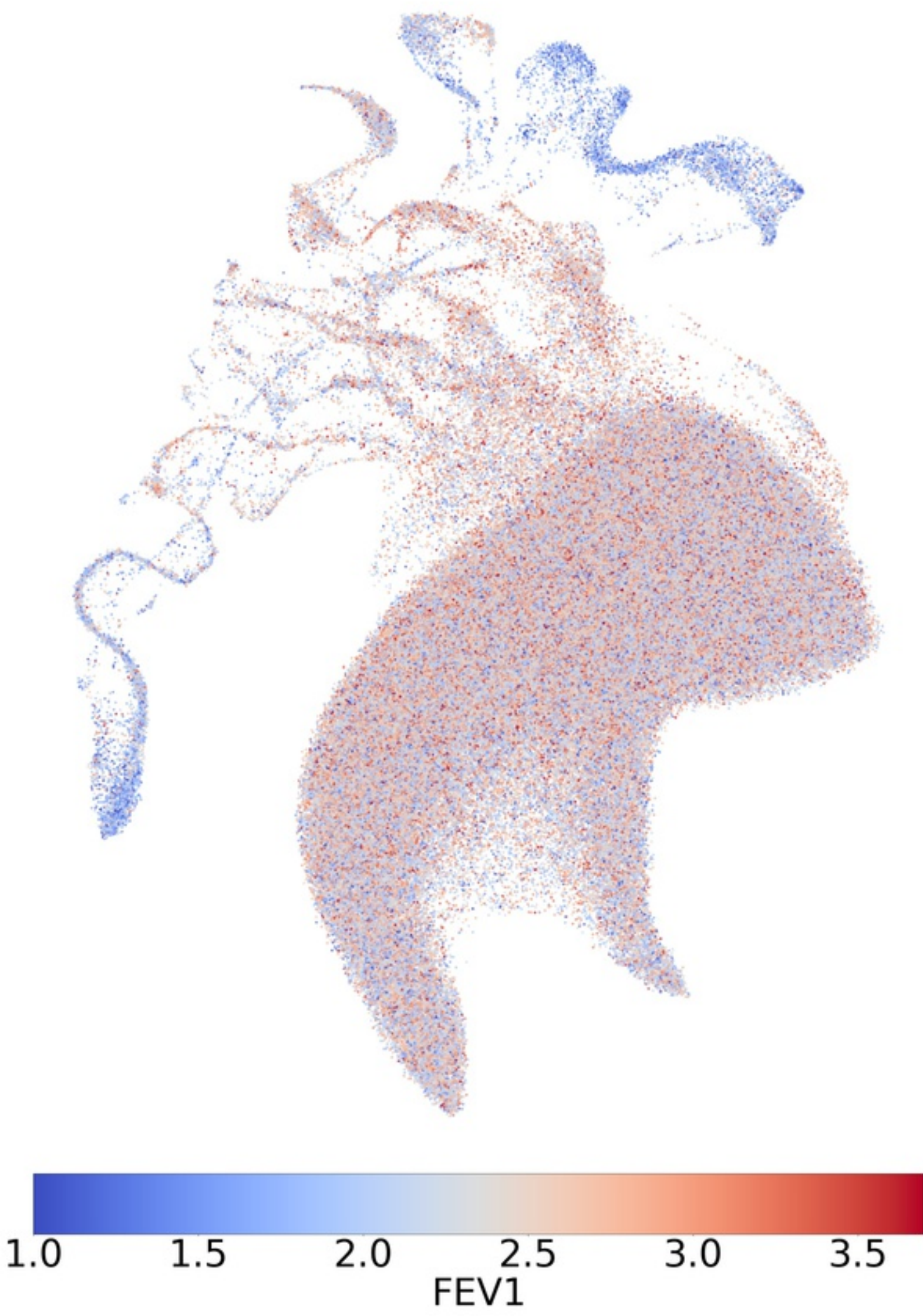

Supplement: S27 Fig — UMAP on the top 10 principal components of the UKBB coloured by FEV1 (female). Data has been randomized as explained in the materials and methods section. (PDF) [file pgen.1008432.s027.pdf]

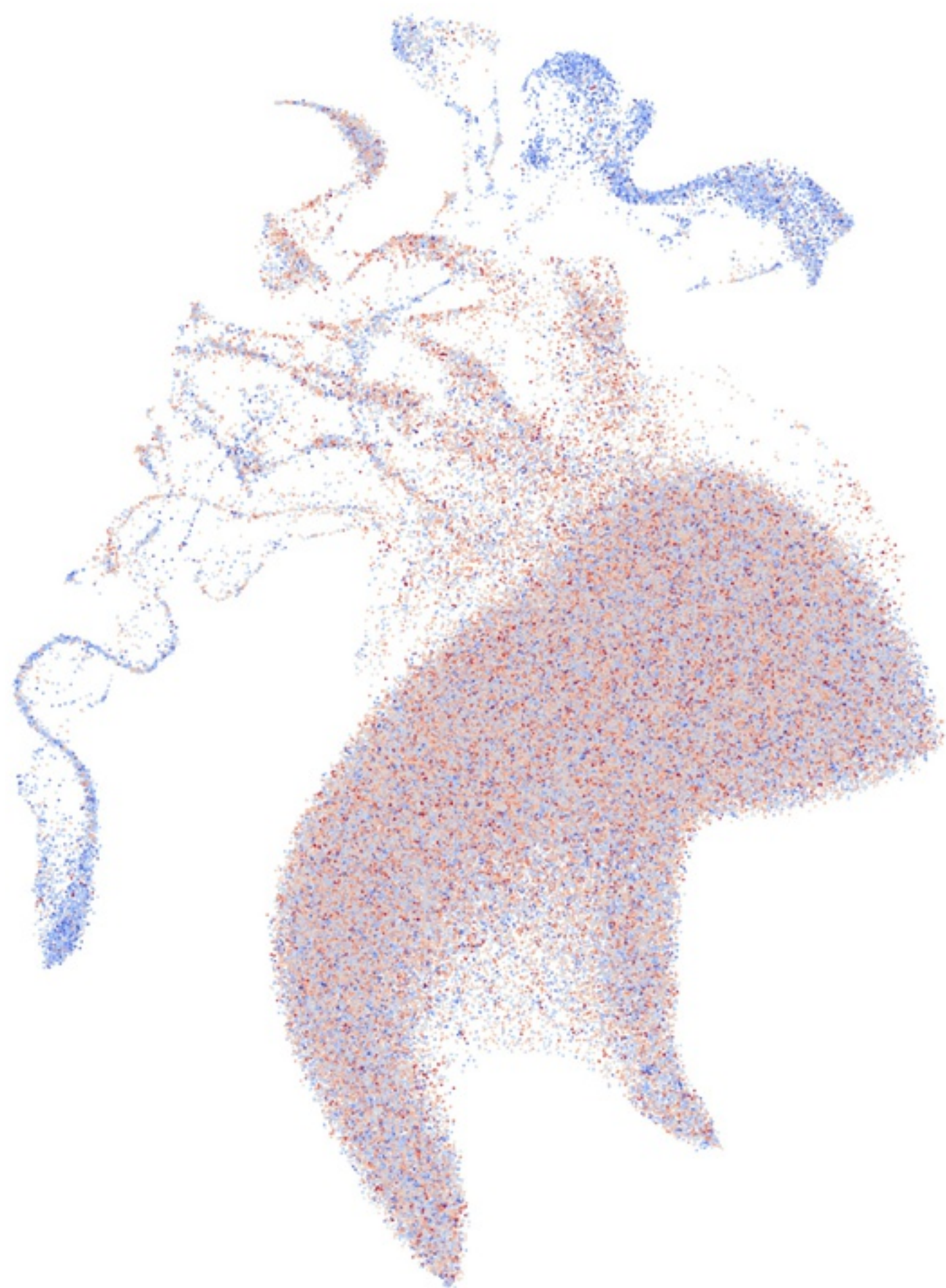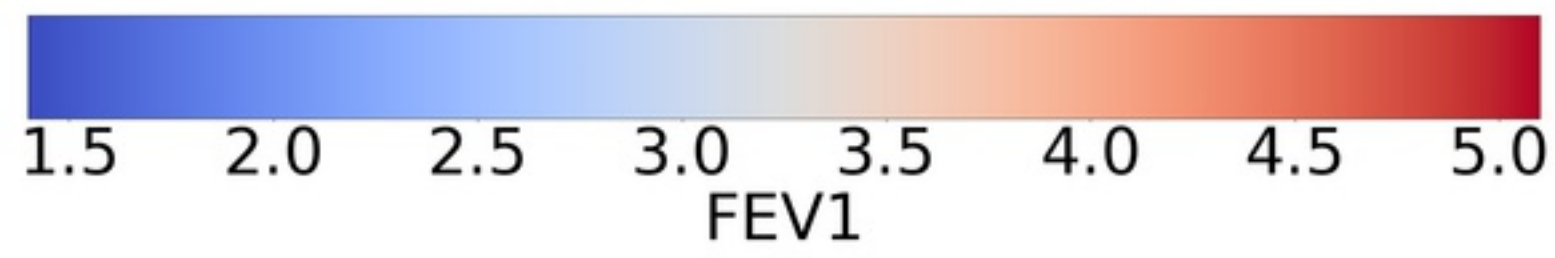

Supplement: S28 Fig — UMAP on the top 10 principal components of the UKBB coloured by FEV1 (male). Data has been randomized as explained in the materials and methods section. (PDF) [file pgen.1008432.s028.pdf]

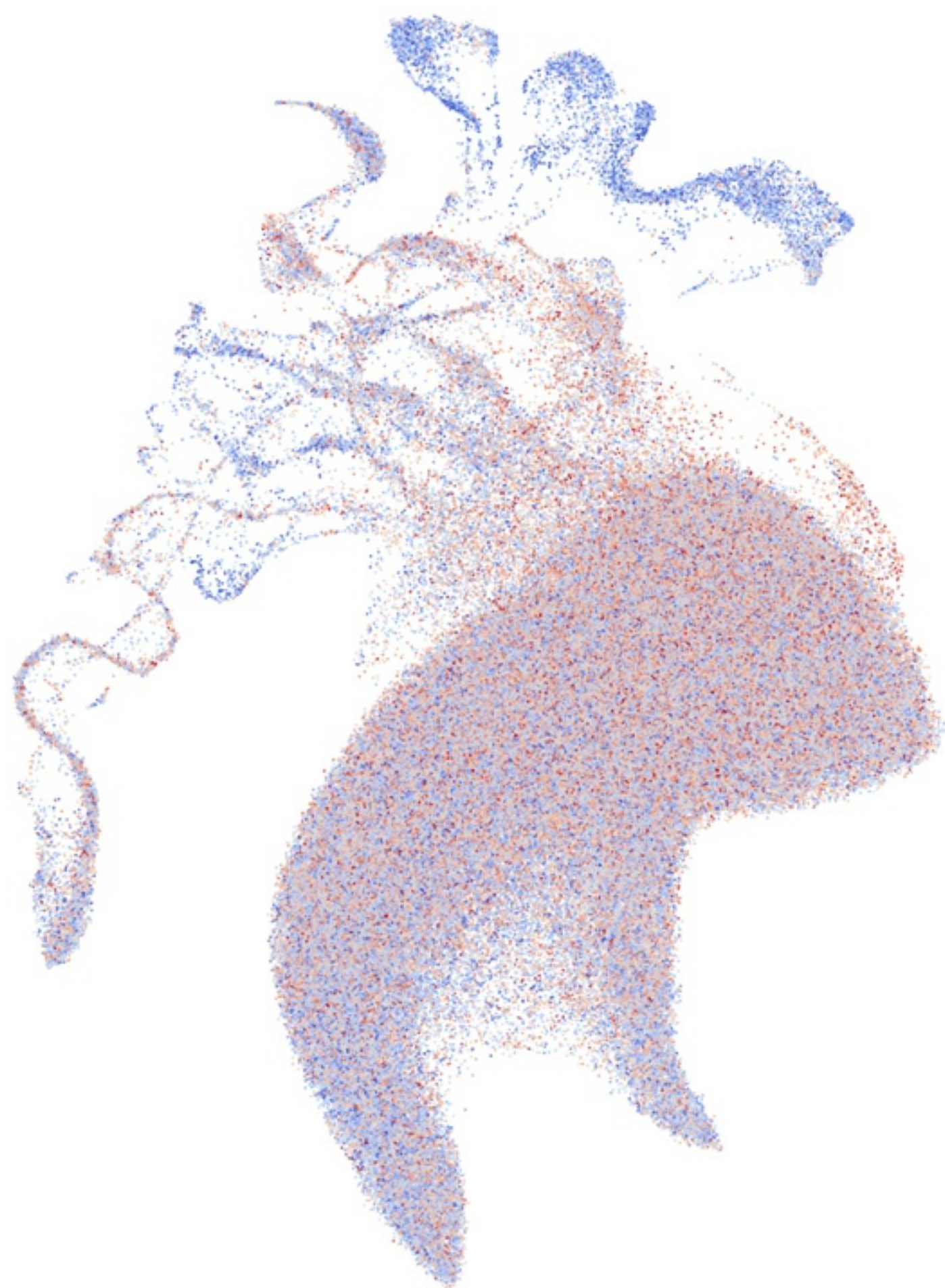

—10      —5      0      5      10  
Age-adjusted residual height (cm)

Supplement: S29 Fig — UMAP on the top 10 principal components of the UKBB coloured by height (female). Data has been randomized as explained in the materials and methods section. (PDF) [file pgen.1008432.s029.pdf]

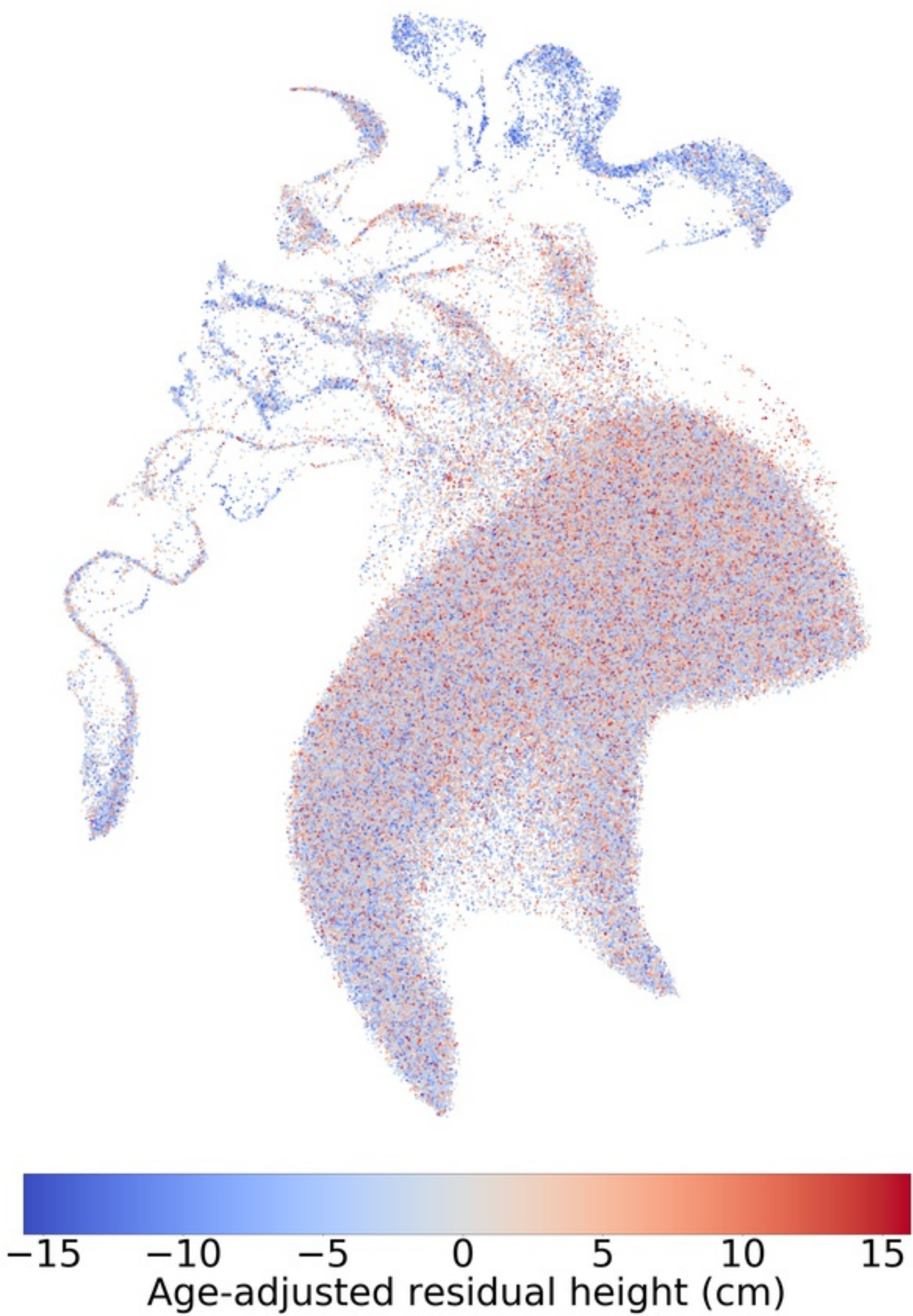

Supplement: S30 Fig — UMAP on the top 10 principal components of the UKBB coloured by height (male). Data has been randomized as explained in the materials and methods section. (PDF) [file pgen.1008432.s030.pdf]

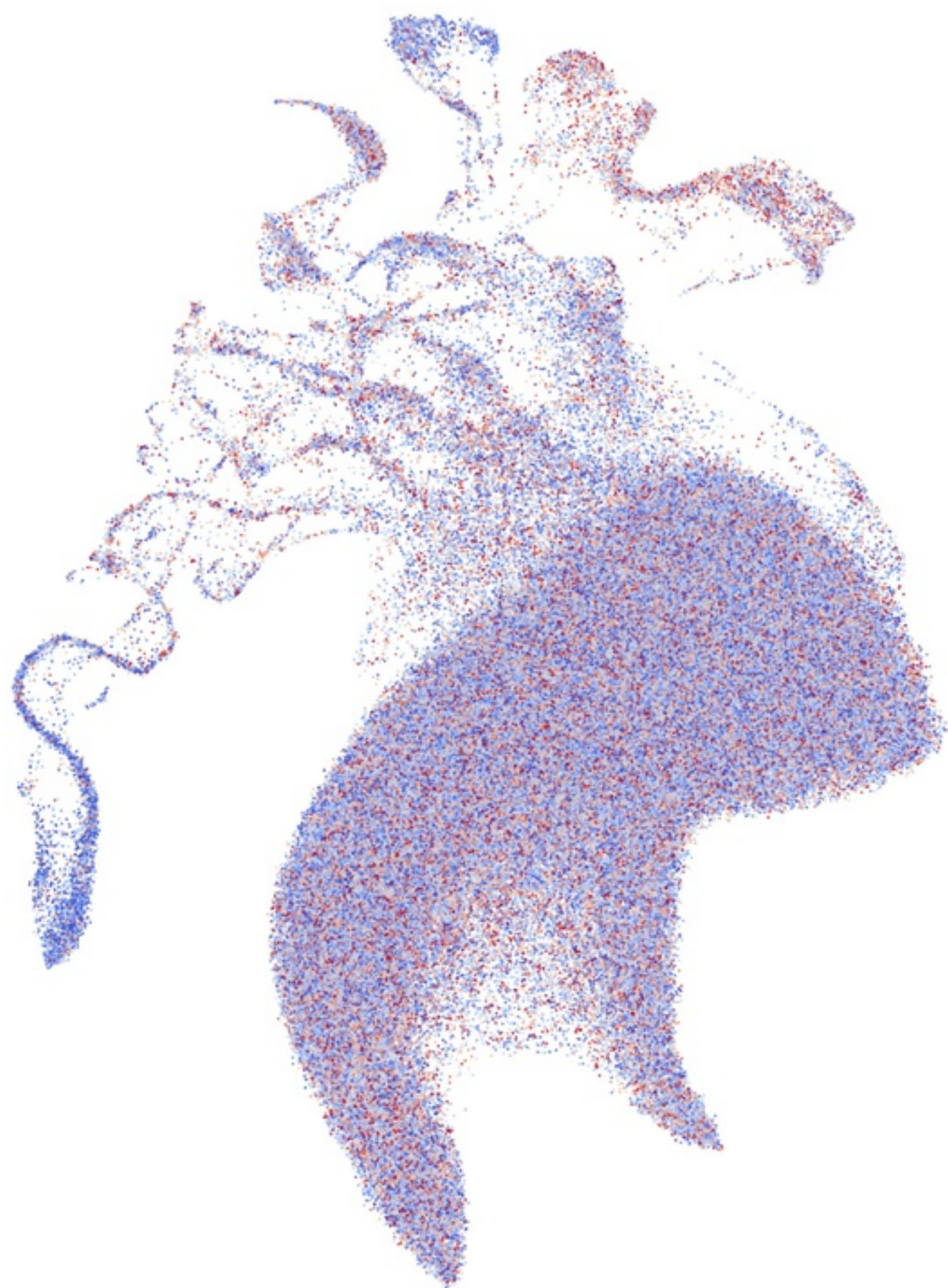

5

6

7

8

9

10

Leukocyte count ( $10^9$  cells/L)

Supplement: S31 Fig — UMAP on the top 10 principal components of the UKBB coloured by leukocyte count (female). Data has been randomized as explained in the materials and methods section. (PDF) [file pgen.1008432.s031.pdf]

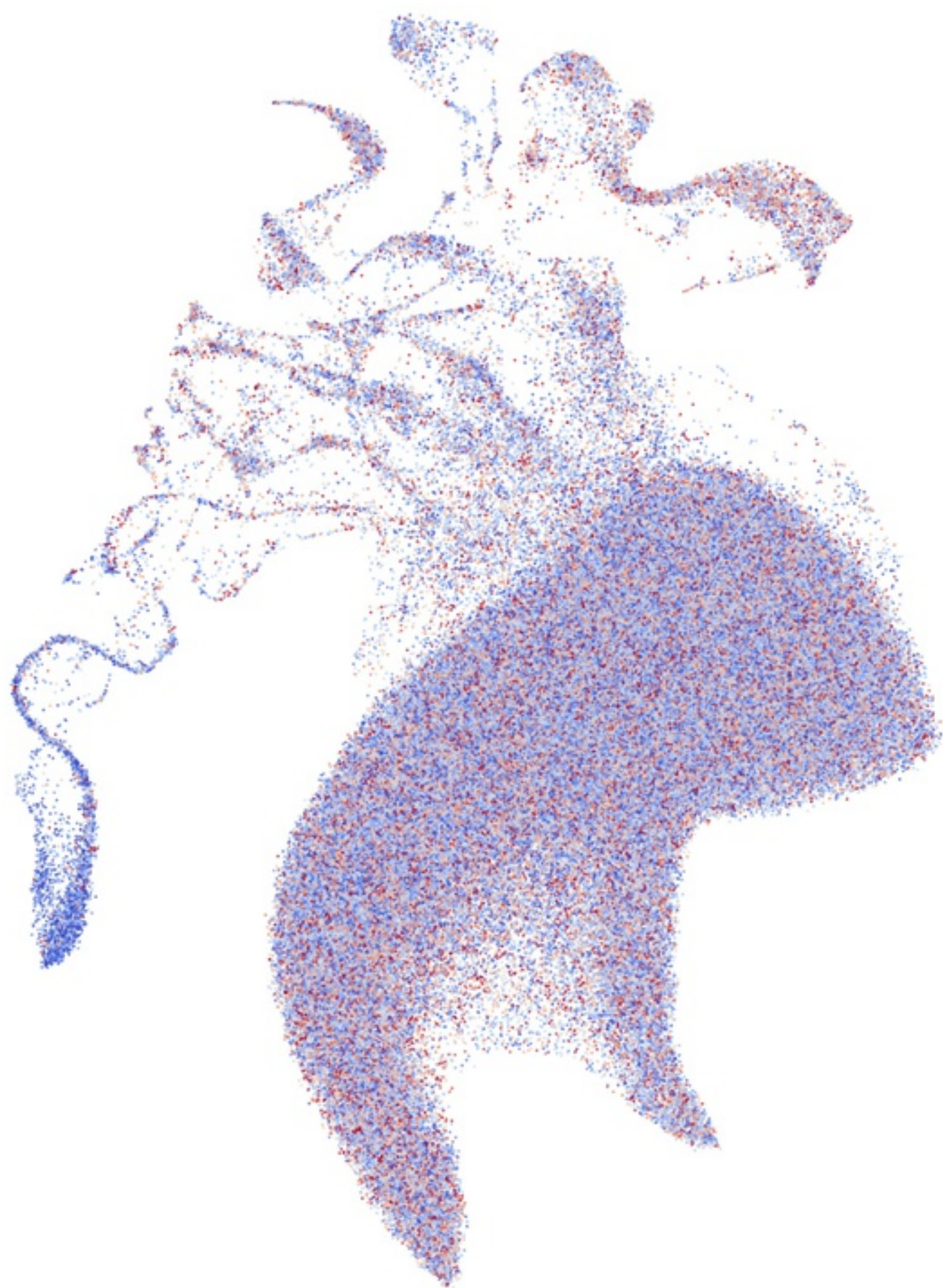

5

6

7

8

9

10

Leukocyte count (10<sup>9</sup> cells/L)

Supplement: S32 Fig — UMAP on the top 10 principal components of the UKBB coloured by leukocyte count (male). Data has been randomized as explained in the materials and methods section. (PDF) [file pgen.1008432.s032.pdf]

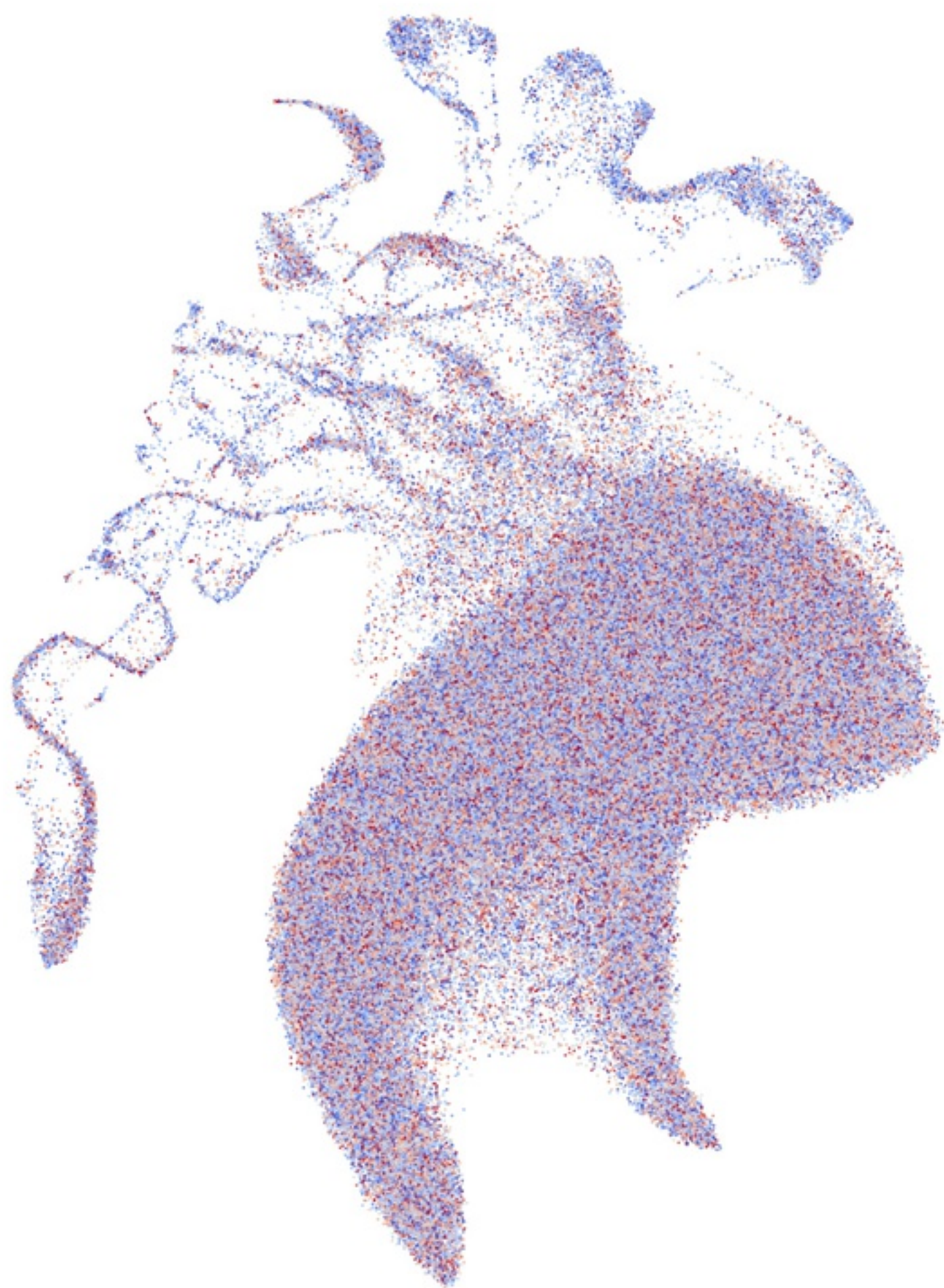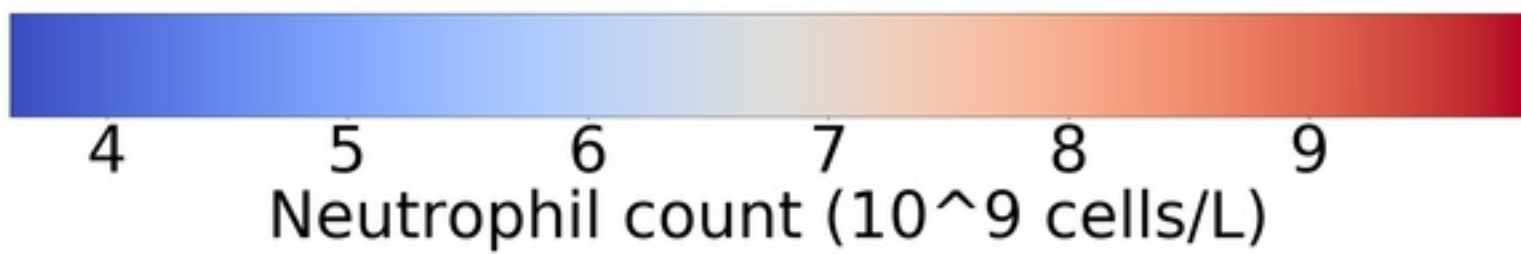

Supplement: S33 Fig — UMAP on the top 10 principal components of the UKBB coloured by neutrophil count (female). Data has been randomized as explained in the materials and methods section. (PDF) [file pgen.1008432.s033.pdf]

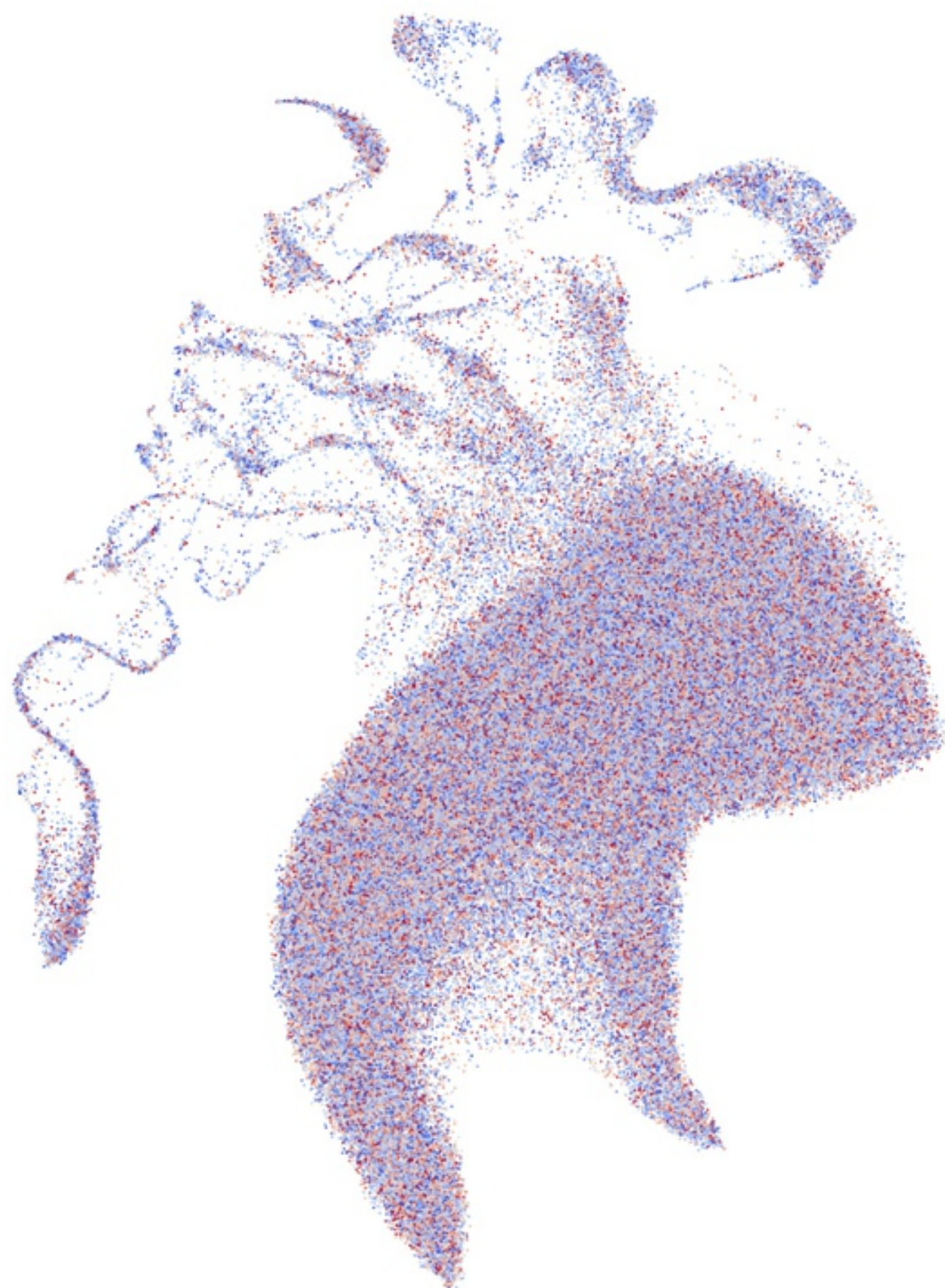

5

6

7

8

9

10

11

Neutrophil count ( $10^9$  cells/L)

Supplement: S34 Fig — UMAP on the top 10 principal components of the UKBB coloured by neutrophil count (male). Data has been randomized as explained in the materials and methods section. (PDF) [file pgen.1008432.s034.pdf]

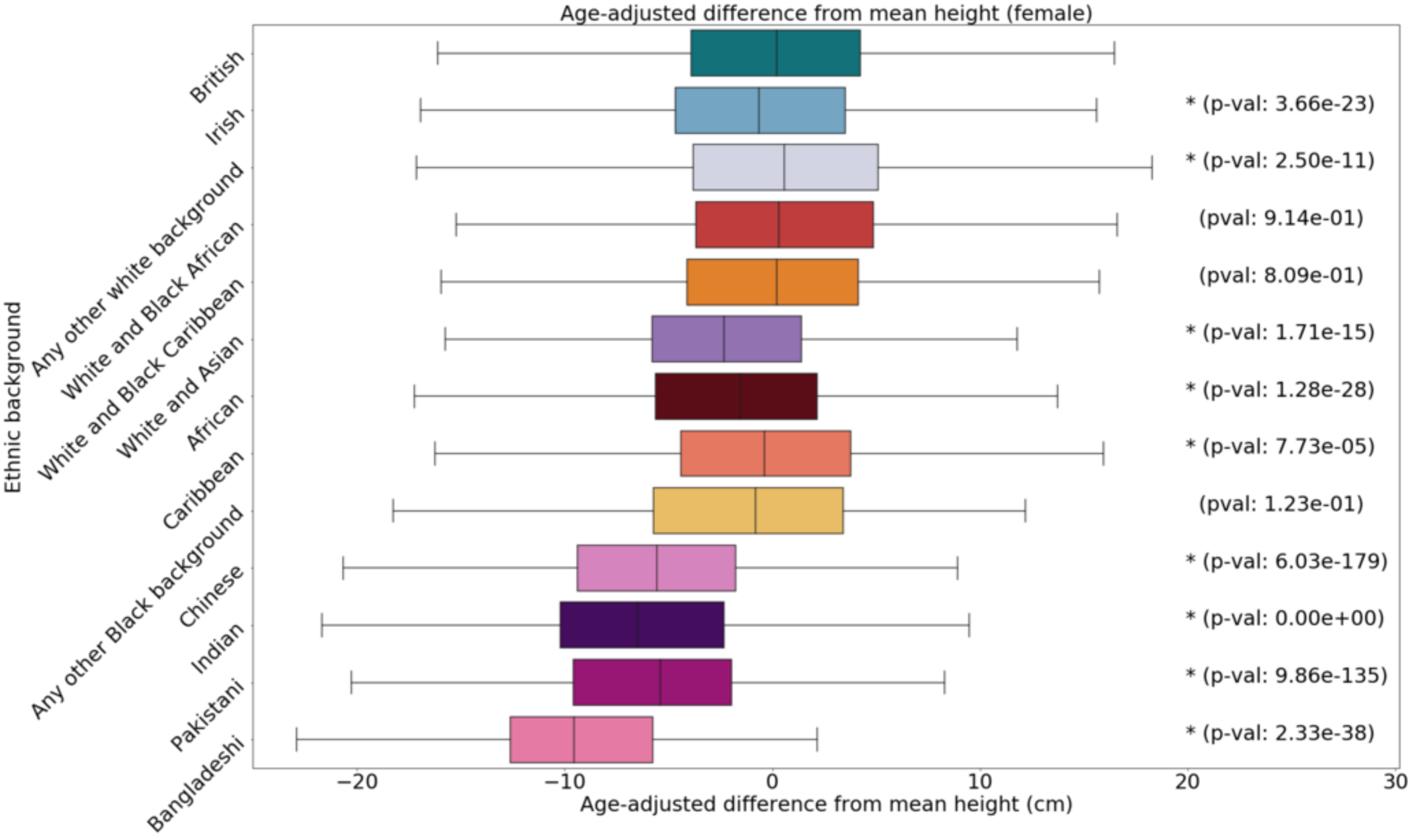

Supplement: S35 Fig — Height by sex and ethnic group, annotated with p-values. Asterisks indicate significant difference from the White British group with a Bonferroni correction for 12 groups. (PDF) [file pgen.1008432.s035.pdf]

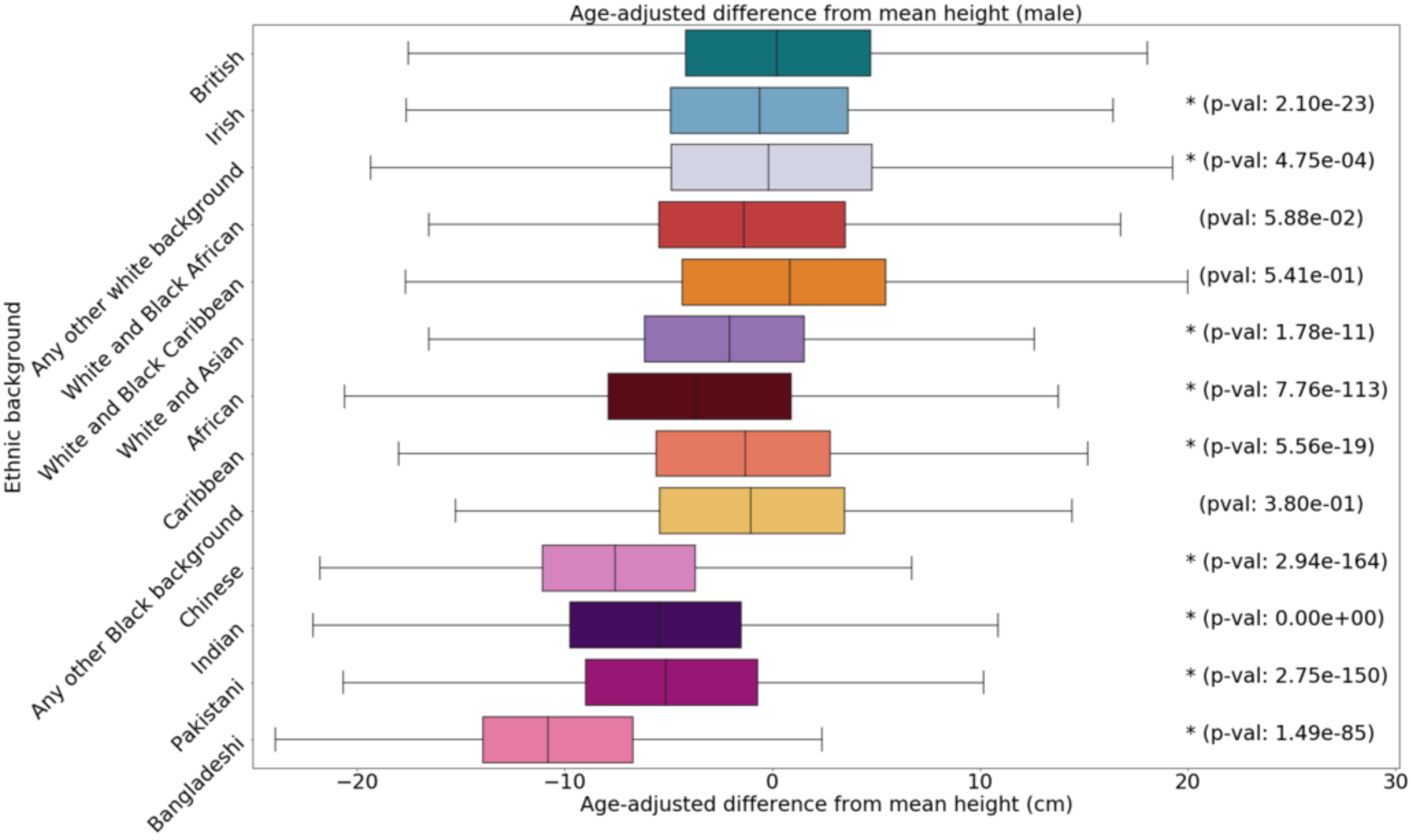

Supplement: S36 Fig — Height by sex and ethnic group, annotated with p-values. Asterisks indicate significant difference from the White British group with a Bonferroni correction for 12 groups. (PDF) [file pgen.1008432.s036.pdf]

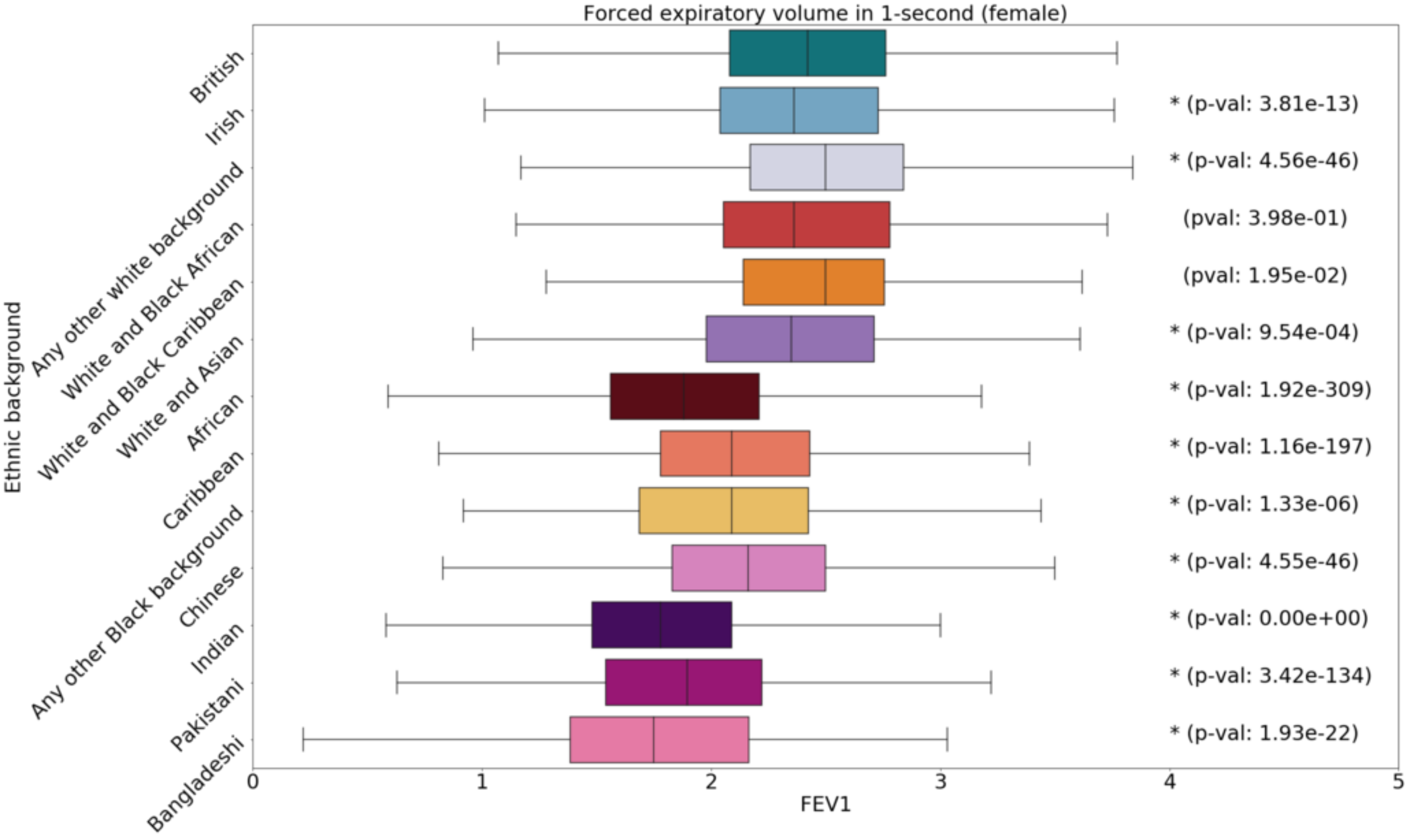

Supplement: S37 Fig — FEV1 by sex and ethnic group, annotated with p-values. Asterisks indicate significant difference from the White British group with a Bonferroni correction for 12 groups. (PDF) [file pgen.1008432.s037.pdf]

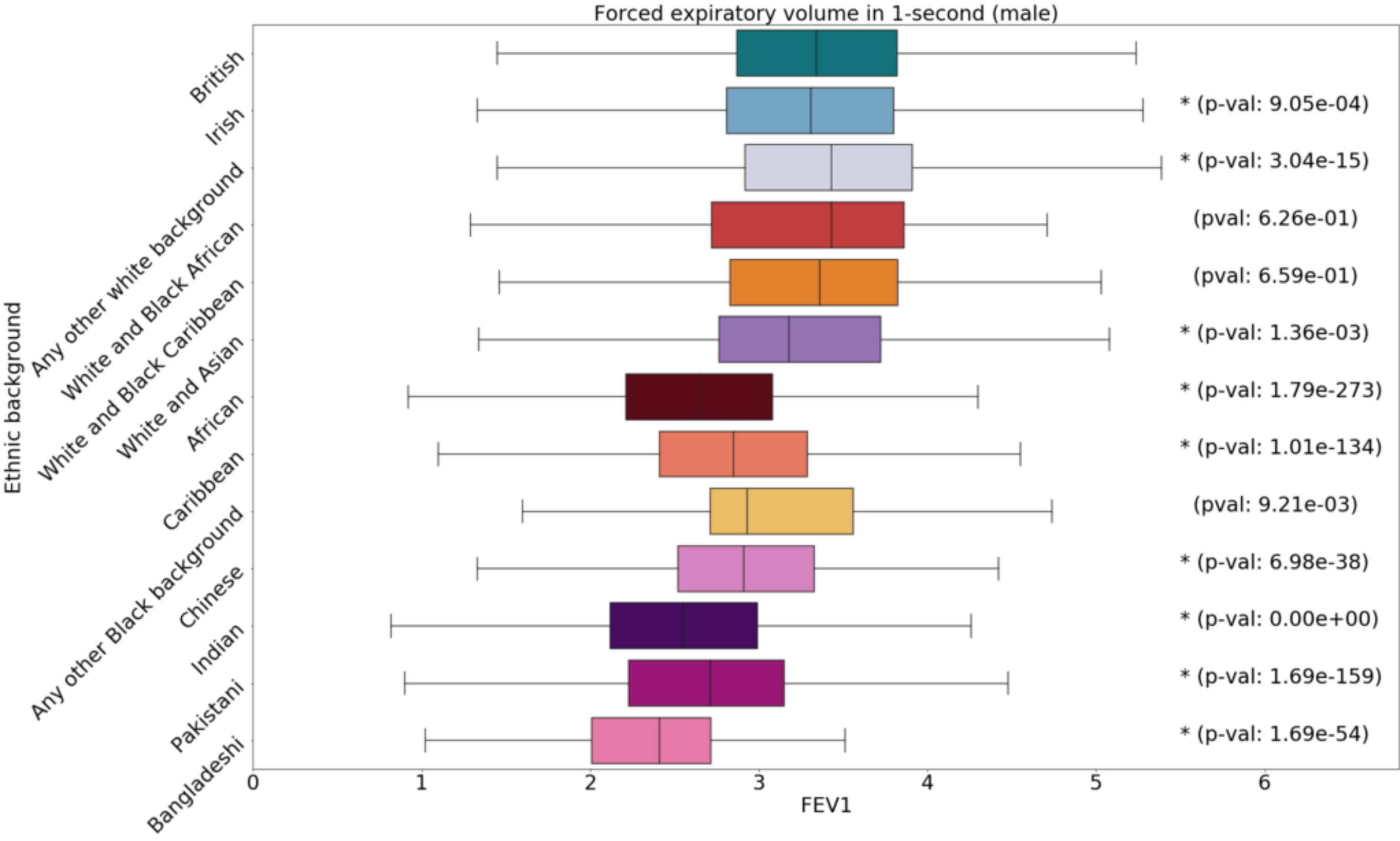

Supplement: S38 Fig — FEV1 by sex and ethnic group, annotated with p-values. Asterisks indicate significant difference from the White British group with a Bonferroni correction for 12 groups. (PDF) [file pgen.1008432.s038.pdf]

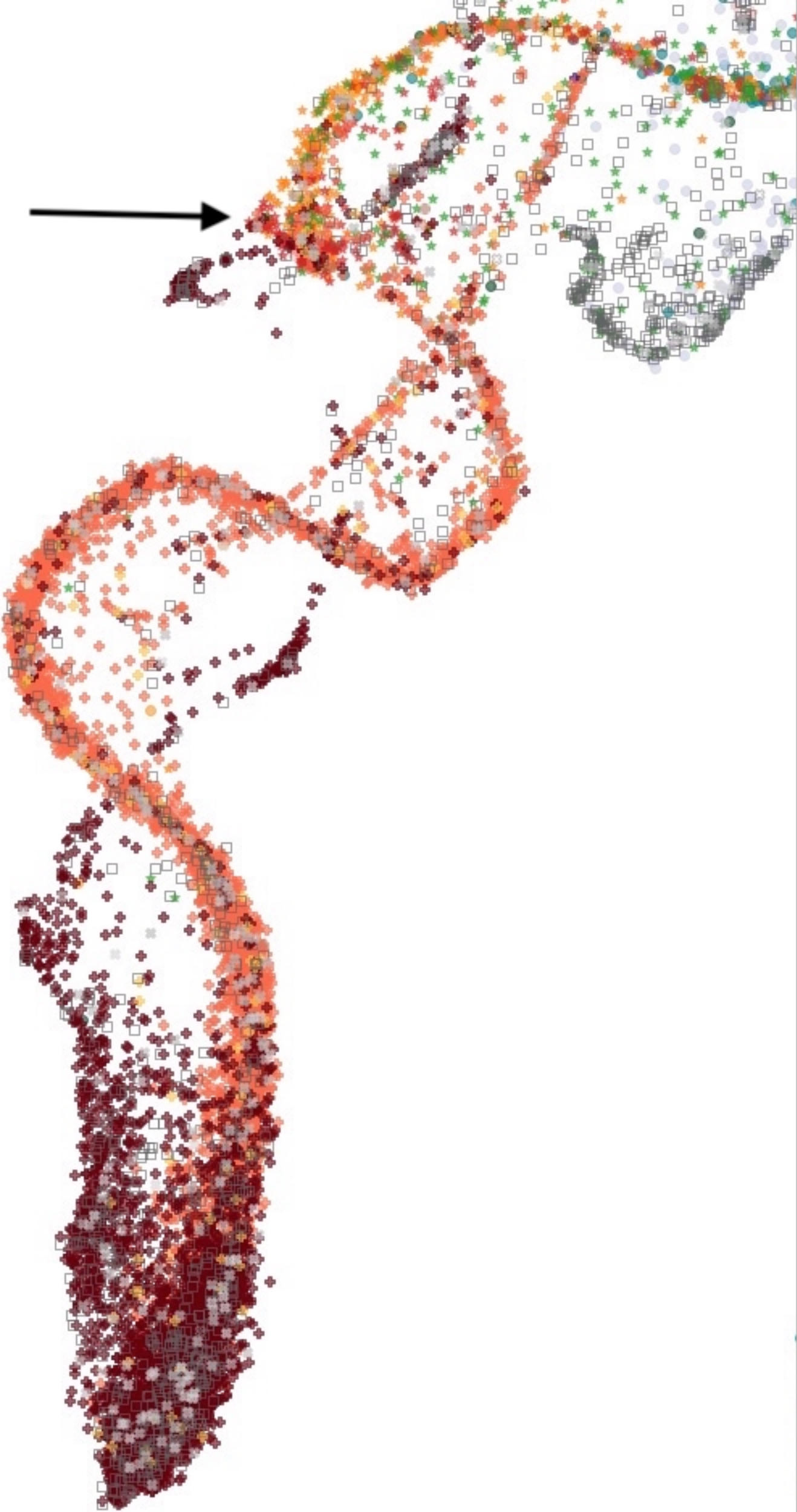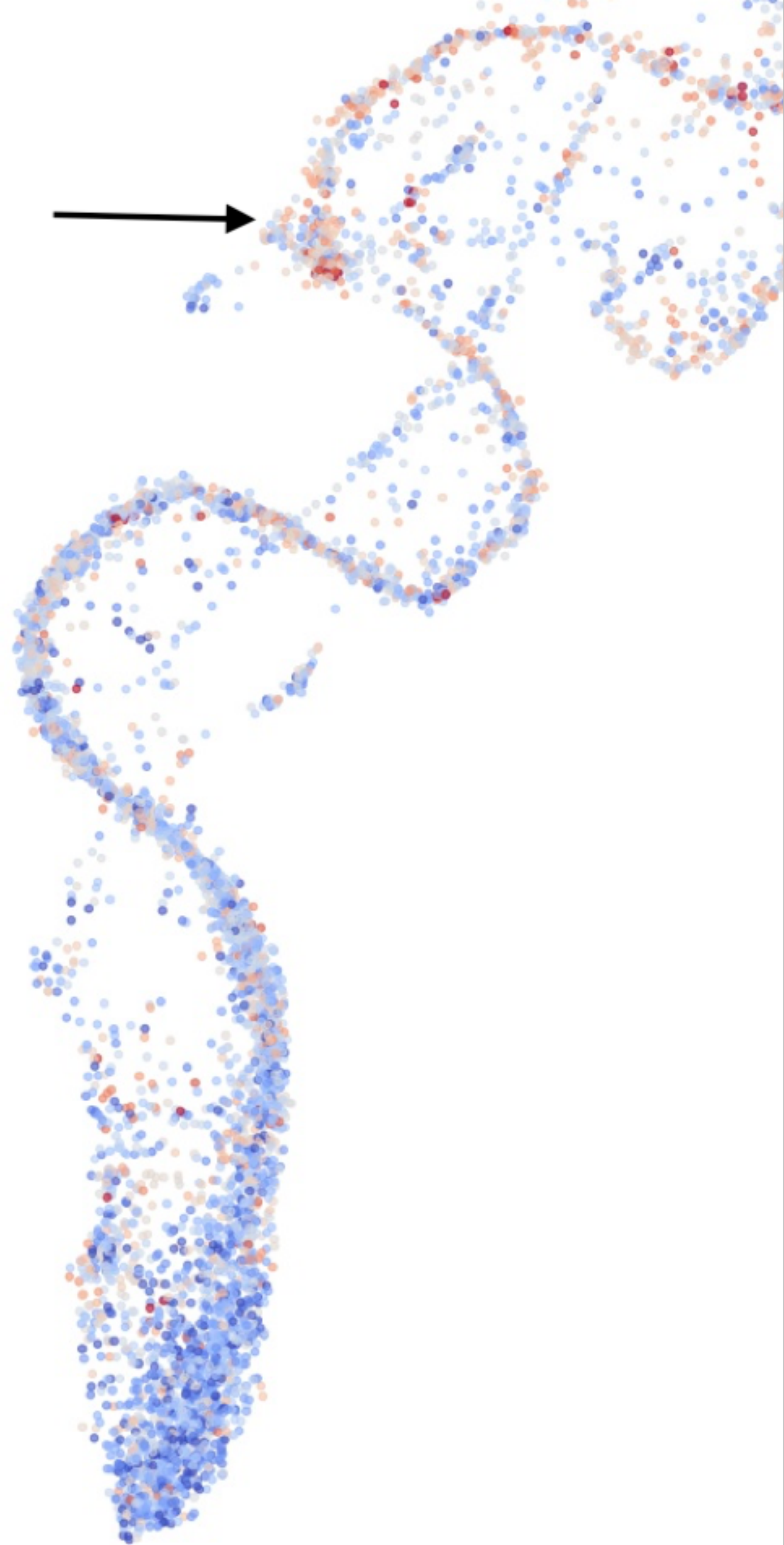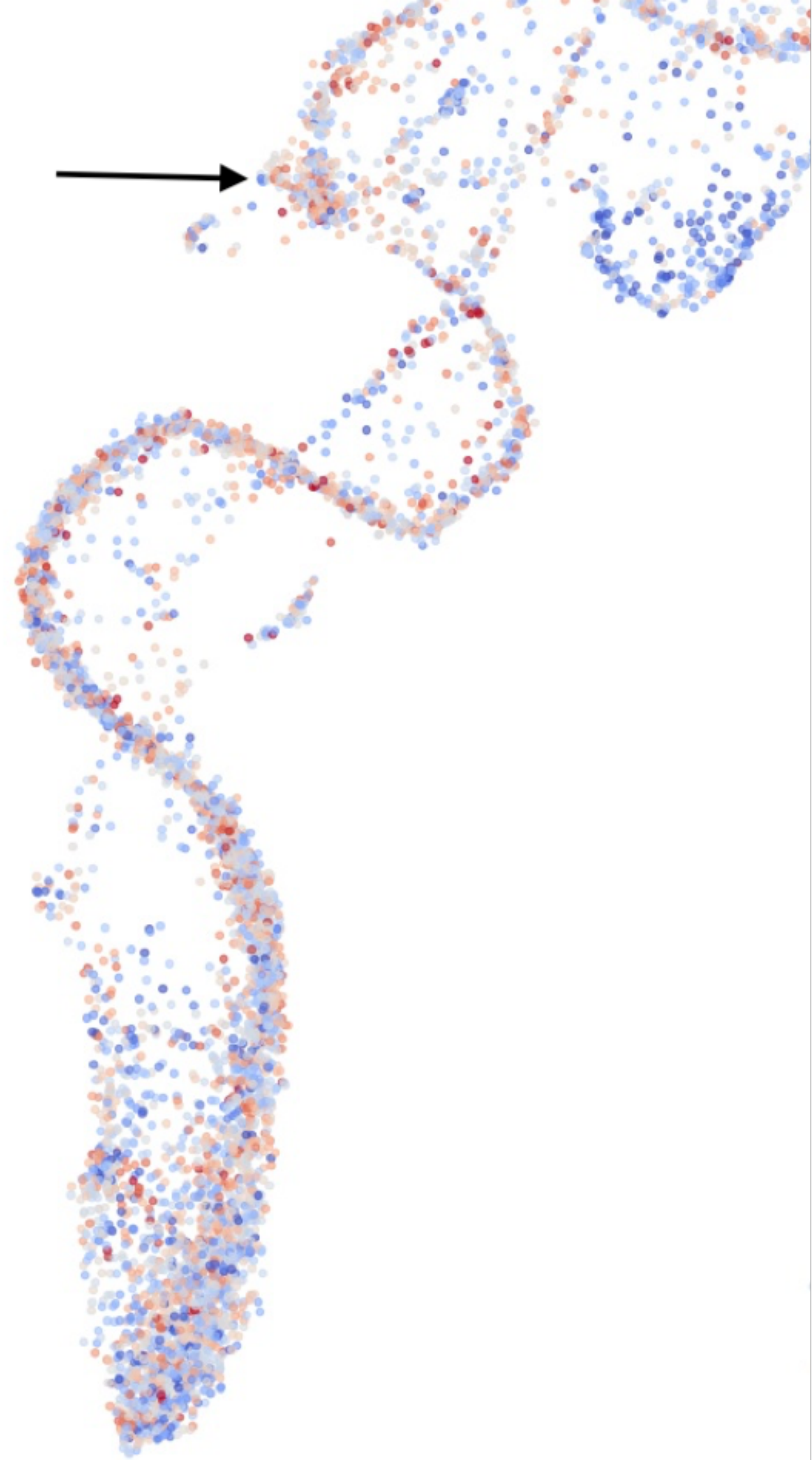

Supplement: S39 Fig — Individuals of Black African, Black Caribbean, and mixed backgrounds (primarily White and Black Caribbean/African) coloured by self-identified ethnic background (left, from Fig 3B), FEV1 (middle), and age-adjusted height (right). An arrow points to an area where the FEV1 distribution appears to change, corresponding to where the clusters contain more people with self-identified mixed backgrounds. (PDF) [file pgen.1008432.s039.pdf]

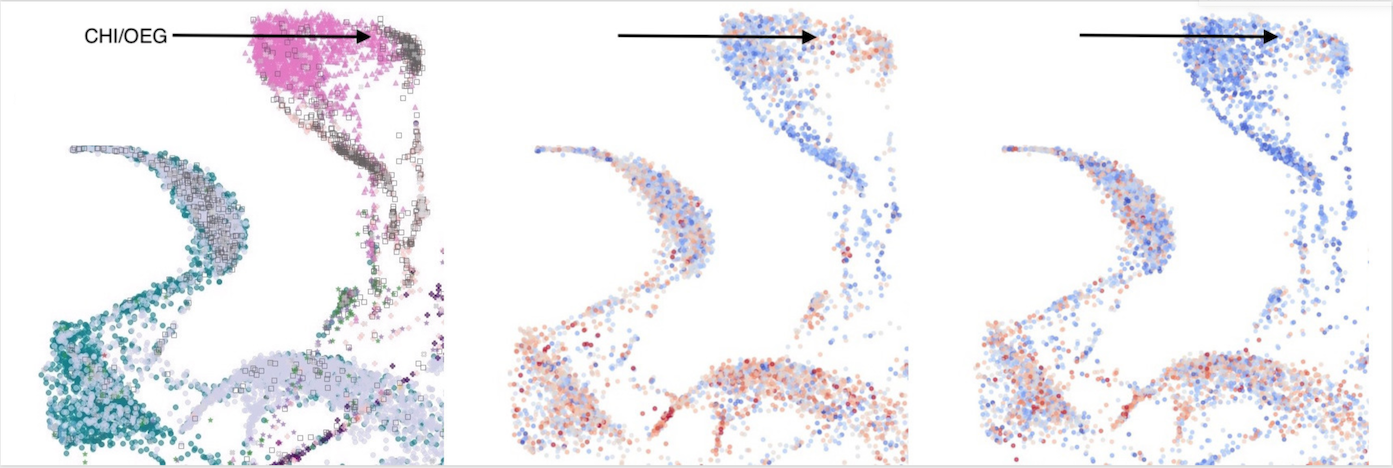

Supplement: S40 Fig — Zoomed in section of Fig 3B, focused on individuals with Chinese (CHI), White British (GBR), any other white background, or any other ethnic group (OEG) coloured by ethnicity (left), FEV1 (middle), and age-adjusted height (right). The OEG cluster next to the Chinese cluster appears redder on the middle panel, suggesting higher levels of FEV1. (PNG) [file pgen.1008432.s040.png]

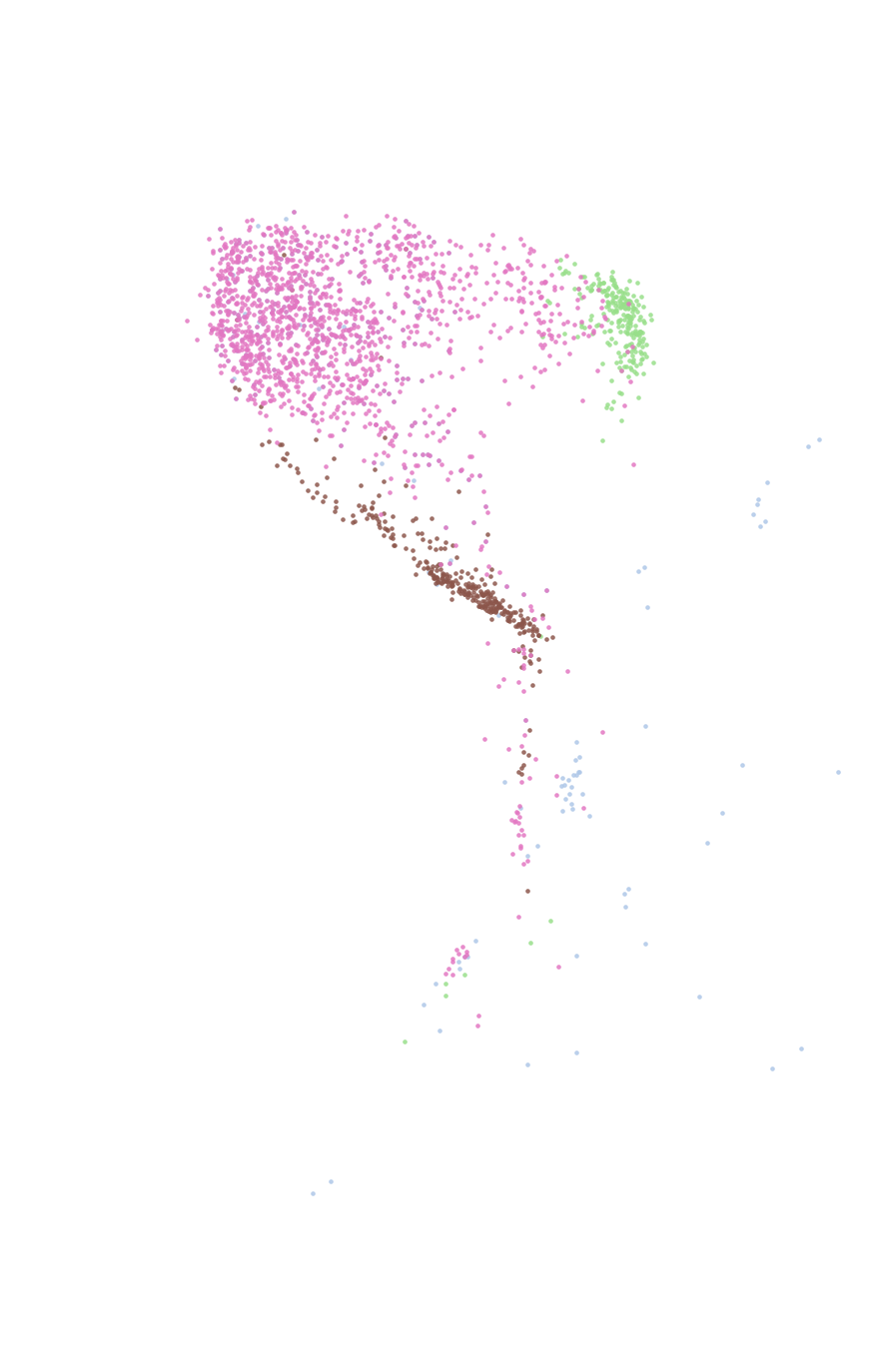

Supplement: S41 Fig — Individuals from the zoomed in section in S40 Fig used in statistical testing, coloured the same as in S42 Fig. Brown, blue, and green represent those born in the Philippines, Malaysia, and Japan; pink represents those who self-identify as Chinese. The Chinese individuals were those who self-identified their ethnic background as Chinese, and the remaining populations were determined based on country of birth; the categorizations are mutually exclusive. (PNG) [file pgen.1008432.s041.png]

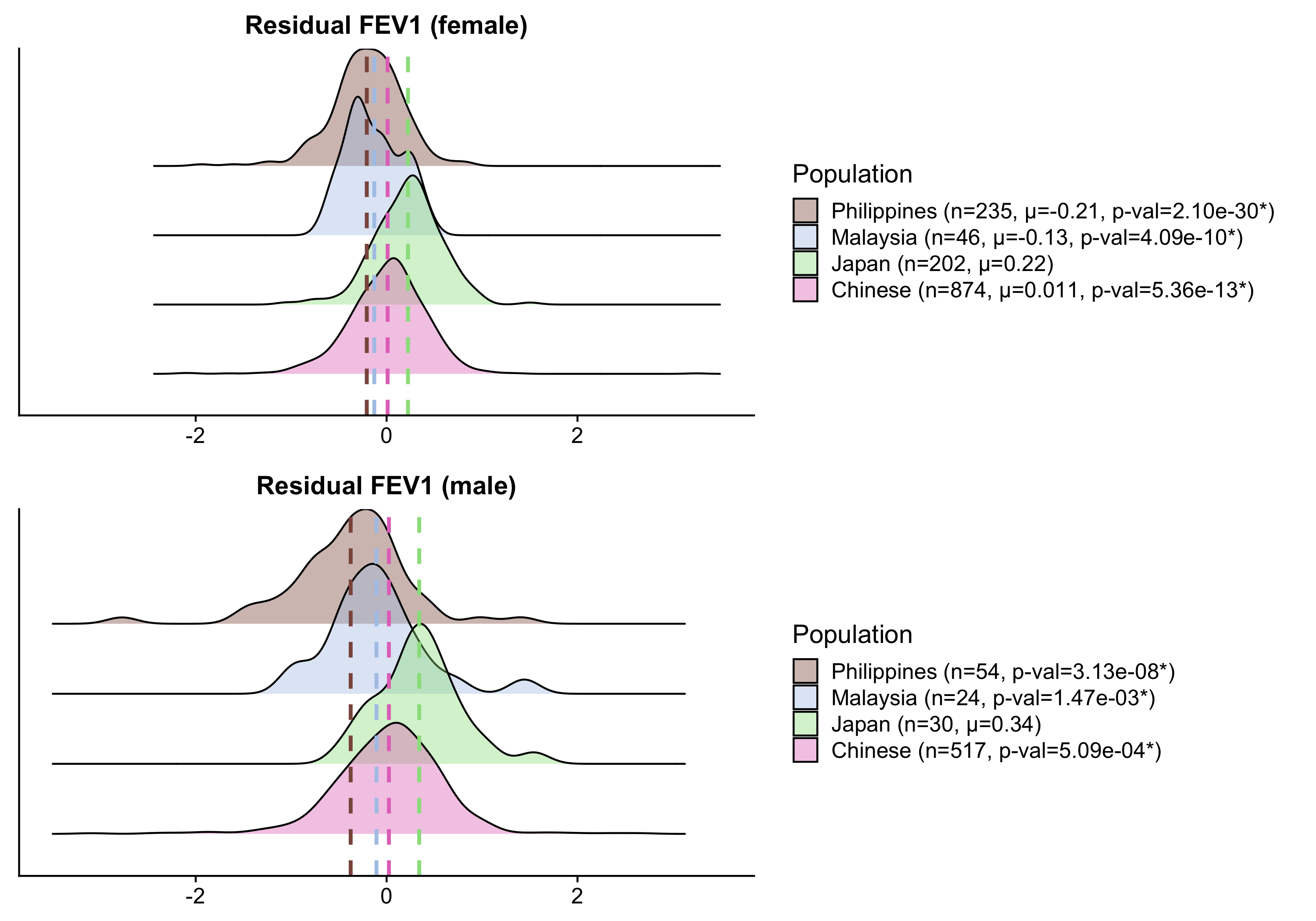

Supplement: S42 Fig — Plots of the distributions of residual FEV1 by sex for East Asian populations, after adjusting for height, age, age2, and sex through linear regression. Individuals were limited to those in the “Chinese/Other Ethnic Group” cluster from S40 Fig. The Chinese individuals were those who self-identified their ethnic background as Chinese, and the remaining populations were determined based on country of birth; the categorizations are mutually exclusive. Asterisks indicate significant difference from the Japanese population, using Welch’s unpaired t-test with a Bonferroni correction for 3 groups. The dashed lines are the means of the distributions, and Japanese populations have consistently higher means. (PNG) [file pgen.1008432.s042.png]

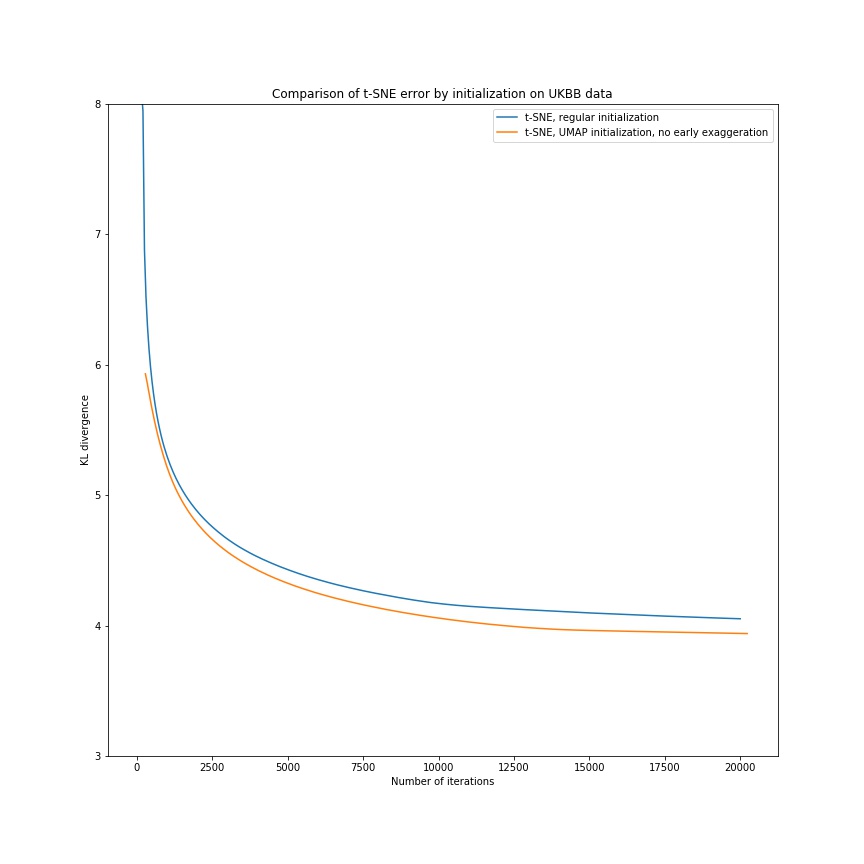

Supplement: S43 Fig — Comparing the error terms of standard t-SNE versus t-SNE initialized with a UMAP embedding and no early exaggeration. Done on the UKBB dataset with 20000 iterations. The UMAP-initialized graph has been shifted by 230 iterations to approximate the 230 epochs UMAP uses for large datasets (n > 10, 000). (JPEG) [file pgen.1008432.s043.jpeg]

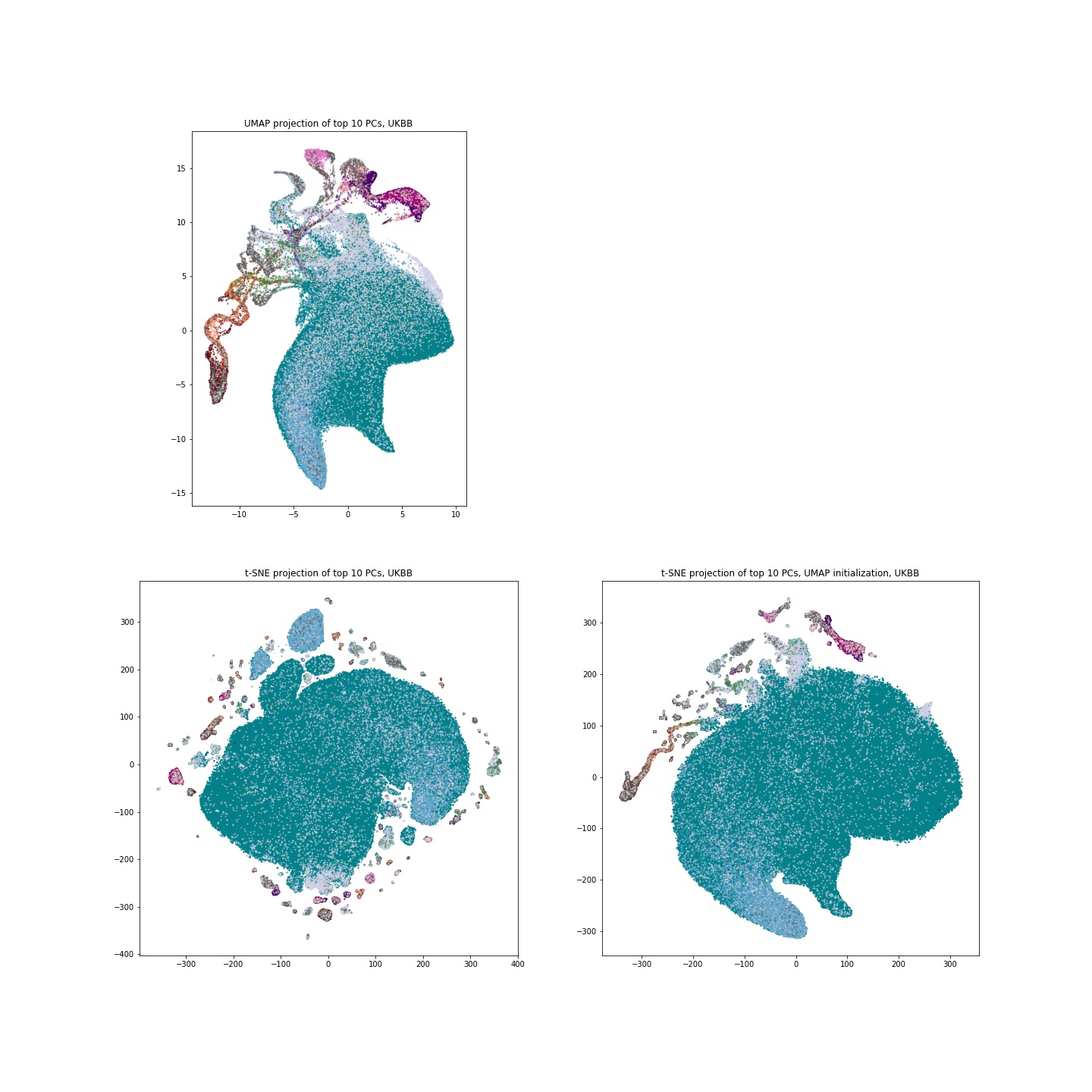

Supplement: S44 Fig — Comparing the visualizations of UMAP, standard t-SNE, and t-SNE initialized with a UMAP projection, on the top 10 principal components of the UKBB. t-SNE used 20000 iterations. (JPEG) [file pgen.1008432.s044.jpeg]

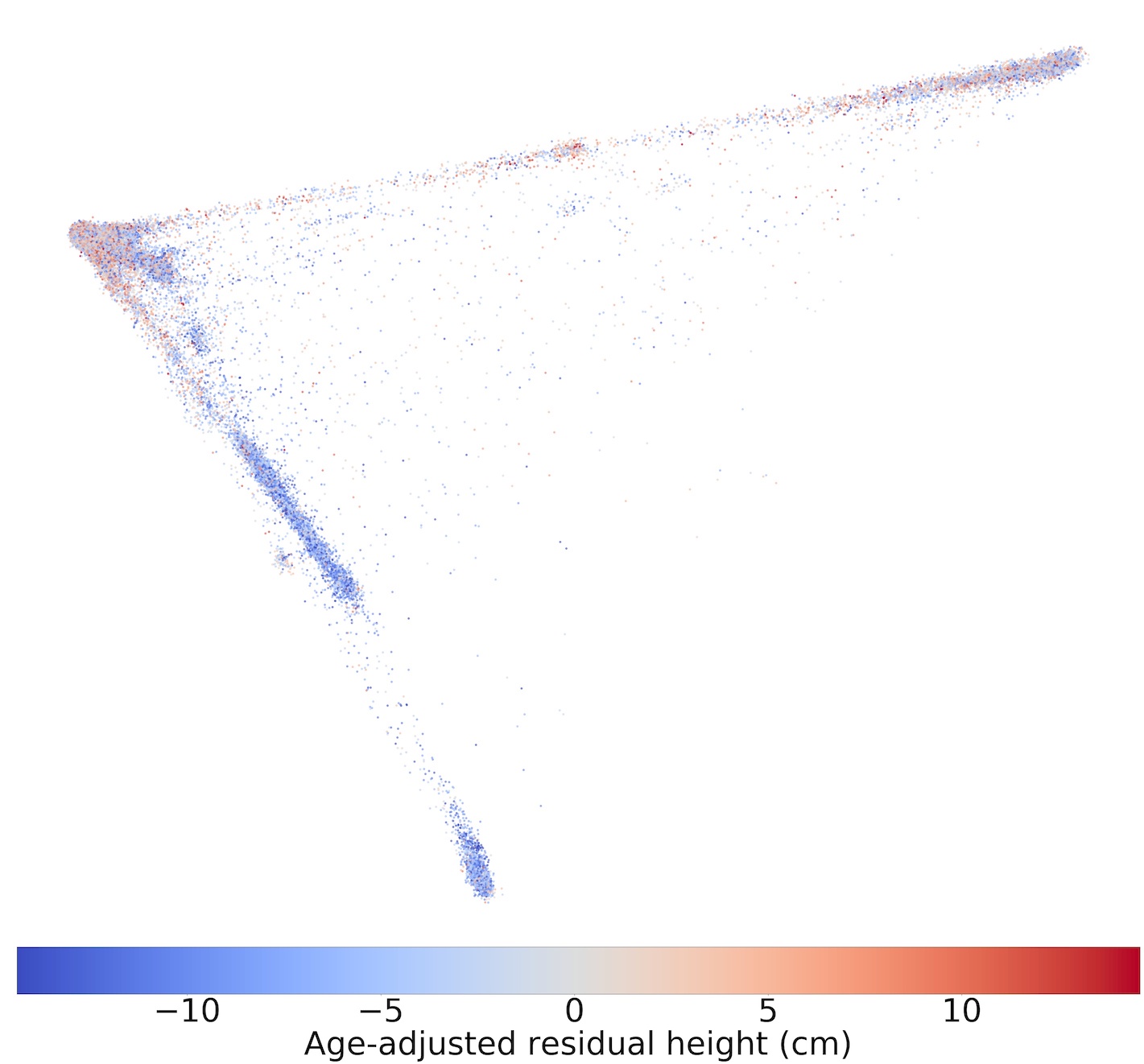

Supplement: S45 Fig — Principal components 1 and 2 from the UKBB, coloured by age-adjusted residual height (female). Data has been randomized as explained in the materials and methods section. (JPEG) [file pgen.1008432.s045.jpeg]

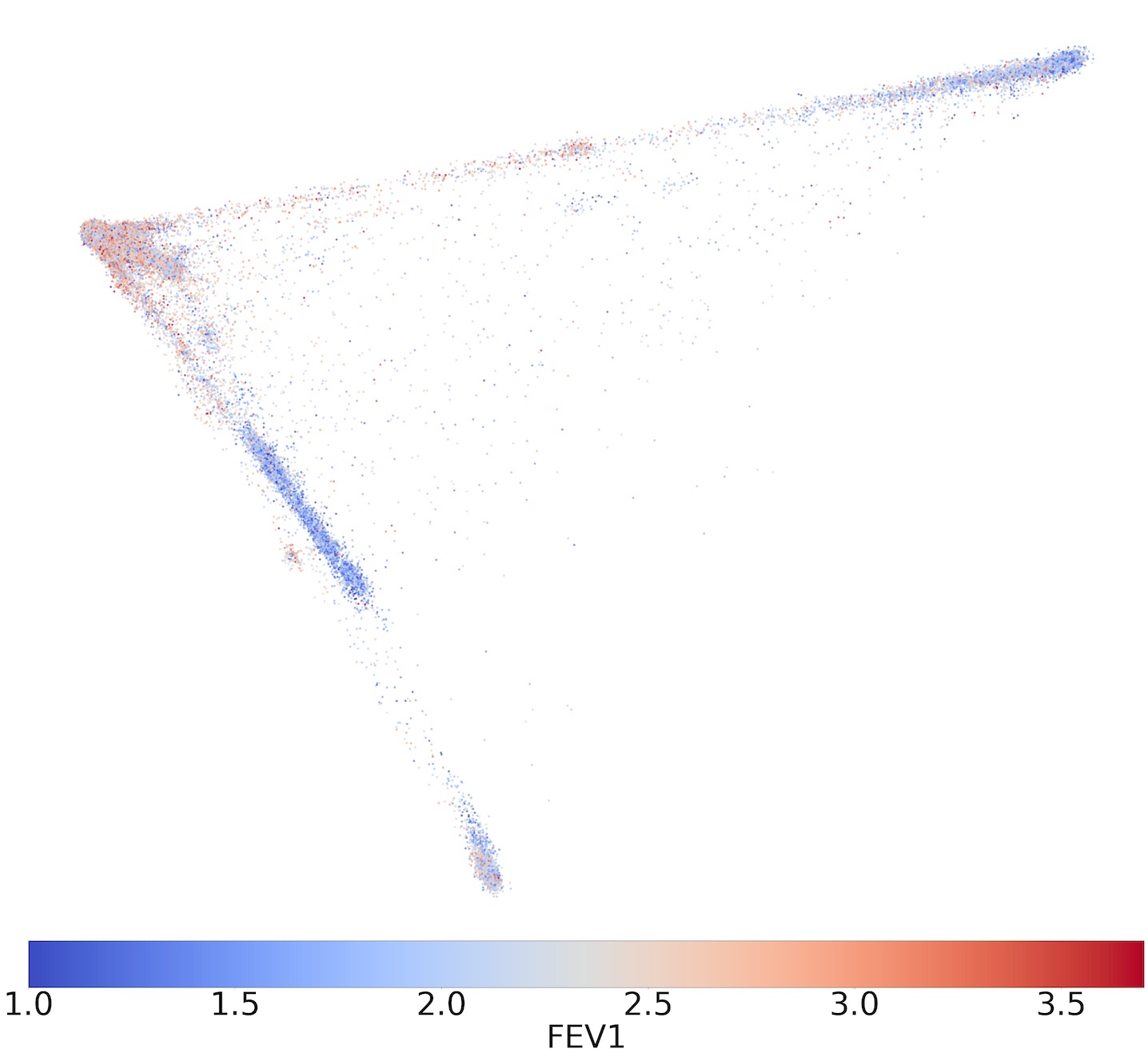

Supplement: S46 Fig — Principal components 1 and 2 from the UKBB, coloured by FEV1 (female). Data has been randomized as explained in the materials and methods section. (JPEG) [file pgen.1008432.s046.jpeg]

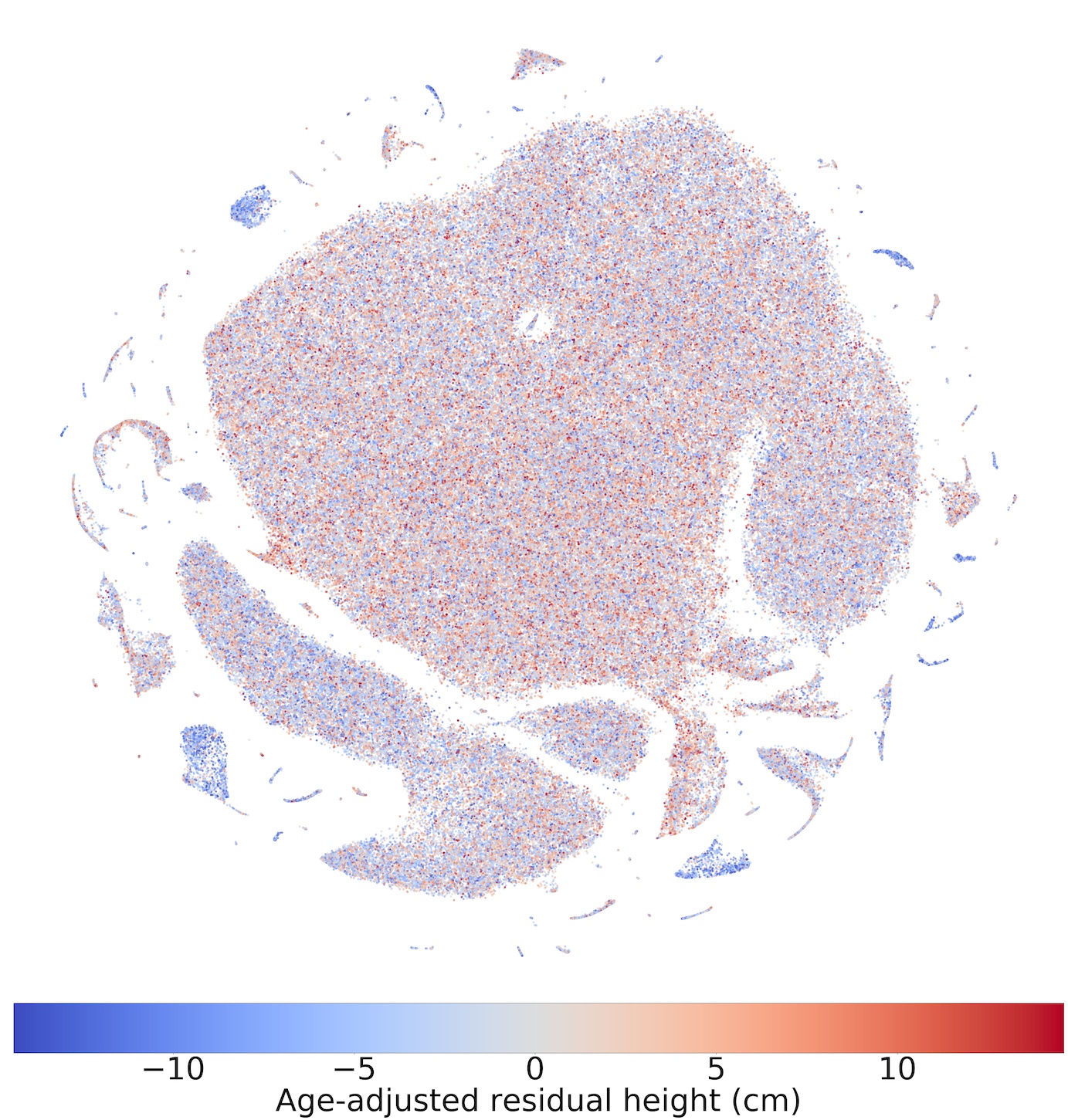

Supplement: S47 Fig — t-SNE on the first 10 principal components from the UKBB, coloured by age-adjusted residual height (female). Data has been randomized as explained in the materials and methods section. (JPEG) [file pgen.1008432.s047.jpeg]

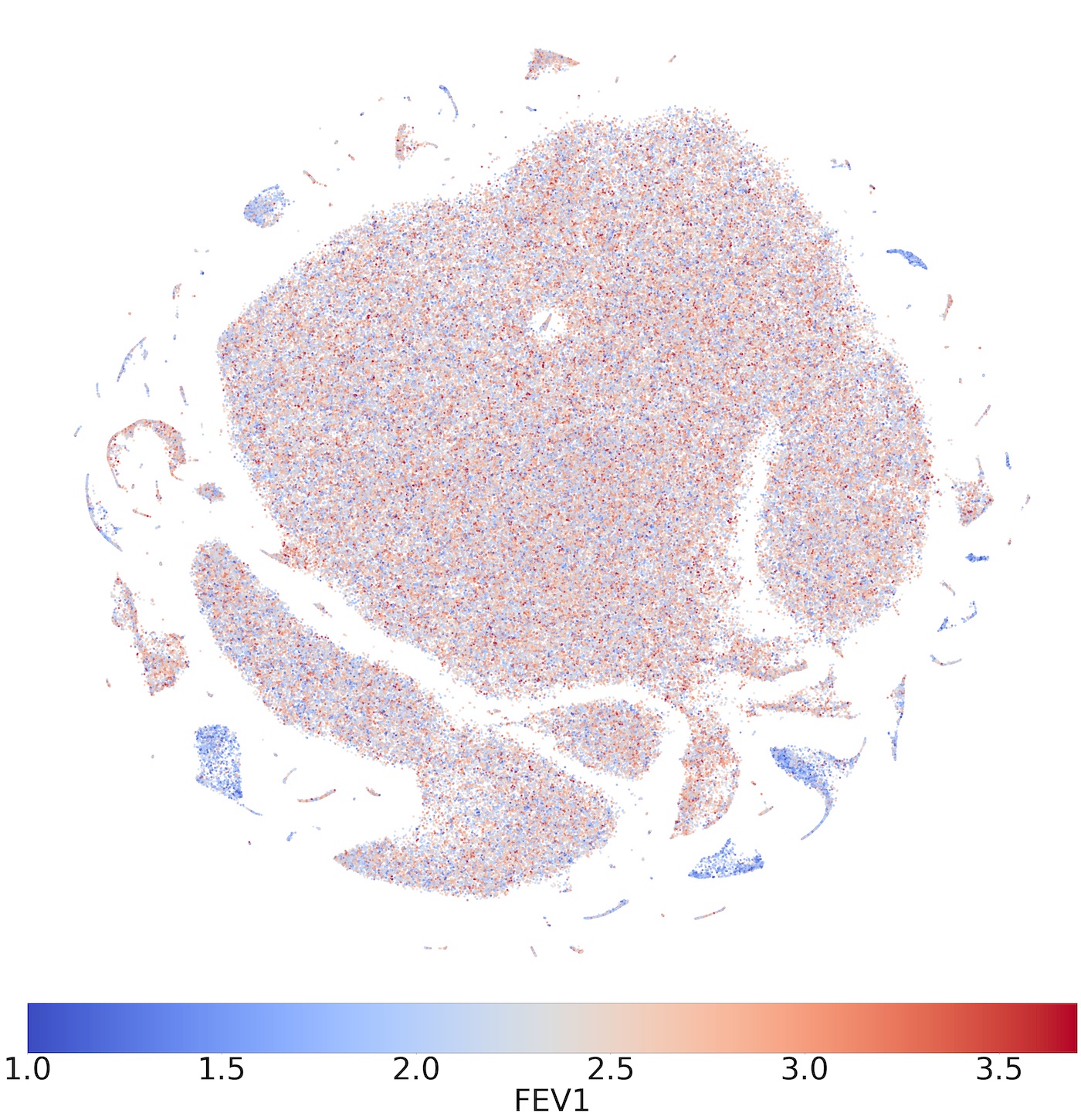

Supplement: S48 Fig — t-SNE on the first 10 principal components from the UKBB, coloured by FEV1 (female). Data has been randomized as explained in the materials and methods section. (JPEG) [file pgen.1008432.s048.jpeg]

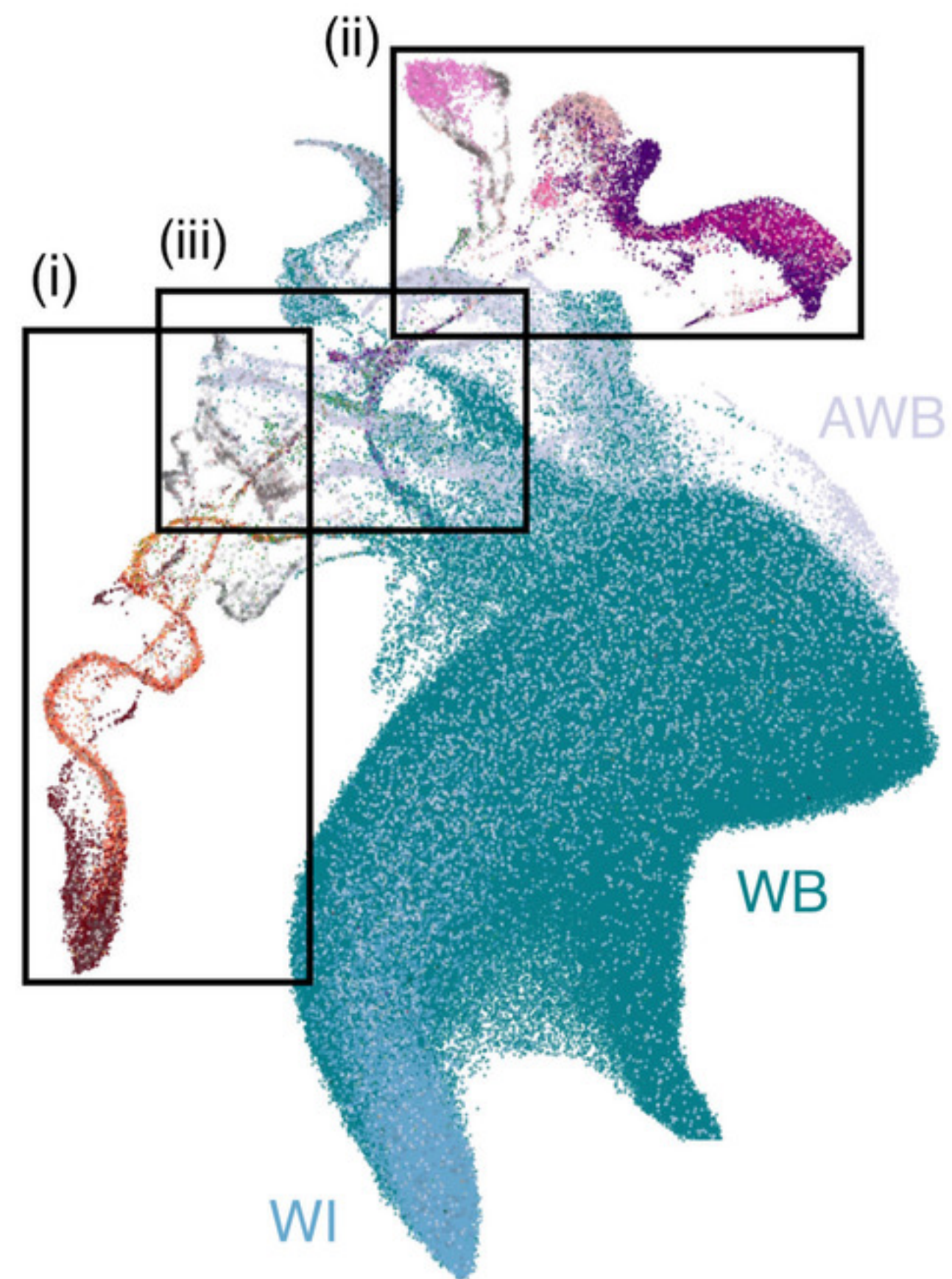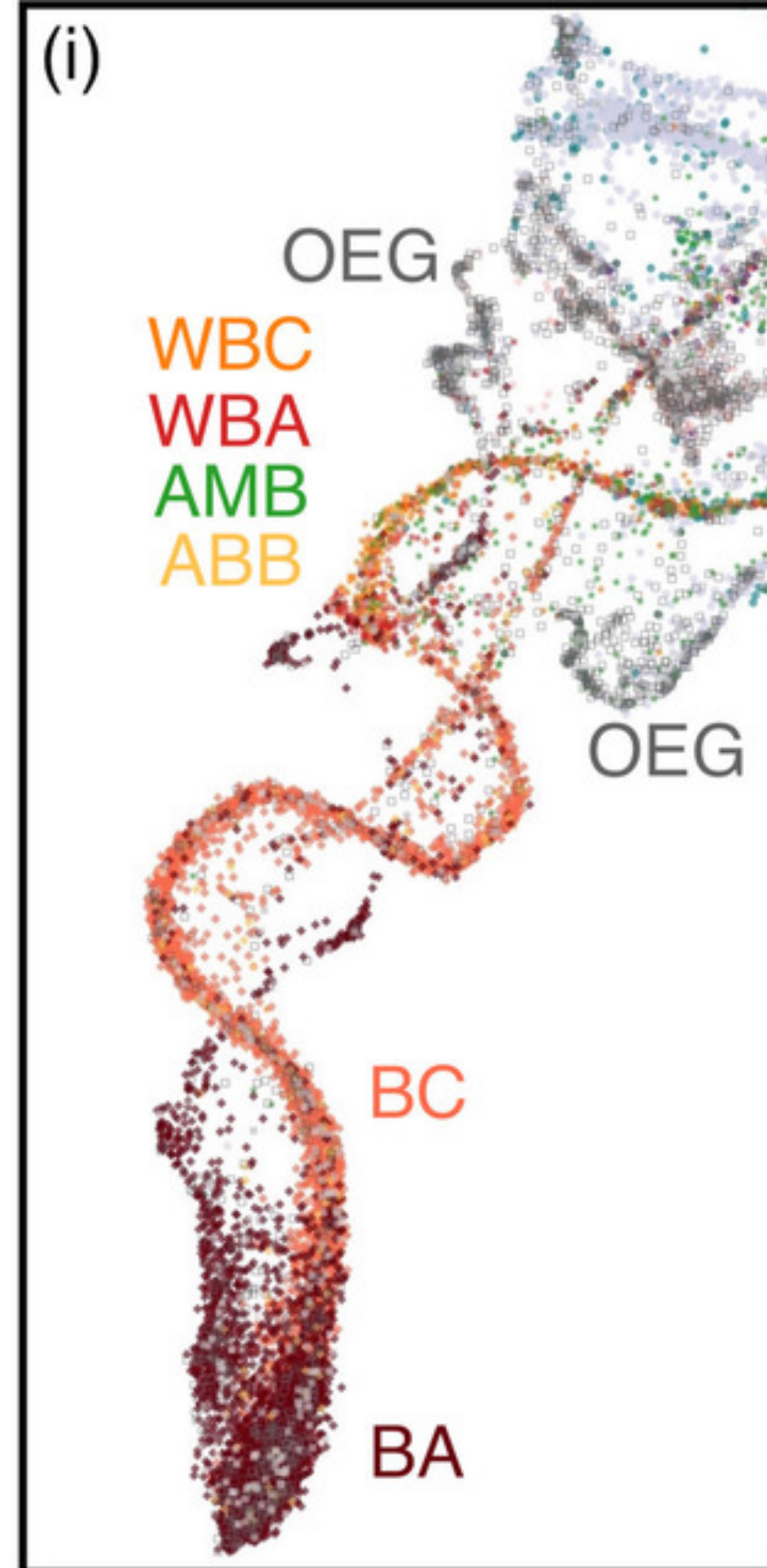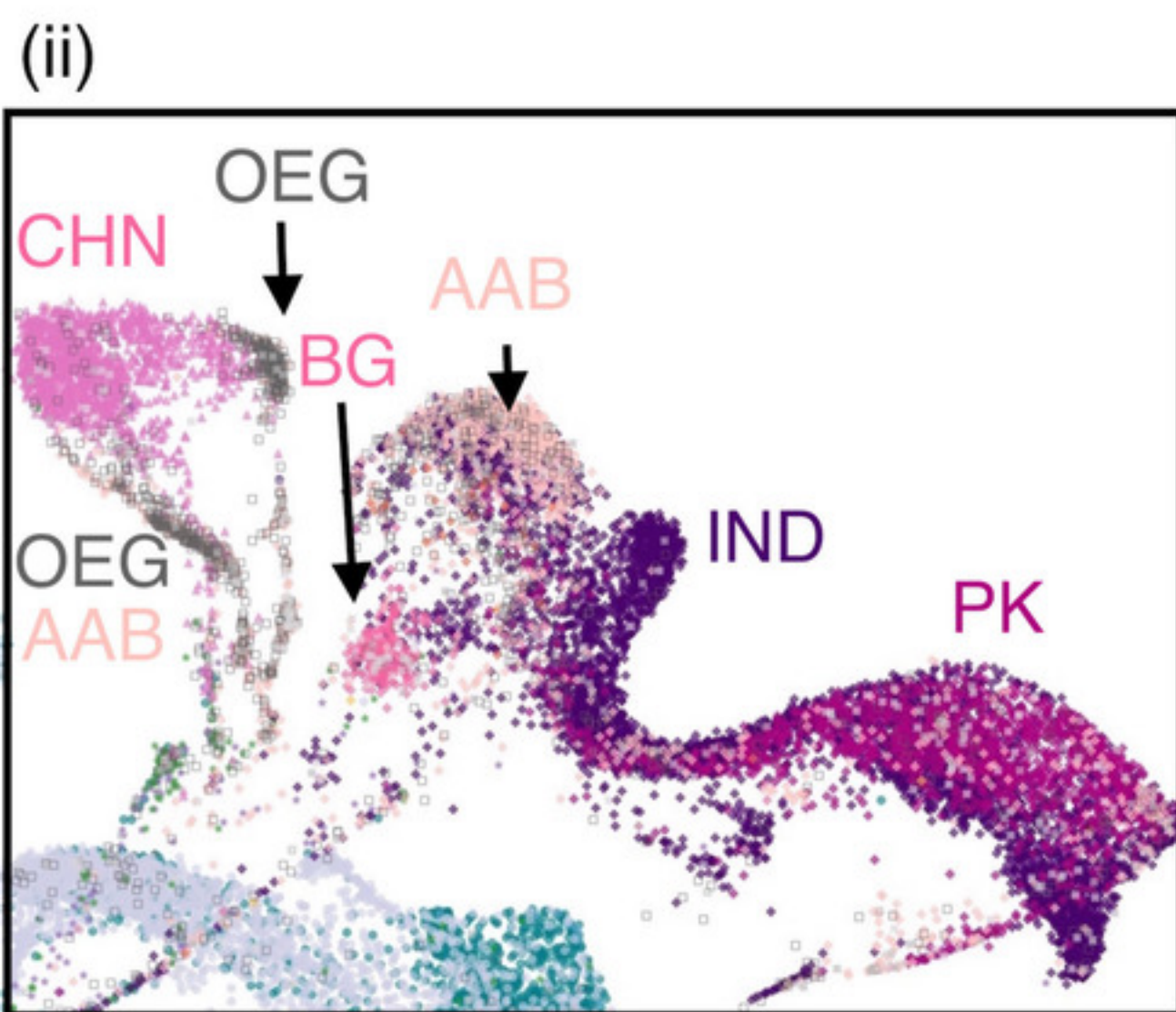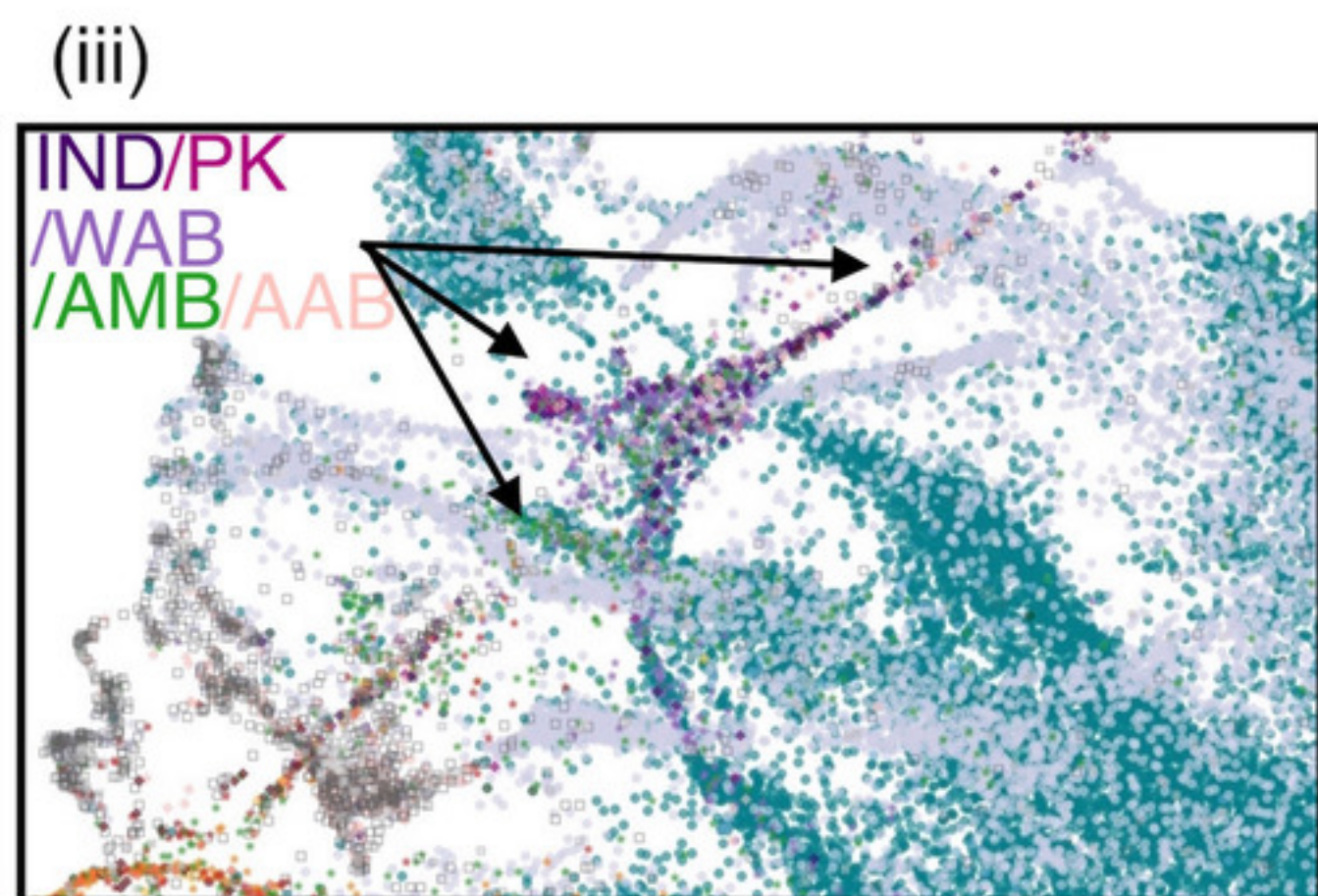

Supplement: S49 Fig — Zoomed in areas of Fig 3B. Sections (i) and (ii) respectively focus on the African and Asian superpopulations, and section (iii) focuses on an area with individuals from many ethnic backgrounds. Noticeable clusters of unidentified ethnic backgrounds appear and are labelled “OEG” (“Other Ethnic Group”). (PDF) [file pgen.1008432.s049.pdf]

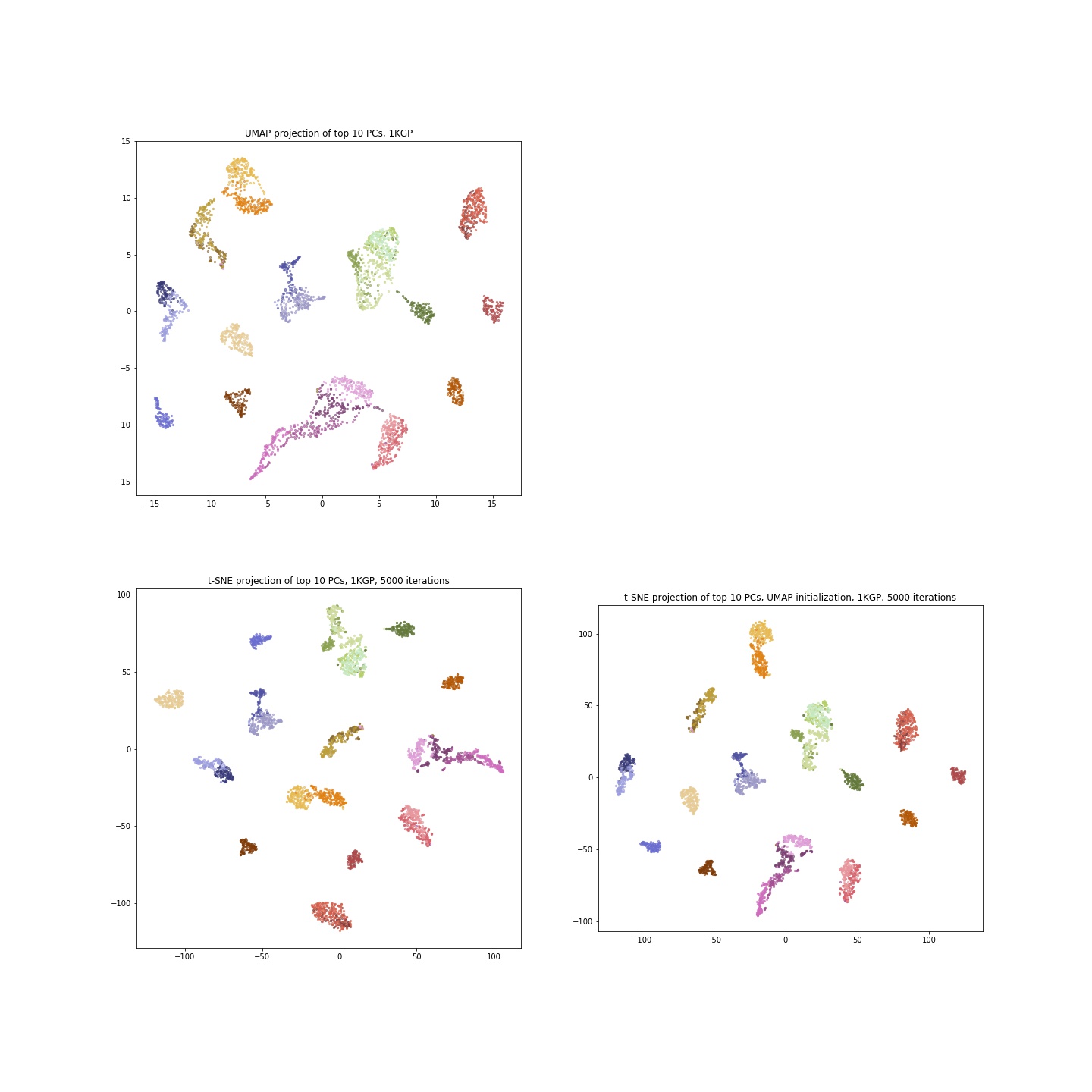

Supplement: S50 Fig — Comparing the visualizations of UMAP, standard t-SNE, and t-SNE initialized with a UMAP projection, on the top 10 principal components of the 1KGP. t-SNE used 5000 iterations. Initializing t-SNE with UMAP breaks the continuous structure of the projection and instead forms many small clusters. (JPEG) [file pgen.1008432.s050.jpeg]

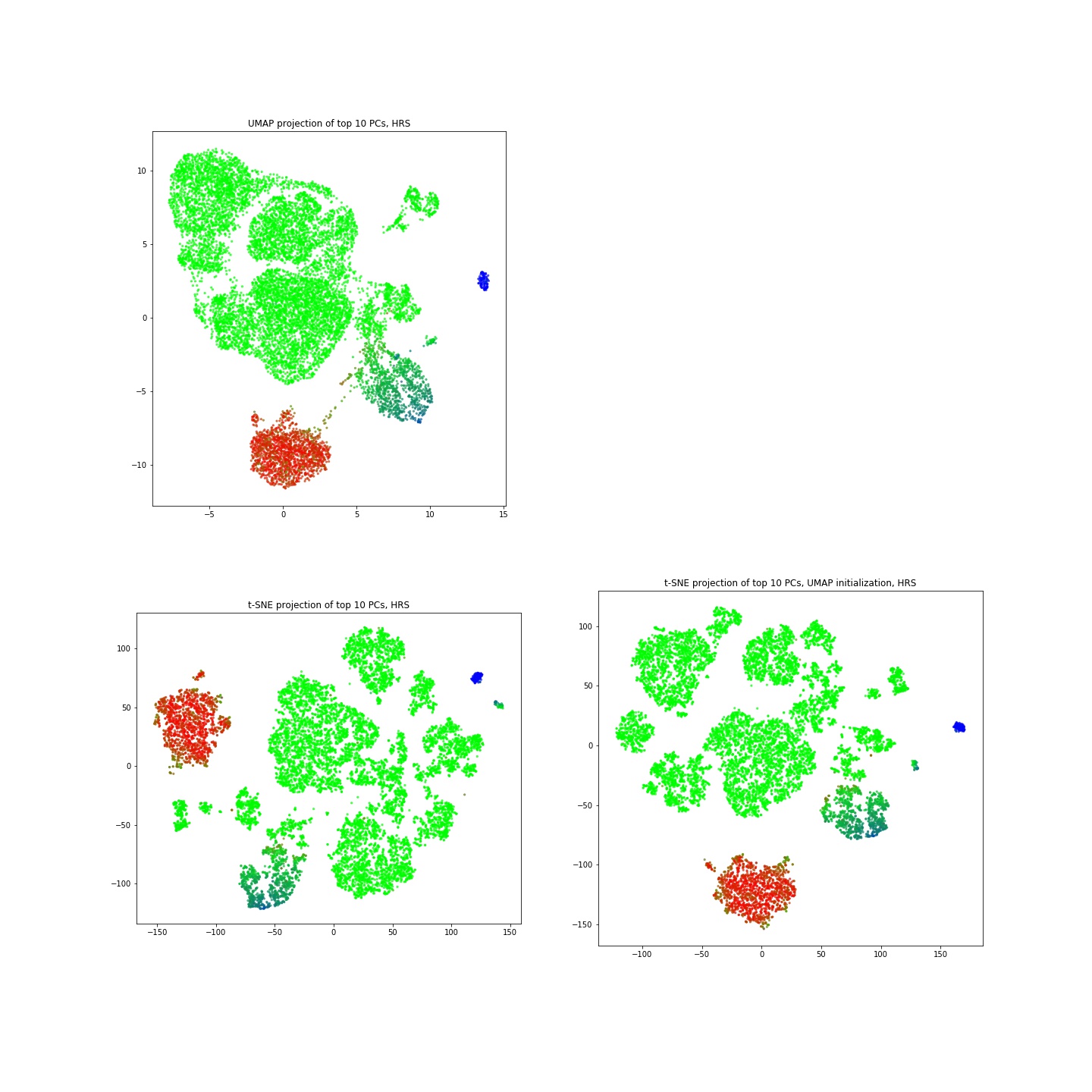

Supplement: S51 Fig — Comparing the visualizations of UMAP, standard t-SNE, and t-SNE initialized with a UMAP projection, on the top 10 principal components of the HRS. t-SNE used 5000 iterations. (JPEG) [file pgen.1008432.s051.jpeg]

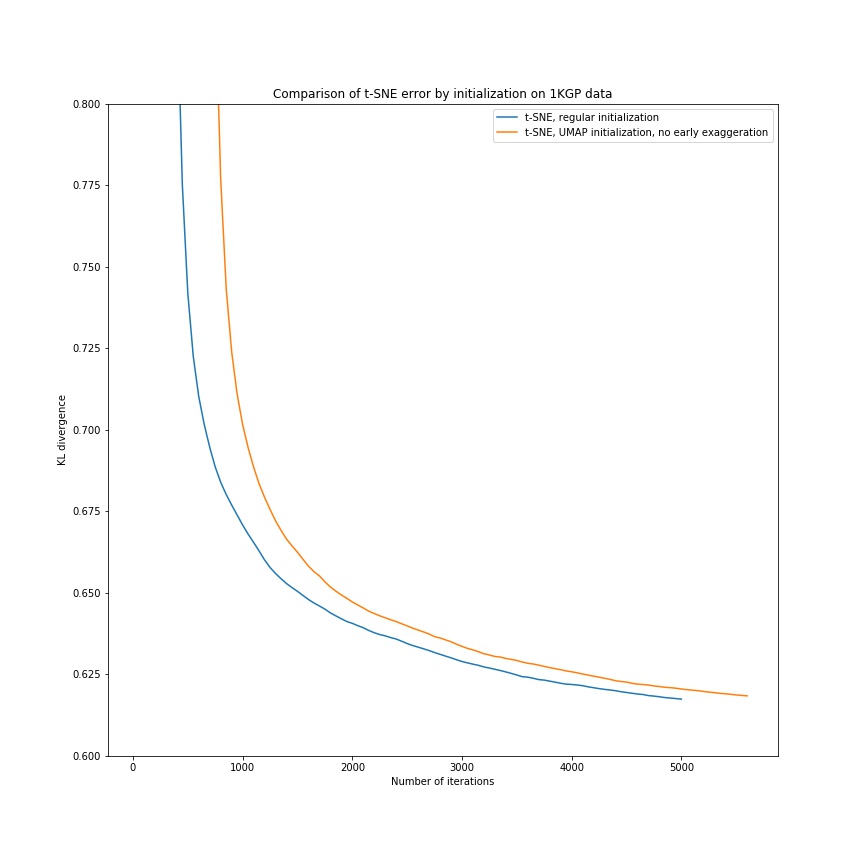

Supplement: S52 Fig — Comparing the error terms of standard t-SNE versus t-SNE initialized with a UMAP embedding and no early exaggeration. Done on the 1KGP dataset with 5000 iterations. The UMAP-initialized graph has been shifted by 600 iterations to approximate the 600 epochs UMAP uses for small datasets (n <= 10, 000). (JPEG) [file pgen.1008432.s052.jpeg]

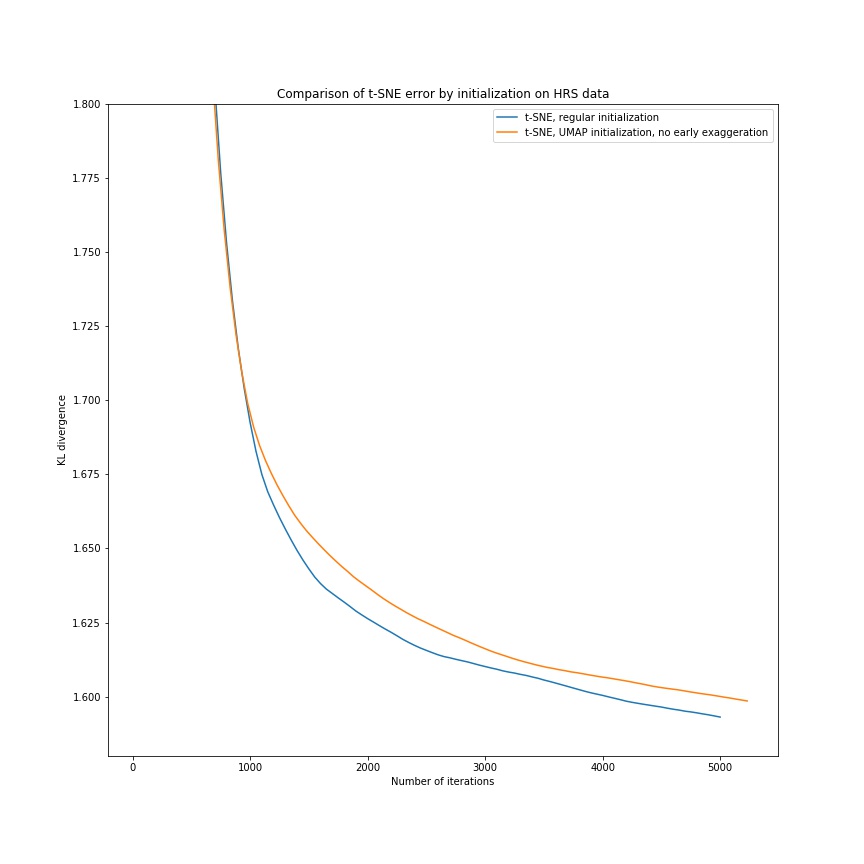

Supplement: S53 Fig — Comparing the error terms of standard t-SNE versus t-SNE initialized with a UMAP embedding and no early exaggeration. Done on the HRS dataset with 5000 iterations. The UMAP-initialized graph has been shifted by 230 iterations to approximate the 230 epochs UMAP uses for large datasets (n > 10,000). (JPEG) [file pgen.1008432.s053.jpeg]

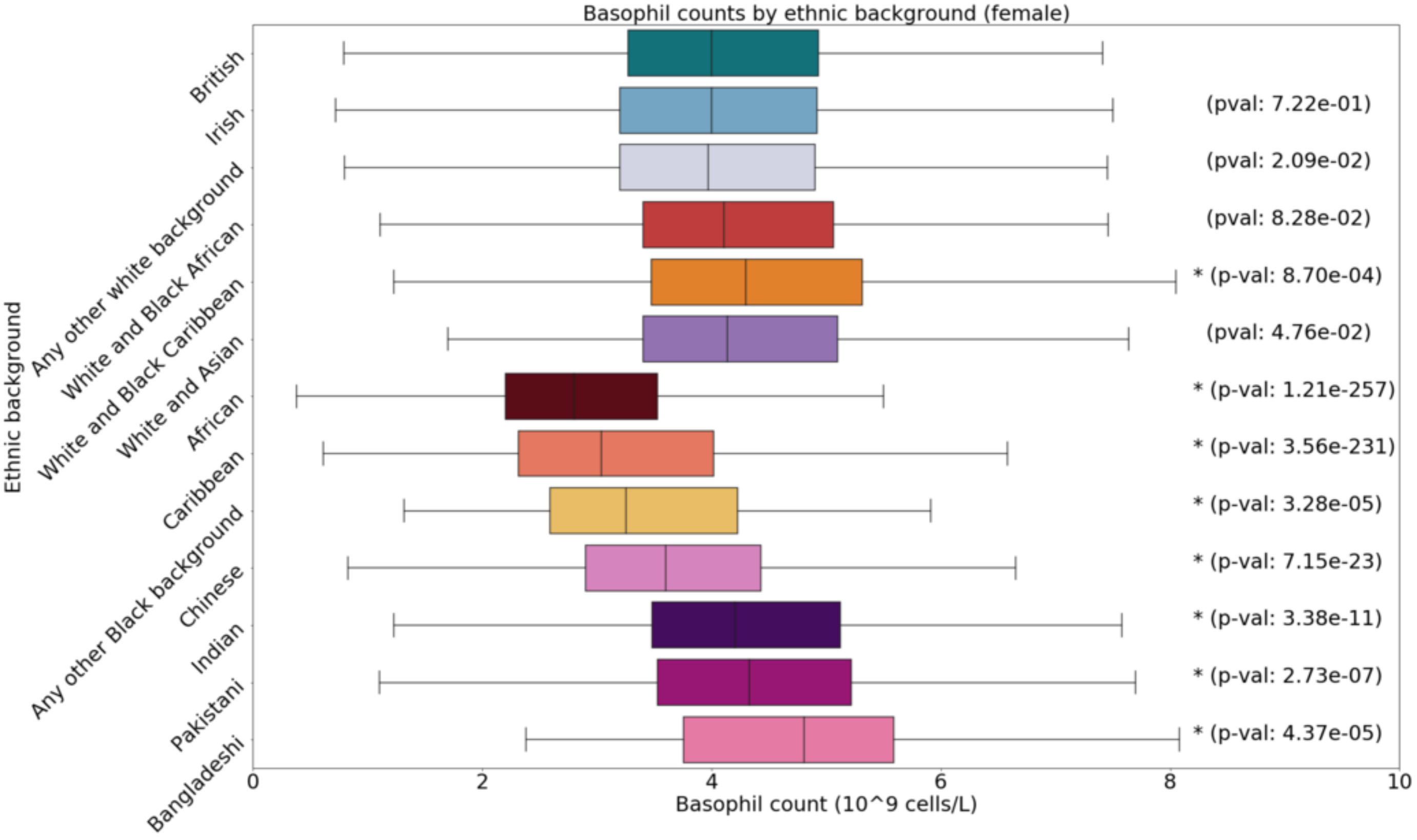

Supplement: S54 Fig — Basophil counts by sex and ethnic group, annotated with p-values. Asterisks indicate significant difference from the White British group with a Bonferroni correction for 12 groups. (PDF) [file pgen.1008432.s054.pdf]

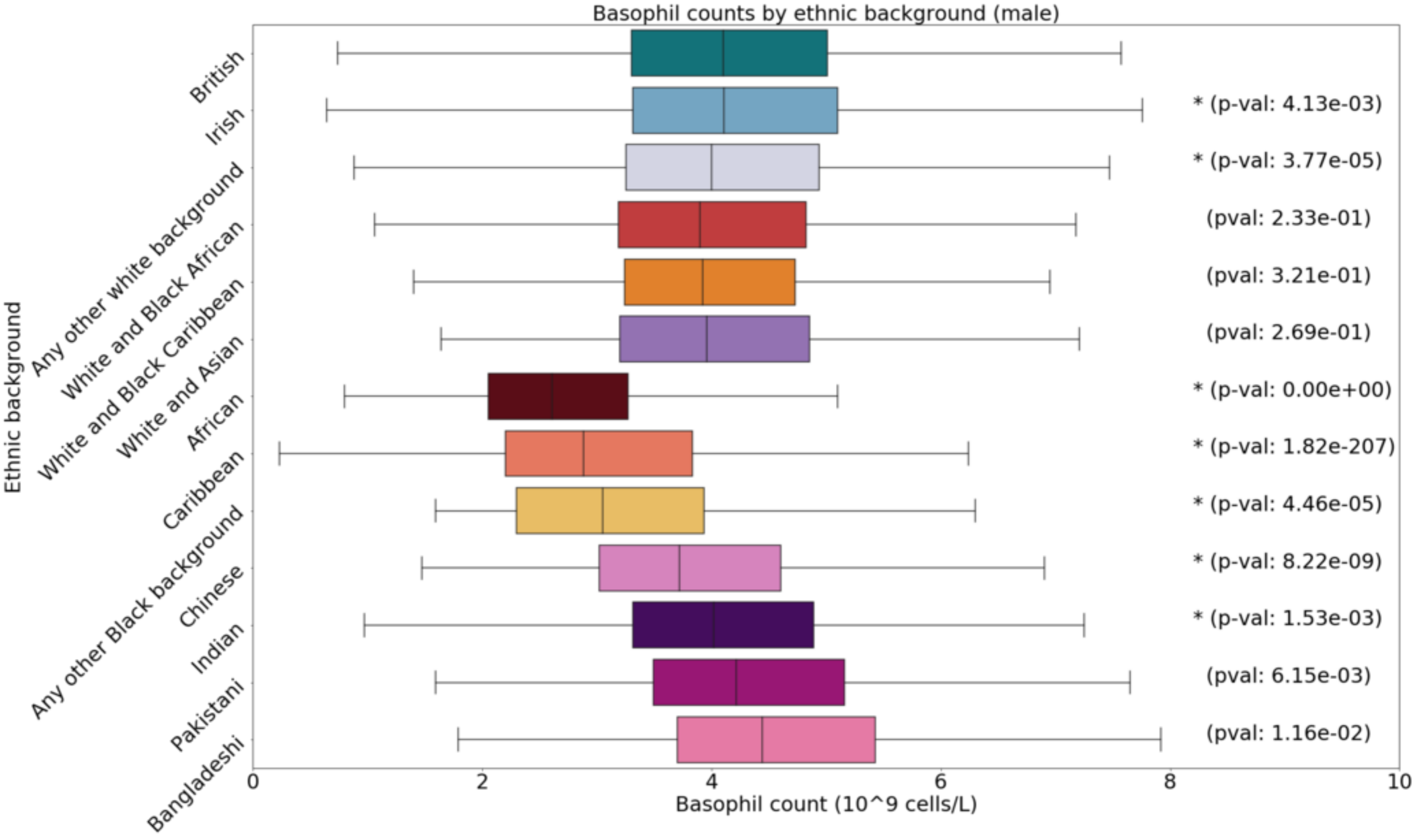

Supplement: S55 Fig — Basophil counts by sex and ethnic group, annotated with p-values. Asterisks indicate significant difference from the White British group with a Bonferroni correction for 12 groups. (PDF) [file pgen.1008432.s055.pdf]

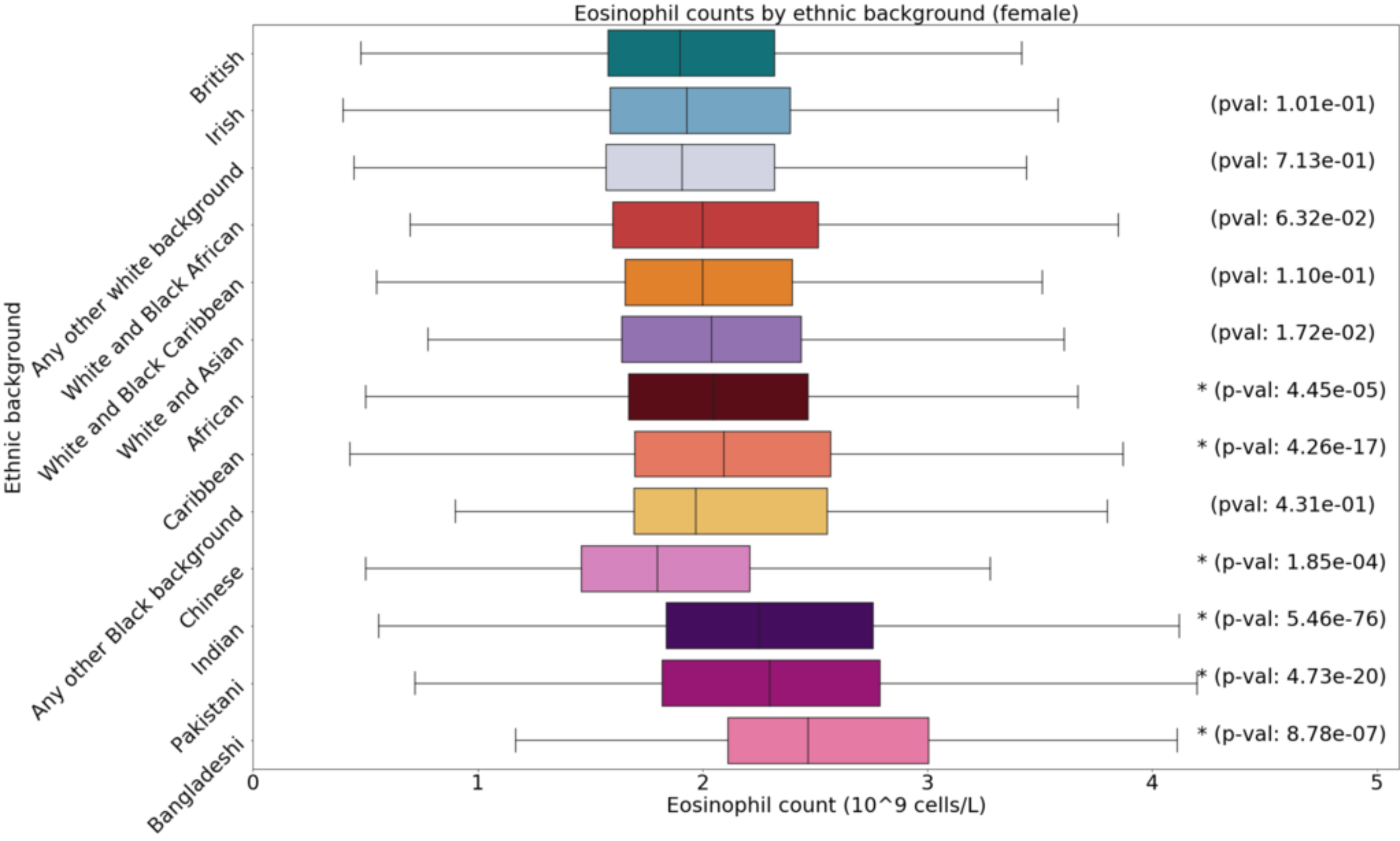

Supplement: S56 Fig — Eeosinophil counts by sex and ethnic group, annotated with p-values. Asterisks indicate significant difference from the White British group with a Bonferroni correction for 12 groups. (PDF) [file pgen.1008432.s056.pdf]

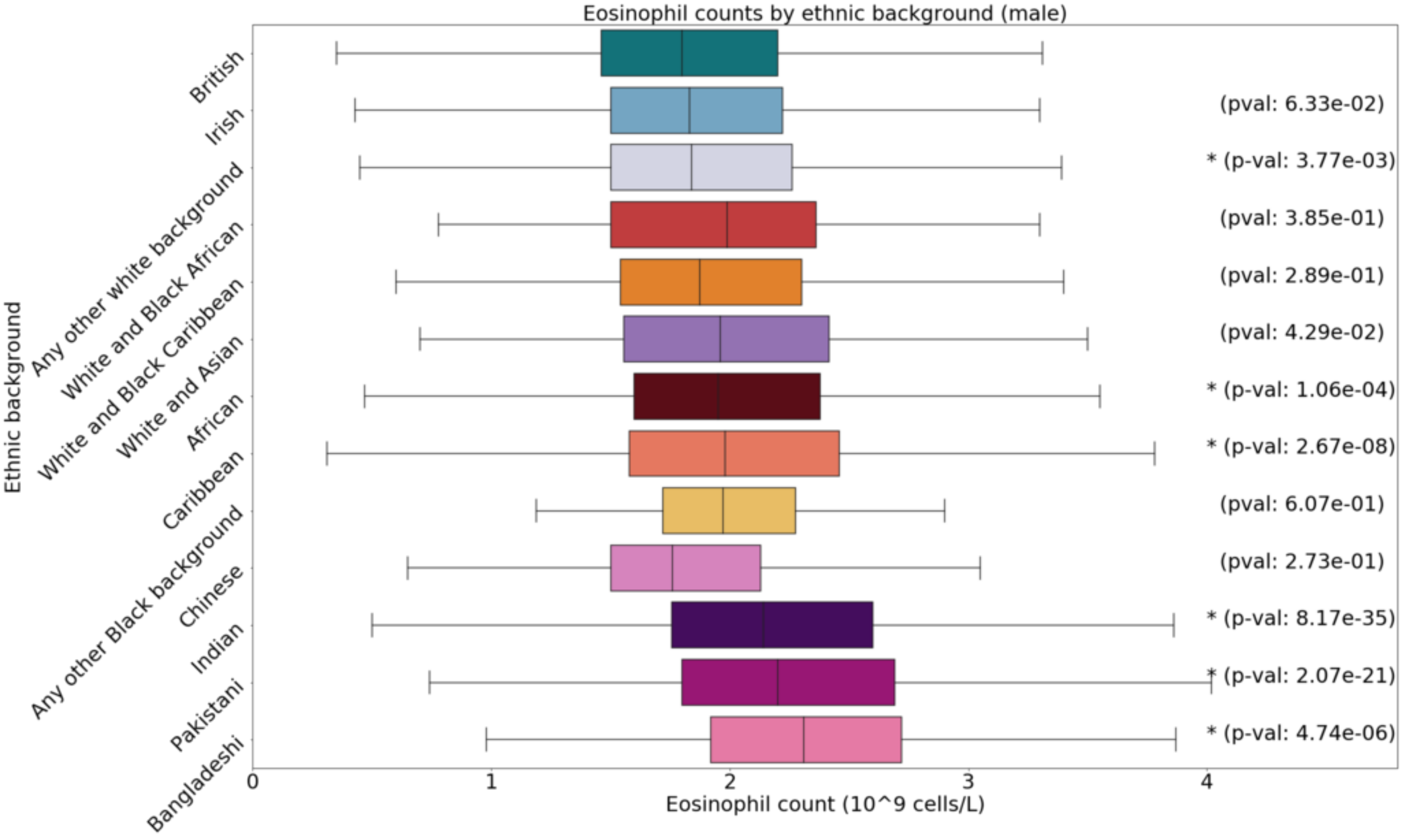

Supplement: S57 Fig — Eosinophil counts by sex and ethnic group, annotated with p-values. Asterisks indicate significant difference from the White British group with a Bonferroni correction for 12 groups. (PDF) [file pgen.1008432.s057.pdf]

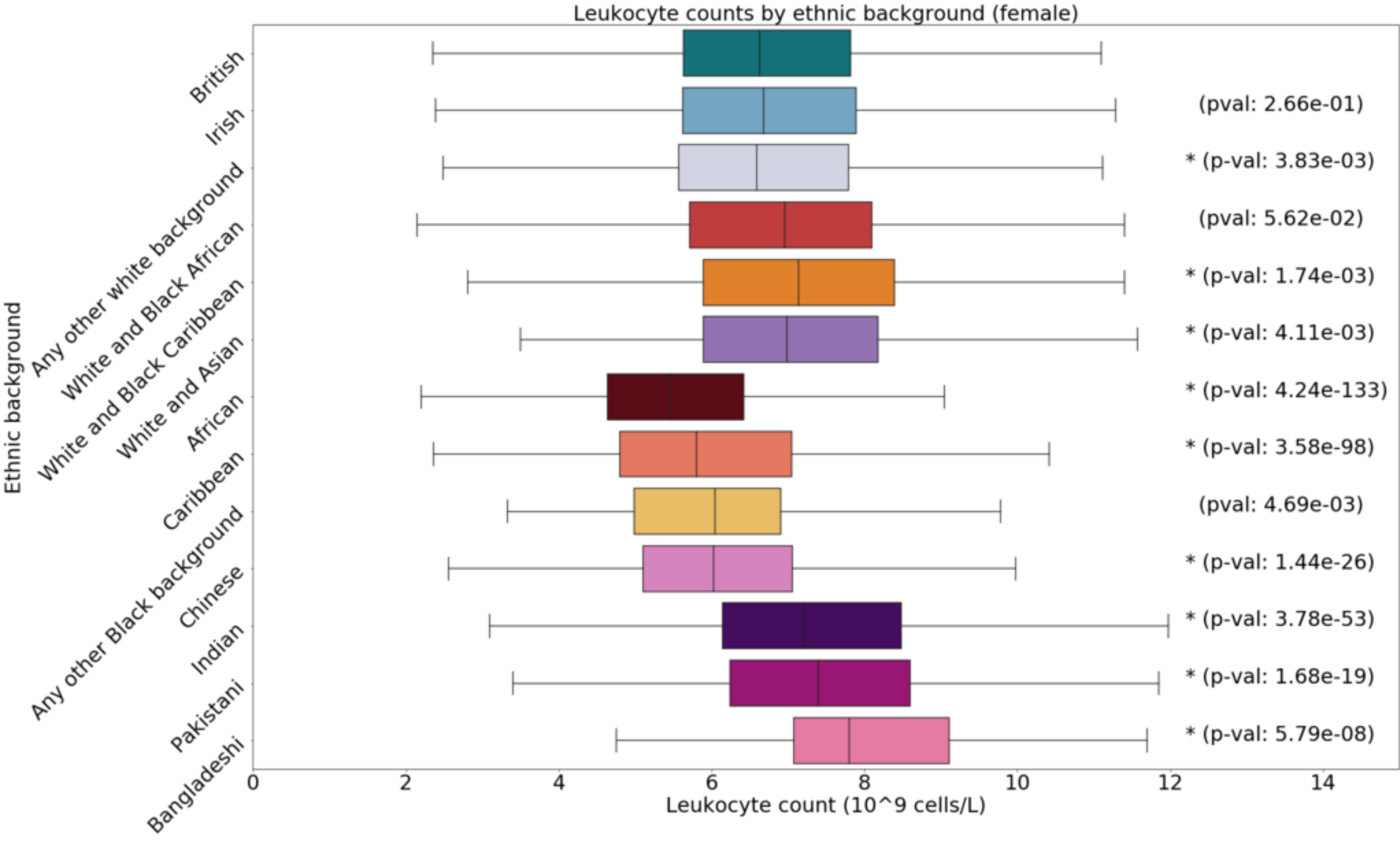

Supplement: S58 Fig — Leukocyte counts by sex and ethnic group, annotated with p-values. Asterisks indicate significant difference from the White British group with a Bonferroni correction for 12 groups. (PDF) [file pgen.1008432.s058.pdf]

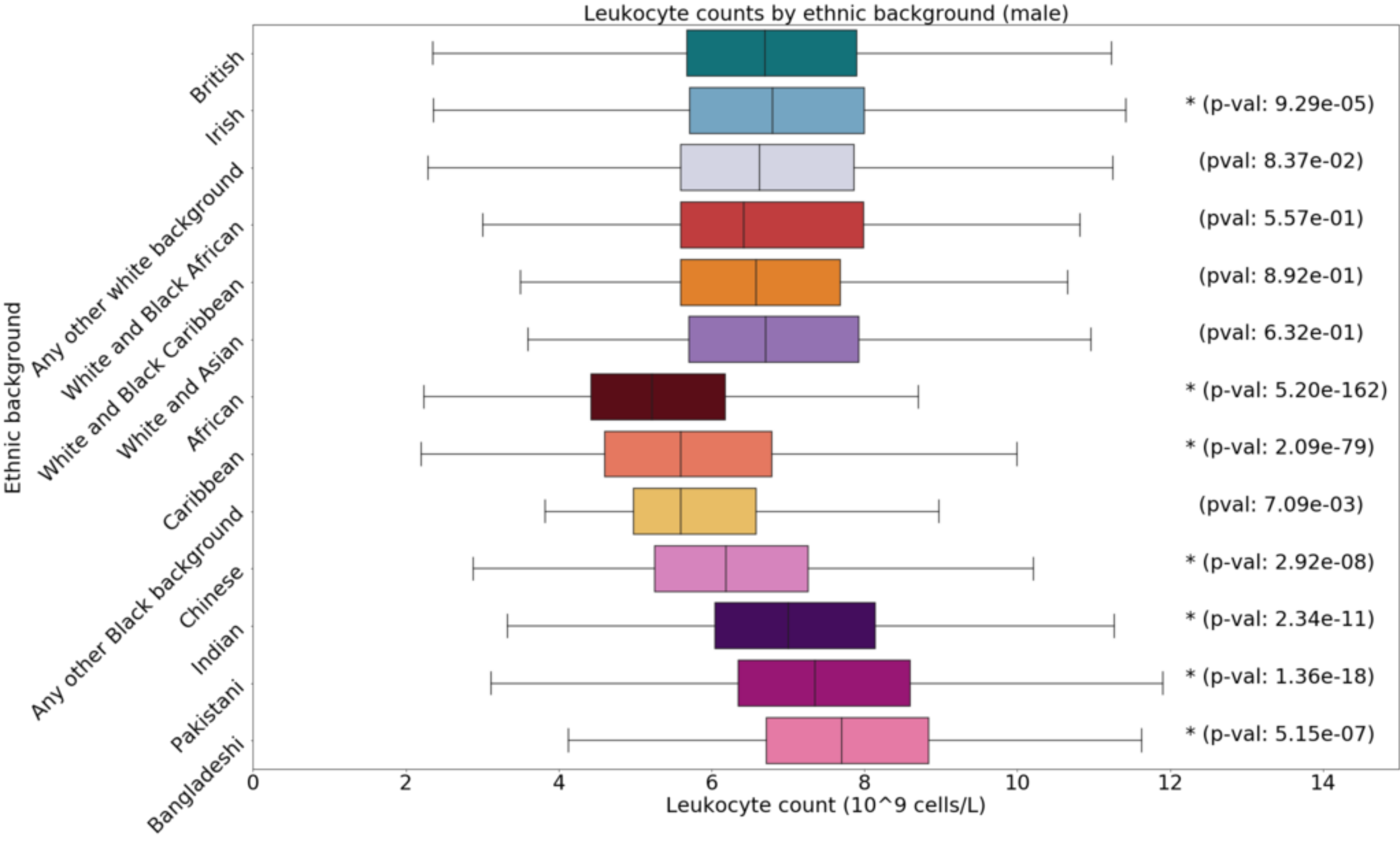

Supplement: S59 Fig — Leukocyte counts by sex and ethnic group, annotated with p-values. Asterisks indicate significant difference from the White British group with a Bonferroni correction for 12 groups. (PDF) [file pgen.1008432.s059.pdf]

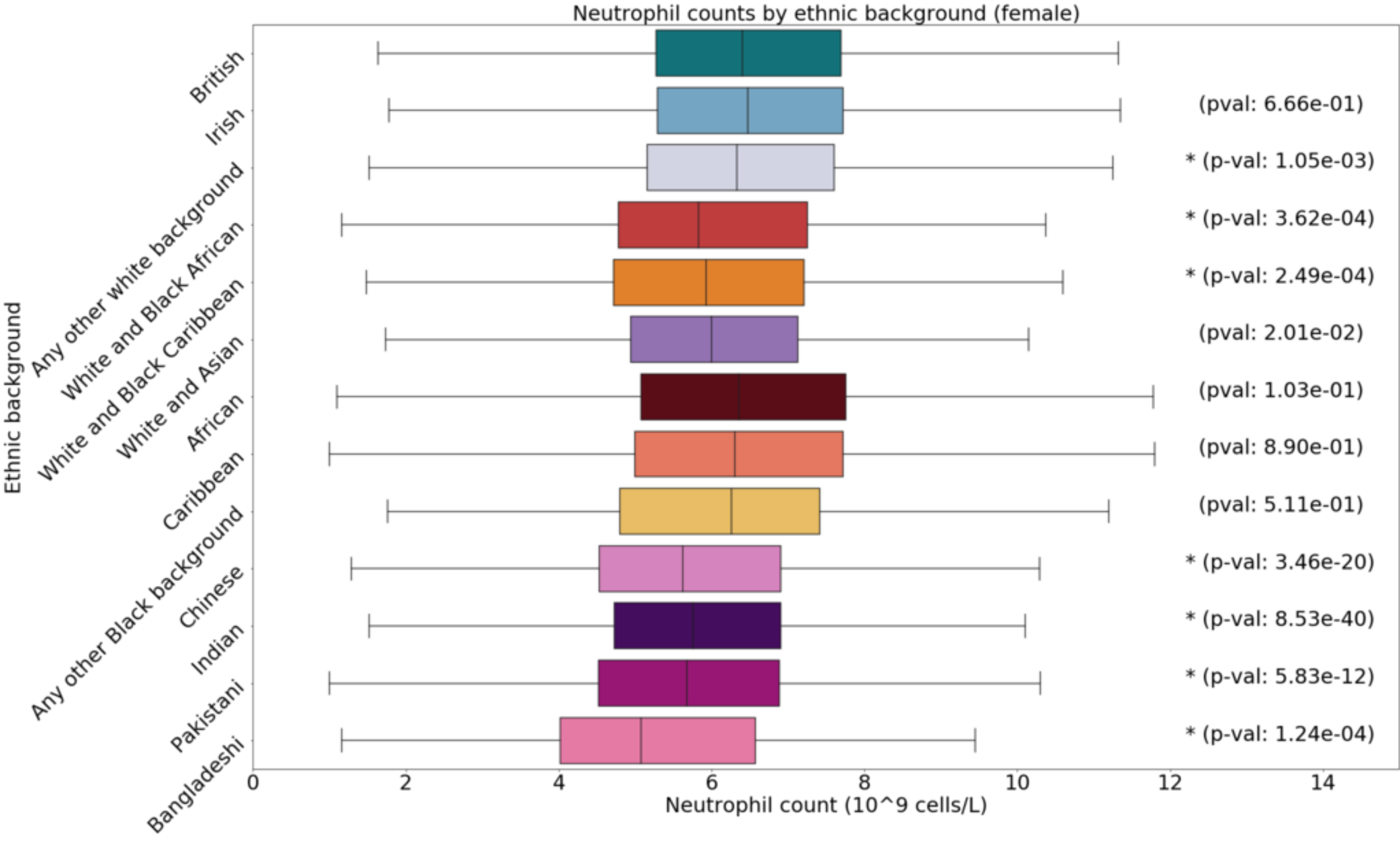

Supplement: S60 Fig — Neutrophil counts by sex and ethnic group, annotated with p-values. Asterisks indicate significant difference from the White British group with a Bonferroni correction for 12 groups. (PDF) [file pgen.1008432.s060.pdf]

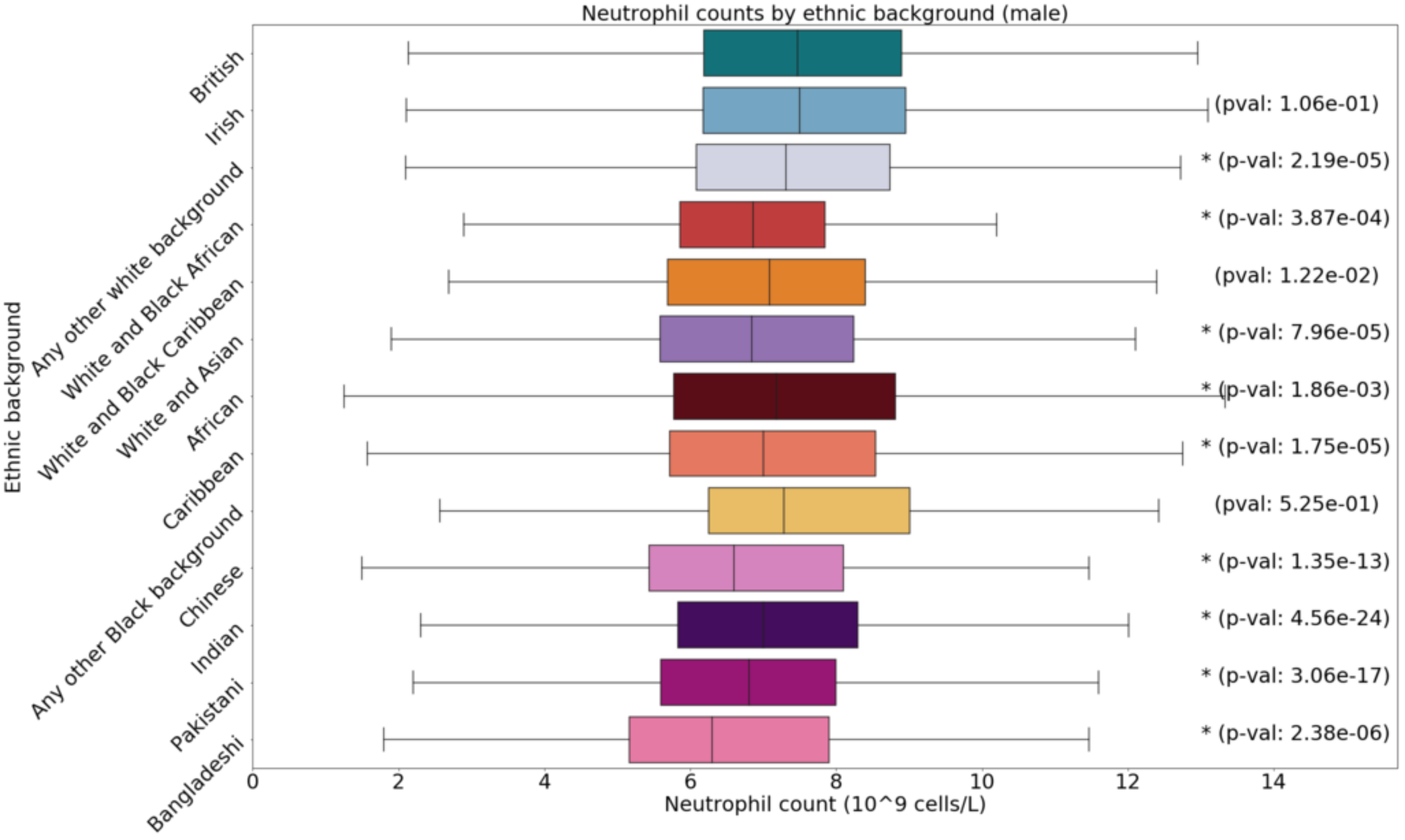

Supplement: S61 Fig — Neutrophil counts by sex and ethnic group, annotated with p-values. Asterisks indicate significant difference from the White British group with a Bonferroni correction for 12 groups. (PDF) [file pgen.1008432.s061.pdf]

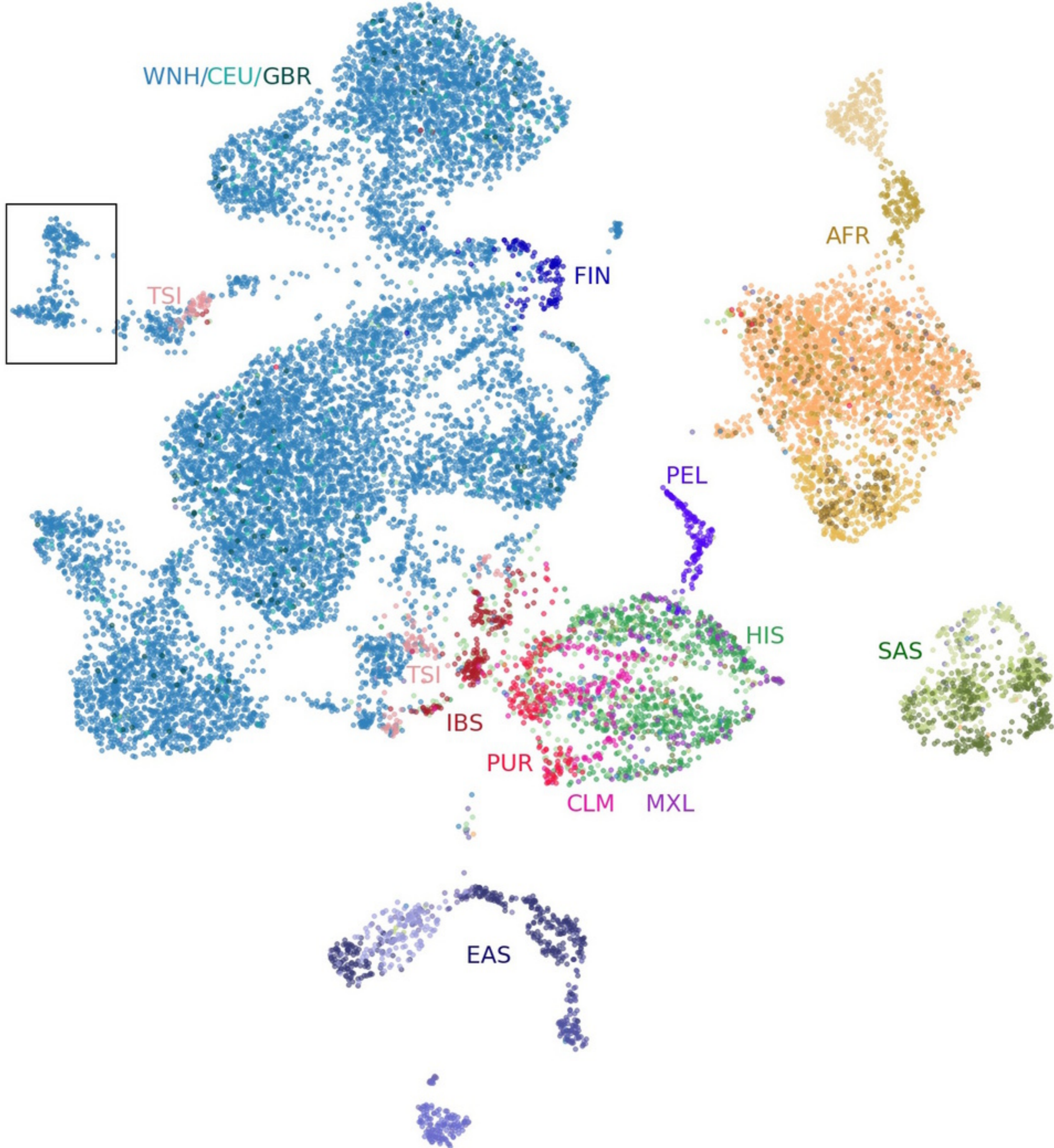

Supplement: S62 Fig — UMAP projection of the top 10 principal components of the combined HRS and 1KGP datasets. One cluster (in the box) does not group with any of the 1KGP populations. A cluster of Finnish (FIN) individuals consistently appears in the “White Not Hispanic” (WNH) group. Groups of Central and South American populations from the 1KGP (CLM, Colombian; MXL, Mexican; PEL, Peruvian; PUR, Puerto Rican) form nearby or within the HRS Hispanic cluster (HIS). Iberian individuals (IBS) cluster near the Hispanic population. Toscani individuals (TSI) form some small clusters and sometimes appear near the Iberian and Hispanic populations. Individuals with British/Scottish (GBR) or Northern/Western European ancestry (CEU) are scattered throughout the WNH clusters. Individuals with African ancestry from the 1KGP group with Black Americans from the HRS (AFR). Similar population groupings occur with South Asian (SAS) and East Asian (EAS) individuals. (PDF) [file pgen.1008432.s062.pdf]

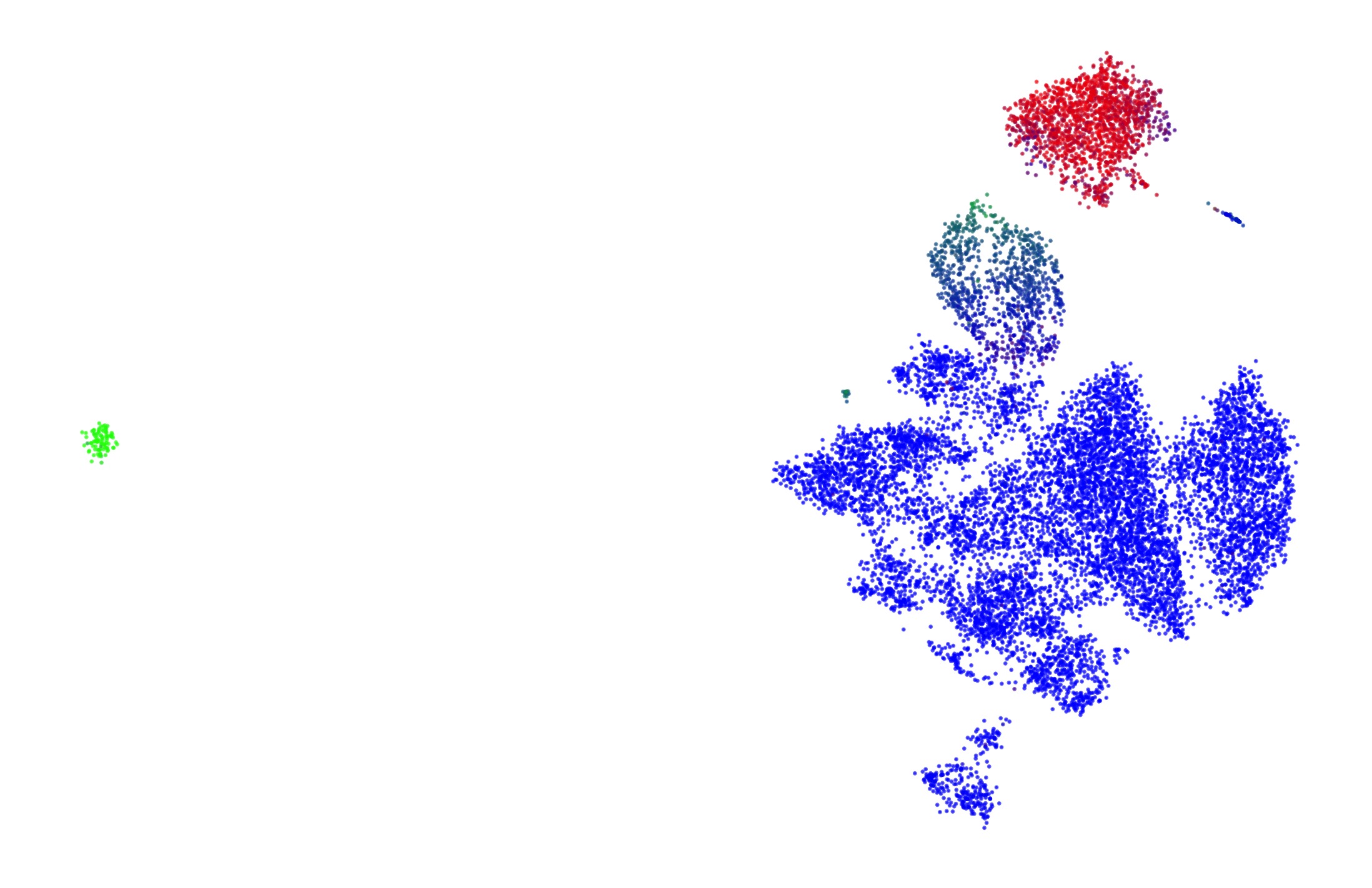

Supplement: S63 Fig — An alternate colouring of S7 Fig. Here red, green, and blue correspond to African, Asian/Native American, and European ancestry, respectively. (JPEG) [file pgen.1008432.s063.jpeg]

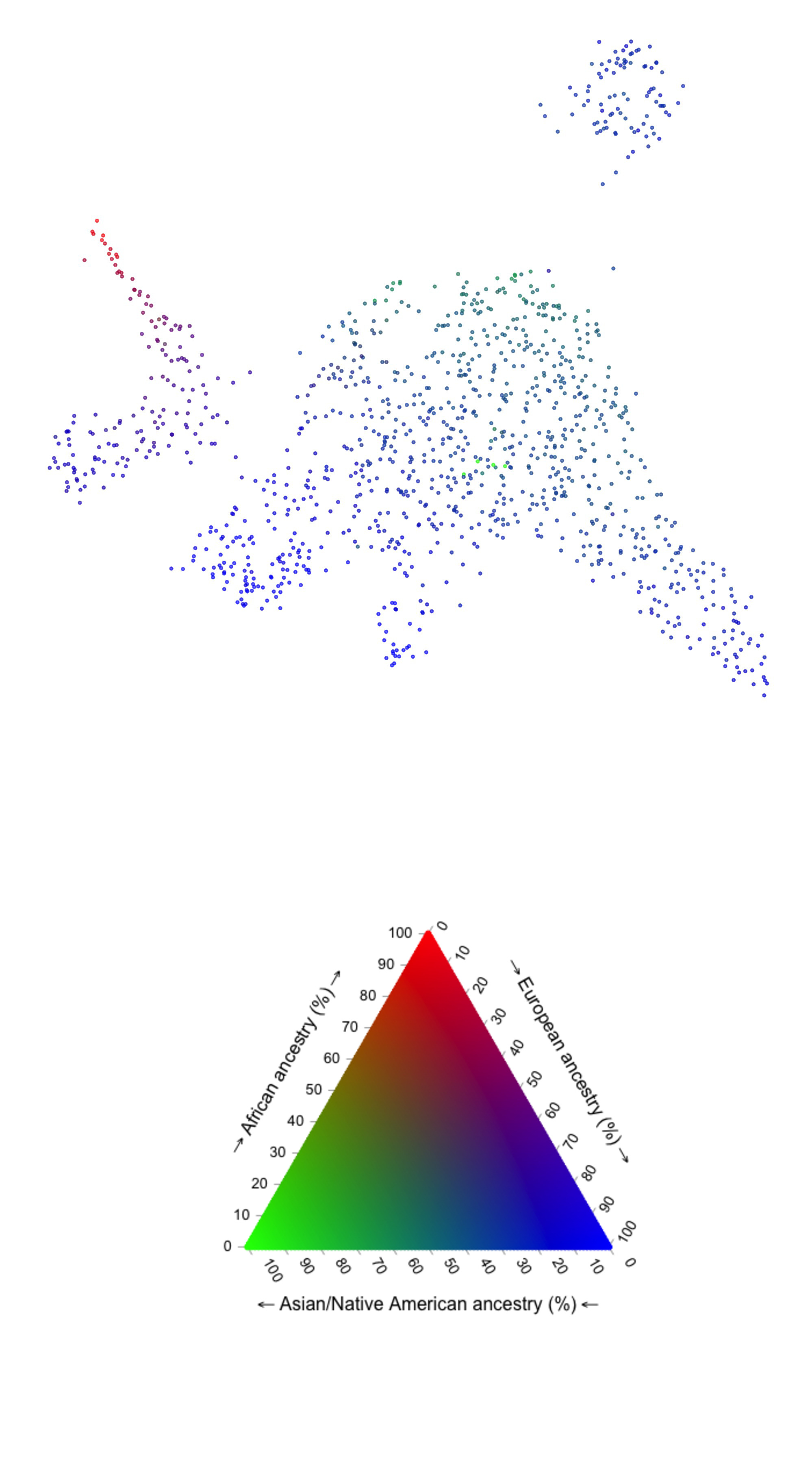

Supplement: S64 Fig — An alternate colouring of S11 Fig. Here red, green, and blue correspond to African, Asian/Native American, and European ancestry, respectively. (JPEG) [file pgen.1008432.s064.jpeg]

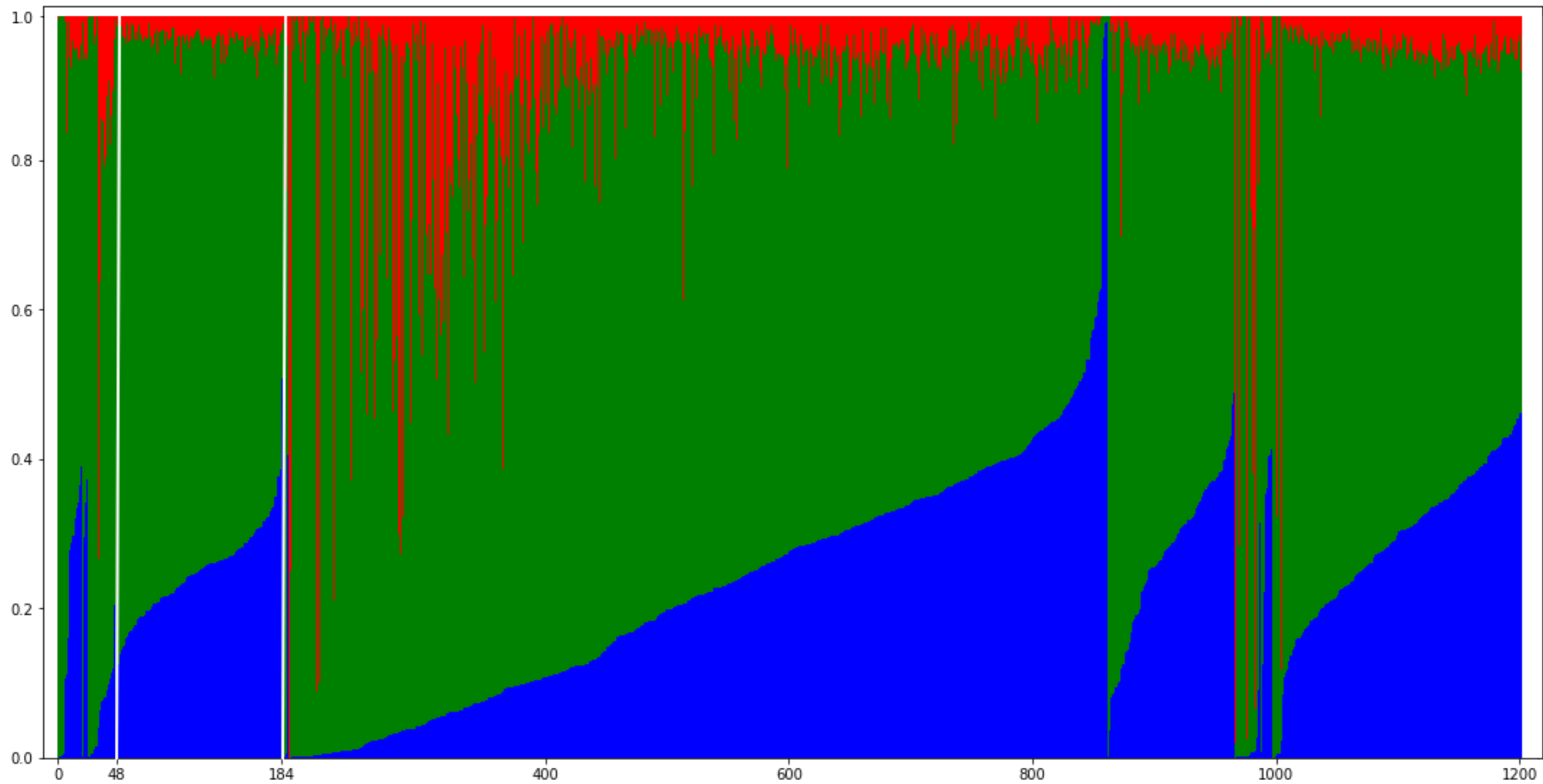

Supplement: S65 Fig — Admixture plot of Hispanic individuals in the HRS. Individuals born in the Mountain census region fall between the white lines (indices 48 to 184). (PDF) [file pgen.1008432.s065.pdf]
