# Supplementary material for: UMAP reveals cryptic population structure and phenotype heterogeneity in large genomic cohorts
Source: PLoS Genet. 2019 Nov 1;15(11):e1008432. doi: 10.1371/journal.pgen.1008432 (PMC6853336; doi:10.1371/journal.pgen.1008432)
Supplement: S6 Fig — UMAP applied to the first 10 principal components of HRS data. Points coloured by self-identified race, Hispanic status, and Mexican-American status. The cluster on the left is mostly people who identify as neither Black nor White and were born outside the contiguous United States or in the Pacific census region. Clustering with the 1KGP data places them with Asian-identified populations. BNH, Black (not Hispanic); BHO, Black (Hispanic, Other); WNH, White (not Hispanic); WHM, White (Hispanic, Mexican-American); WHO, White Hispanic (Other); ONH, Other (not Hispanic); OHM, Other (Hispanic, Mexican-American); OHO, Other (Hispanic, Other). (PDF) [file pgen.1008432.s006.pdf]

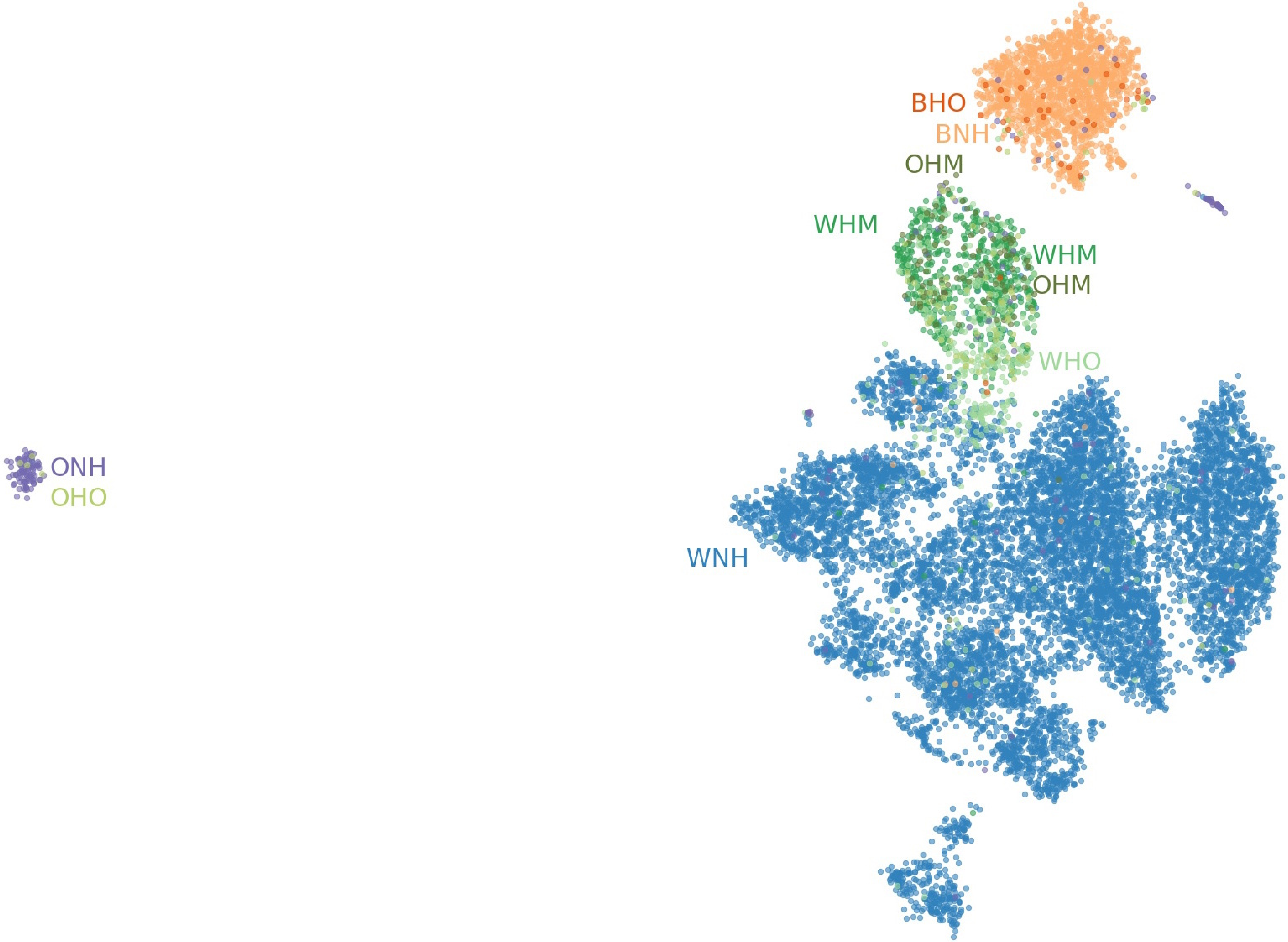
BHO
BNH
OHM
WHM
WHM
OHM
WHO
ONH
OHO
WNH
