# Supplementary material for: UMAP reveals cryptic population structure and phenotype heterogeneity in large genomic cohorts
Source: PLoS Genet. 2019 Nov 1;15(11):e1008432. doi: 10.1371/journal.pgen.1008432 (PMC6853336; doi:10.1371/journal.pgen.1008432)
Supplement: S1 Table — Variance explained in the 1KGP data by the number of principal components used. (PDF) [file pgen.1008432.s066.pdf]

| Number of PCs | Variance explained |
|---|---|
| 2 | 13.6% |
| 3 | 14.7% |
| 4 | 15.5% |
| 5 | 15.7% |
| 6 | 15.8% |
| 7 | 15.9% |
| 8 | 16.0% |
| 9 | 16.1% |
| 10 | 16.2% |
| 11 | 16.2% |
| 12 | 16.3% |
| 13 | 16.4% |
| 14 | 16.5% |
| 15 | 16.5% |
| 30 | 17.5% |
| 50 | 18.6% |
| 100 | 21.3% |
| 200 | 26.4% |
| 300 | 31.1% |
| 400 | 35.5% |
| 500 | 39.7% |
| 600 | 43.7% |
| 700 | 47.4% |
| 800 | 50.8% |
| 900 | 54.0% |
| 1000 | 57.0% |
| 1100 | 60.0% |
| 1200 | 62.8% |
| 1300 | 65.4% |
| 1400 | 68.0% |
| 1500 | 70.5% |
| 1600 | 73.0% |
| 1700 | 75.3% |
| 1800 | 77.5% |
| 1900 | 79.7% |
| 2000 | 81.8% |
| 2100 | 83.8% |
| 2200 | 85.8% |
| 2300 | 87.6% |
| 2400 | 89.4% |
| 2500 | 91.1% |
| 2600 | 92.7% |
| 2700 | 94.2% |
| 2800 | 95.3% |
| 2900 | 96.3% |
| 3000 | 97.1% |
| 3100 | 97.8% |
| 3200 | 98.5% |
| 3300 | 99.2% |
| 3400 | 99.7% |

Table 1: Variance explained in the 1KGP data by the number of principal components used.
